# Supplementary material for: Child and family-focused interventions for child maltreatment and domestic abuse: development of core outcome sets
Source: BMJ Open. 2022 Sep 19;12(9):e064397. doi: 10.1136/bmjopen-2022-064397 (PMC9486347; doi:10.1136/bmjopen-2022-064397)
Supplement: Supplementary data [file bmjopen-2022-064397supp001.pdf]

Online only supplementary material

Supplement 1: Sensitivity protocol 2

Supplement 2: Characteristics of participants in the identification (Stage 1) and development (Stage 2) of core outcomes for CM and DVA 3

Supplement 3: Table of inclusion criteria for rapid evidence reviews 6

Supplement 4: Rapid evidence review flowcharts 12

Supplement 5: List of websites searched in grey literature review 13

Supplement 6: Overview of e-Delphi survey questions 15

- Round 1: Example question 15
- Round 2: Example question 15
- Round 3: Example question 15

Supplement 7: List of changes to the protocol 16

Supplement 8: Rating of e-Delphi survey outcomes 17

- Round 1 scoring: Child maltreatment and domestic violence and abuse 17
- Round 2 and 3 scoring: Child maltreatment 18
- Round 2 and 3 scoring: Domestic violence and abuse 22

Supplement 9: DVA consensus workshop consensus statement 26

Supplement 10: Consensus workshop outcome rankings 27

Supplement 11: Summary of discussion about implications of COS for underserved groups 28

Supplement 1: Sensitivity protocol

Before the interview

- Ask for permission to let their support organisation know that they're take part
- Offer for interviewee to bring someone to the interview if they want to
- Ask if there's anything that would help make them feel comfortable
- Offer a choice of venue if possible
- Offer possibility that participants can carry interview out by email
- Recap the aim of the interview/research and who is doing the research

During the interview

- Have a box of tissues but not visibly out
- Ask 'are you happy to talk about this?' and skip to the next question if the answer is no
- Allow pauses during the interview – don't rush to fill silences
- Offer break(s) if necessary or to do the interview in two parts
- Allow interviewees chance to go off-topic

After the interview

- Give a list of support options at the end of the interview
- Give option that interviewee can feedback or give more details
- Researcher to send a text message for follow-up and offer a call
- Explain clearly next steps e.g. referrals

Supplement 2: Characteristics of participants in the identification (Stage 1) and development (Stage 2) of core outcomes for CM and DVA

STAGE ONE

Month 3

Domestic violence and abuse stakeholder workshop held on 19th June 2019 at Friends House, Euston, London		
Survivors	Practitioners	Researchers
10 survivors attended from:<br>• University of Central Lancashire Survivor Researcher Advisory Group<br>• National Survivor User Network (NSUN)<br>• Violence, Abuse and Mental Health Network (VAMHN)	25 practitioners and policymakers attended from:<br>• Frontline and second tier DVA third sector organisations<br>• Local authority service commissioners, government ministries<br>• Schools and education providers	10 researchers and 14 speakers and facilitators attended from:<br>• UK universities and research groups<br>• International universities and research groups

Month 6

Child maltreatment stakeholder workshop held on 5th September 2019 at Institute of Child Health, University College London, London		
Survivors	Practitioners	Researchers
9 survivors attended from:<br>• University of Central Lancashire Survivor Researcher Advisory Group<br>• National Survivor User Network (NSUN)<br>• Violence, Abuse and Mental Health Network (VAMHN)	16 practitioners and policymakers attended from:<br>• Frontline and second tier CM third sector organisations<br>• Local authorities, government ministries<br>• Health service providers and commissioners<br>• Social care professionals and associated organisations	6 researchers and 10 speakers and facilitators attended from:<br>• UK universities and research groups

Months 17 to 21

Qualitative survivor interviews held online from August to December 2020 (N=10)	
Demographics	n (%)
Age	
18 to 35 years	2 (20)
36 to 45 years	2 (20)
46+ years	5 (50)
Unknown	1 (20)
Sex	
Female	8 (80)
Male	2 (20)
Ethnicity	
Asian-European	2 (20)
Black British/Caribbean	2 (20)
White British/UK	5 (50)
Other	1 (10)
Type of abuse	
Child maltreatment only	0 (0)
Domestic violence only	5 (50)
Both	5 (50)

STAGE TWO

Months 26 to 30

E-Delphi surveys held online from May to Sept 2021 (N=170)<br>*CM and DVA participants amalgamated for anonymity*	
Demographics	n (%)
Panel	
Survivors	42 (25)
Practitioners	66 (39)
Researchers	62 (36)
Age	
18 to 25 years	2 (1)
26 to 35 years	21 (12)
36 to 45 years	49 (29)
46 to 55 years	42(25)
56 to 65 years	44 (26)
66+ years	12 (7)
Sex / gender	
Female	146 (86)
Male	22 (13)
*Non-binary / third gender / Trans**	2 (2)
Ethnicity	
Asian or Asian British (Indian, Pakistani, Bangladeshi, Chinese, Any other Asian background)	10 (6)
Black / African / Caribbean / Black British	6 (4)
Mixed / multiple ethnic groups	16 (9)
White (English, Welsh, Scottish, Northern Irish, British, Irish, Gypsy or Irish Traveller, Any other White background)	137 (81)
Type of abuse (survivors only)	
Child maltreatment only	8 (19)
Domestic violence only	9 (21)
Both	25 (60)

Month 30

Child maltreatment consensus workshop held online on 23rd September 2021		
Survivors	Practitioners	Researchers
6 survivors attended from:<br>• University of Central Lancashire Survivor Researcher Advisory Group<br>• National Survivor User Network (NSUN)<br>• Violence, Abuse and Mental Health Network (VAMHN)<br>• E-Delphi survey participation	*10 practitioners and policymakers attended from:*<br>• Frontline and second tier CM third sector organisations<br>• Local authorities, government ministries<br>• Health service providers and commissioners<br>• Social care professionals and associated organisations	*4 researchers and 4 facilitators attended from:*<br>• UK universities and research groups<br>• International universities and research groups

Month 30

Domestic violence consensus workshop held online on 28th September 2021		
Survivors	Practitioners	Researchers
5 survivors attended from:<br>• University of Central Lancashire Survivor Researcher Advisory Group<br>• National Survivor User Network (NSUN)<br>• Violence, Abuse and Mental Health Network (VAMHN)<br>• E-Delphi survey participation	*13 practitioners and policymakers attended from:*<br>• Frontline and second tier DVA third sector organisations<br>• Local authorities, government ministries<br>• Health service providers and commissioners<br>• Social care professionals and associated organisation<br>• Police	*5 researchers and 4 facilitators attended from:*<br>• UK universities and research groups

BMJ Publishing Group Limited (BMJ) disclaims all liability and responsibility arising from any reliance placed on this supplemental material which has been supplied by the author(s)

Supplement 3: Table of inclusion criteria for rapid evidence reviews

Review	Criteria	Inclusion criteria	Exclusion criteria
Review of systematic review	Study type	Peer-reviewed systematic reviews of controlled or quasi experimental comparator intervention studies: with or without randomisation.<br><br>The DARE criteria for SRs are at least 4 of the following: reporting of inclusion/exclusion criteria; adequate search; synthesis of included studies; quality assessment of studies; sufficient detail presented (CRD, 1995). For the purposes of this review, SRs will be included if they use an electronic database and have a structured search strategy.<br><br>Published since 2014.<br><br>No restrictions by country. English language only.<br><br>Individual studies must include DVA/CM in one of the following ways:<br><br>o Entry to the intervention is determined by experience, perpetration or identified as at risk of DVA/CM. (Identification of risk is by researchers, practitioners, or participants thus we do not have a definition)<br><br>o Subgroup analysis is carried out of participants who have experienced (or are considered to be at risk of) DVA/CM<br><br>o DVA/CM is measured as an exposure (this could be retro or prospectively reported)	Non peer-reviewed studies<br><br>Qualitative studies<br><br>General literature reviews<br><br>Protocols<br><br>Case reports<br><br>Cross-sectional studies<br><br>General discussion papers<br><br>Letters<br><br>Commentaries<br><br>Book chapters<br><br>Conference papers<br><br>Theses and dissertations.
	Population	Children or families with children at risk of experiencing or experiencing DVA/CM. This includes unborn children, children (aged 0 to 18 years), designated as victim or witness.<br><br>For DVA any adult family members who have a parenting role (Early Intervention Foundation, 2014), whether designated as perpetrator, victim, witness, or household member.	

	For CM any adult family members who have a caring role, whether designated as maltreating parent, witness, or household member.<br><br>These adults and children could either be the primary study population of interest or form a subgroup in a wider study population.	
Intervention	Any interventions or services where:<br><br>Experience of or increased risk of experiencing DVA/CM is a criterion for being offered the service<br><br>OR<br><br>DVA/CM is measured as an exposure or outcome of interest<br><br>AND<br><br>At least one child or family-level outcome is measured. Family-level outcomes do not need to be explicitly labelled as 'family' level; we will make a judgement. However, they include any outcome that affects the family/household unit. For example, worklessness in study where at least some participants are reported to be parents would be included.<br><br>Studies must include evaluation of a defined activity/programme and evaluation of a hypothesised effect.<br><br>Interventions may be delivered to any family member(s) as an individual or in a group. Any duration of intervention will be included. Any setting will be considered.	Universal interventions that do not specifically target children and families at risk of<br><br>DVA/CM; targeted interventions that do not measure any child or family level outcomes e.g. perpetrator programmes that focus solely on attitudinal change; DVA (only) interventions focused solely on elder abuse, sibling abuse or child perpetration of domestic violence where participants have not been identified as exposed to DVA.
Comparator	Any control or comparison group/period with participants receiving no care, treatment as usual or any other treatment	

	Outcome	Any child outcome related to i) the child's experience of adversity ii) child functioning, including risky behaviours.<br><br>Any outcomes related to the quality of the caregiving environment (e.g. parenting, maternal depression, stressful life events, maternal psychological distress, parental substance misuse).<br><br>Any outcomes related to material deprivation e.g. low income, economic hardship, or stress (including perceived), social capital, hunger, food poverty, housing instability.<br><br>Any other outcome judged to relate to children or families by the research team.<br><br>Outcomes can be reported by professionals, child, parent or other family member and they can be retrospective or prospective.<br><br>Outcomes can be end points, surrogate markers for end points or intermediate outcomes.<br><br>No minimum or maximum follow-up is required.	
	Context	Studies from any country in any setting	
Review of qualitative studies	Study type	Primary qualitative (i.e. analysis of interviews, focus groups or other verbal analysis which is not quantified) intervention studies either as a standalone study or a discrete component of mixed method studies.<br><br>Direct and sufficient verbatim text from participants for analysis (i.e. more than two lines) c.f. Arai et al. (2019).<br><br>Published since October 2015 (DVA) and July 2014 (CM) to build on Howarth et al. (2016) and Macdonald et al. (2016).<br><br>No restrictions by country. English language only.<br><br>Individual studies must include DVA/CM in one of the following ways:	Non peer-reviewed studies<br><br>Surveys or quantitative studies with descriptive free text only<br><br>General literature reviews<br><br>Case reports<br><br>General discussion papers<br><br>Letters<br><br>Commentaries

		o Participation in the study is determined by experience, perpetration or specifically identified as at risk of DVA/CM. Participants may have received an intervention or may be discussing the impact of DVA/CM and their desired outcomes for the future. (To ensure we are not limited by outcomes defined by current interventions).<br>OR<br>o Stakeholders involved in developing and/or delivering interventions to children/families experiencing DVA/CM (c.f. Howarth et al, 2016, p.52), or stakeholder discussion of outcomes that are sought either in relation to an intervention or the future in general.	Editorials<br>Book chapters<br>Conference papers<br>Theses and dissertations
	Population	Any adult or child stakeholders relevant to DVA/CM. This could be because of experience, perpetration, identified as at risk, delivering, commissioning, or intending to deliver services.	
	Phenomenon of interest	DVA/CM	
	Design	Any qualitative approach to data collection and analysis (e.g. interviews, focus groups).	
	Evaluation	Perspectives of experienced or anticipated benefits or harms of interventions, and/or desired outcomes in general related to DVA/CM.	
Review of grey literature	Literature	Any national or regional policy or practice document that reports on DVA/CM-relevant services or outcomes (e.g. measurement/theory).<br>Participation in the service is determined by experience, perpetration or identified as at risk of DVA/CM. (Identification of risk is by practitioners or participants thus we do not have a definition).	Publication in academic journals<br>Book chapters<br>Conference papers<br>Theses and dissertations.

	Published since 2016 to build on Howarth et al. (2016) and Macdonald et al. (2016).<br><br>England-based only. English language only.	
Population	Children or families with children at risk of experiencing, or experiencing DVA/CM. This includes unborn children, children (aged 0 to 18 years), designated as victim or witness.<br><br>For DVA any adult family members who have a caring or parenting role (Early Intervention Foundation, 2014), whether designated as perpetrator, victim, witness, or household member.<br><br>For CM any adult family members who have a caring role, whether designated as perpetrator, witness, or household member.	
Service	Experience of or increased risk of experiencing DVA/CM is a criterion for being offered the service/intervention.<br><br>Services/interventions may be delivered to any family member(s) as an individual or in a group.<br><br>Any duration of service/intervention will be included.<br><br>Any setting will be considered.<br><br>OR<br><br>Any evaluative work or outcomes framework where at least one child or family-level outcome is evaluated/discussed. Family-level outcomes do not need to be explicitly labelled as 'family' level; we will make a judgement. However, they include any outcome that affects the family/household unit. For example, worklessness in study where at least some participants are reported to be parents would be included<br><br>.	Universal services/interventions that do not specifically target children and families at risk of DVA/CM.<br><br>Targeted services/interventions that do not measure any child or family level outcomes e.g. perpetrator programmes that focus solely on attitudinal change.<br><br>DVA (only) services/interventions focused solely on elder abuse, sibling abuse or child perpetration of domestic violence, where participants have not been identified as exposed to DVA (i.e. perpetration of abuse by a child could feasibly be an outcome associated with exposure).

Outcome	Any family or child-level outcome measured or evaluated or discussed in any way.<br><br>Intermediate outcomes that could feasibly represent preconditions needed to reach distal/final outcomes (including those relating to the process of service delivery) will be included, along with final/distal outcomes.

Supplement 4: Rapid evidence review flowcharts

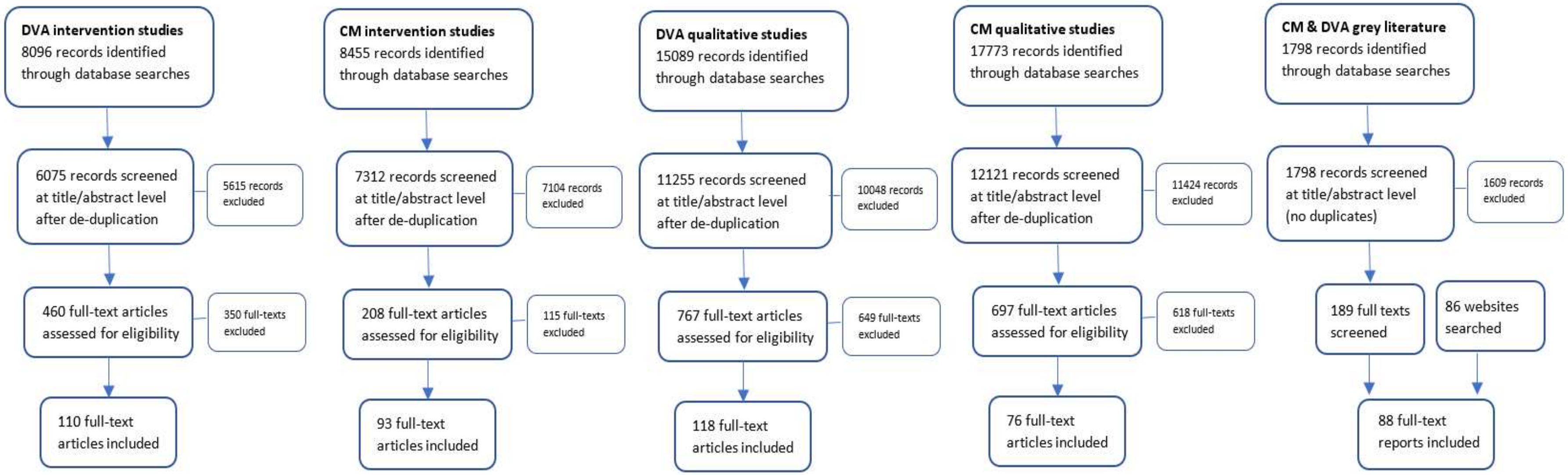

Supplement 5: List of websites searched in grey literature review

Organisation	Website	Date searched
Women's Aid	https://www.womensaid.org.uk/	07/02/20 to 11/02/20
Refuge	www.refuge.org.uk	12/02/2020
Respect	http://respect.uk.net/	12/02/2020
Safe Lives	http://www.safelives.org.uk/	12/02/2020
Voices	http://www.voicescharity.org/	14/02/2020
AVA	https://avaproject.org.uk/	14/02/2020
Standing Together	http://www.standingtogether.org.uk/	14/02/2020
Imkaan	https://www.imkaan.org.uk/	14/02/2020
The Stefanou Foundation	https://www.stefanoufoundation.org/	14/02/2020
Women's Trust	https://womanstrust.org.uk/	14/02/2020
Hestia	https://www.hestia.org/	14/02/2020
DVIP	https://dvip.org/	14/02/2020
Nia	http://www.niaendingviolence.org.uk/	14/02/2020
The Havens	https://www.thehavens.org.uk/	14/02/2020
ManKind Initiative	https://www.mankind.org.uk/	14/02/2020
Everyman Project	http://www.everymanproject.co.uk/	14/02/2020
NCDV (National Centre for Domestic Violence)	https://www.ncdv.org.uk/	14/02/2020
Galop	http://www.galop.org.uk/	14/02/2020
LAWA (Latin American Women's Aid)	http://lawadv.org.uk/en/	14/02/2020
IDAS	https://www.idas.org.uk/	17/02/2020
Advance	http://advancecharity.org.uk/	17/02/2020
Your Sanctuary	https://www.yoursanctuary.org.uk/	17/02/2020
Advocacy After Fatal Domestic Abuse (AAFDA)	https://aafda.org.uk/	17/02/2020
Aurora New Dawn	https://www.aurorand.org.uk/	17/02/2020
My Sister's Place	https://www.mysistersplace.org.uk/	17/02/2020
Surviving Economic Abuse	https://survivingeconomicabuse.org/	17/02/2020
Latin American Women's Rights Service	http://www.lawrs.org.uk/	17/02/2020
Step Up Migrant Women	https://stepupmigrantwomen.org/	17/02/2020
Southall Black Sisters	https://southallblacksisters.org.uk/	17/02/2020
End Violence Against Women	https://www.endviolenceagainstwomen.org.uk/	17/02/2020
Welsh Women's Aid	https://www.welshwomensaid.org.uk/	17/02 2020
Agenda	https://weareagenda.org/	17/02/2020
Rights of women	https://rightsofwomen.org.uk/	17/02/2020
Solace	https://www.solacewomensaid.org/	17/02/2020
Domestic Abuse Housing Alliance	https://www.dahalliance.org.uk/	18/02/2020
Reducing the Risk of Domestic Abuse	https://www.reducingtherisk.org.uk/cms/	18/02/2020
Drive	http://driveproject.org.uk/	18/02/2020
Centre of expertise on child sexual abuse	https://www.csacentre.org.uk/	18/02/2020
FDAC (Family Drug and Alcohol Courts)	https://fdac.org.uk/	18/02/2020
SCIE (Social Care Institute for Excellence)	https://www.scie.org.uk/	18/02/2020
The Survivors Trust	https://www.thesurvivorstrust.org/	19/02/2020
Plan International UK	https://plan-uk.org/	19/02/2020
Victim Support	https://www.victimsupport.org.uk/	19/02/2020
Rape Crisis England & Wales	https://rapecrisis.org.uk/	19/02/2020
IKWRO (Iranian and Kurdish Women's Rights Organisation)	http://ikwro.org.uk/	19/02/2020
Karma Nirvana	https://karmanirvana.org.uk/	19/02/2020
Forward	https://www.forwarduk.org.uk/	19/02/2020
SignHealth	https://www.signhealth.org.uk/	19/02/2020
NSPCC	https://www.nspcc.org.uk/	19/02/2020
NAPAC (The National Association for People Abused in Childhood)	https://napac.org.uk/	19/02/2020
Barnardo's	https://www.barnardos.org.uk/	19/02/2020
Early Intervention Foundation	https://www.eif.org.uk/	19/02/2020
NatCen	http://natcen.ac.uk/	19/02/2020
RCGP (Royal College of General Practitioners)	https://www.rcgp.org.uk/	19/02/2020
RCN (Royal College of Nursing)	https://www.rcn.org.uk/	19/02/2020

Coercive Control	https://coercivecontrol.ripfa.org.uk/	21/02/2020
Domestic Violence UK	https://domesticviolenceuk.org/	21/02/2020
Live Fear Free Helpline	https://gov.wales/live-fear-free	21/02/2020
Living Without Abuse	http://www.lwa.org.uk/	21/02/2020
Hidden Hurt	http://www.hiddenhurt.co.uk/	21/02/2020
RCM (Royal College of Midwives)	https://www.rcm.org.uk/	21/02/2020
NICE (National Institute for Health and Care Excellence)	https://www.nice.org.uk/	21/02/2020
Broken Rainbow UK	https://www.brokenrainbow.org.uk/	21/02/2020
BPS (The British Psychological Society)	https://www.bps.org.uk/	21/02/2020
iHV (Institute of Health Visiting)	https://ihv.org.uk/	21/02/2020
Working together to safeguard children	https://www.gov.uk/government/publications/working-together-to-safeguard-children--2	21/02/2020
WHO	https://www.who.int/	24/02/2020
Public Health England	https://www.gov.uk/government/organisations/public-health-england	24/02/2020
Public Health Scotland	https://publichealthreform.scot/public-health-scotland	24/02/2020
Public Health Wales	https://phw.nhs.wales/	24/02/2020
Public Health Agency Northern Ireland	https://www.publichealth.hscni.net/	26/02/2020
Family Support NI	https://www.familysupportni.gov.uk/	26/02/2020
UNICEF	https://www.unicef.org.uk/	26/02/2020
The National Lottery Community Fund	https://www.tnlcommunityfund.org.uk/	26/02/2020
Comic Relief	https://www.comicrelief.com/	26/02/2020
The Childhood Trust	https://www.childhoodtrust.org.uk/	26/02/2020
College of Policing UK	https://www.college.police.uk/Pages/Home.aspx	26/02/2020
College What Works	https://whatworks.college.police.uk/Pages/default.aspx	26/02/2020
What Works for Children's Social Care	https://whatworks-csc.org.uk/	26/02/2020
Nuffield Foundation	https://www.nuffieldfoundation.org/	27/02/2020
Children's Commissioner for England	https://www.childrenscommissioner.gov.uk/	05/03/2020
Children's Commissioner for Wales	https://www.childcomwales.org.uk/	06/03/2020
Children and Young People's Commissioner Scotland	https://www.cypcs.org.uk/	09/03/2020
Research in Practice	https://www.researchinpractice.org.uk/children/	09/03/2020
The Joseph Rowntree Foundation	https://www.jrf.org.uk/	11/03/2020
British Medical Association (BMA)	https://www.bma.org.uk	18/03/2020

Powell C, *et al. BMJ Open* 2022; 12:e064397. doi: 10.1136/bmjopen-2022-064397

Supplement 6: Overview of e-Delphi survey questions

Round 1: Example question

Q1

Below are the domains in **child health & wellbeing**. These relate to children at any age - at this stage we are not specifying any details, just the broad ideas.

To see further details about each domain hover your mouse/cursor over the item. Then drag and drop into the three categories below, depending on how important it is that they are included in a core outcome set for **child abuse and neglect interventions**. Please try and use all 3 categories.

	Definitely include	Include if possible	Can be dropped
Child physical health			
Child wellbeing			
Child mental distress			
Child sense of self			
Child self-care & coping			
Child social relations & social wellbeing			
Child long term outcomes			

Q1a

Please add any comments on child health & wellbeing in general or any of the domains here:

Round 2: Example question

Q6.1

Below are outcomes in the domain **family function**. Please decide how important you think each outcome is for judging how helpful interventions are for children and families who have experienced child abuse and neglect and rate each one on a nine-point scale before moving on to the next domain.

	9 - Extremely unimportant	8 - Very unimportant	7 - Unimportant	6 - Maybe unimportant	5 - Unsure unimportant or important	4 - Maybe important	3 - Important	2 - Very important	1 - Extremely important
**Family functioning and stability**: includes stable carers	○	○	○	○	○	○	○	○	○
**Family relationships**: includes overall family relationships and functioning, quality and type of relationships, feeling closer as a family, family conflict resolution, feeling closer to children, changes after leaving abusive partner; sibling relationships including after separation; child relationship with birth and foster/adoptive families	○	○	○	○	○	○	○	○	○
**Family emotional environment**: includes emotional security, protective environment, secrecy at home	○	○	○	○	○	○	○	○	○
	9 - Extremely unimportant	8 - Very unimportant	7 - Unimportant	6 - Maybe unimportant	5 - Unsure unimportant or important	4 - Maybe important	3 - Important	2 - Very important	1 - Extremely important
**Family communication**: includes with each other and others outside the family	○	○	○	○	○	○	○	○	○
**Missing episodes** i.e., child missing from home	○	○	○	○	○	○	○	○	○

Q6.2

Please add any comments here about outcomes related to **family function**

Round 3: Example question

Please select up to 10 outcomes (out of 27 in total) that you would like to include in a core outcome set. We recommend that you scroll through the list first and then make your choices.

Please consider that the aim is for this core outcome set to be used across different types of interventions and services. **The outcomes that at least 90% of survivors found important are starred so you can keep the survivor group perspective in mind** because the current focus is mostly on researcher or practitioner priorities. The outcomes are presented in a random order to reduce bias along with the average scores for the survivor group and your individual score so you see how your score compared to other groups. This might inform your decision about what to include. If you missed any questions last time, your previous score will be blank.

As a reminder the scale was:	9 - Extremely unimportant	8 - Very unimportant	7 - Unimportant	6 - Maybe unimportant	5 - Unsure unimportant or important	4 - Maybe important	3 - Important	2 - Very important	1 - Extremely important	0 Selected

☐ **Child stress*** includes signs of stress, i.e., cortisol and other biological measures, subjective feelings of stress

Your previous score = ${e://Field/1A3_r2}
Survivor group average score = 2 - very important

Supplement 7: List of changes to the protocol

- Interviews and consensus workshops were held online to meet national Covid-19 advice and to include participants who were self-isolating.
- We adapted the survey design in response to the longer than expected list of outcomes and to reduce participant burden. In the first round we asked participants to rate outcome domains on a three-point scale, rather than outcomes. The second round then involved a survey of just those outcomes whose domains were selected across the three groups (see methods for details).
- Although the e-Delphi was planned to take place before UK school holidays, Covid-19 delays to the UCL research registration process meant the survey was delayed and took place during the UK summer break. To mitigate against anticipated attrition, new participants were invited to join the survey in the second round.
- Unexpectedly we found extremely high levels of consensus at each stage, thus we increased the consensus levels for outcome inclusion. Staying at the lower levels would have involved including all outcomes. Similarly, we adapted the final round (after participants received feedback) to selecting ten outcomes, rather than re-rating, because all the included outcomes had been rated so highly.

Powell C, *et al. BMJ Open* 2022; 12:e064397. doi: 10.1136/bmjopen-2022-064397

Supplement 8: Rating of e-Delphi survey outcomes

Round 1 scoring: Child maltreatment and domestic violence and abuse

		CM			DVA		
		Survivor	Practitioner	Researcher	Survivor	Practitioner	Researcher
Area	Domain	Median (IQR)	Median (IQR)	Median (IQR)	Median (IQR)	Median (IQR)	Median (IQR)
**Child health & wellbeing**							
	Child physical health	3 (1)	3 (0.5)	3 (0)	3 (0.5)	2 (0.5)	2 (1)
	Child wellbeing	3 (1)	3 (0)	3 (1)	3 (0)	3 (0)	3 (1)
	Child mental distress	3 (0)	3 (0.5)	3 (0)	3 (0)	3 (0.5)	3 (0.5)
	Child sense of self	2 (1.5)	2 (1.5)	2 (1)	3 (1)	2 (0)	2 (1)
	Child self-care & coping	2 (1.5)	2 (0)	2 (1)	2 (1)	2 (0)	2 (1)
	Child social relations & social wellbeing	2 (1.5)	2 (2)	3 (1)	2 (1)	3 (1)	2 (1)
	Child long term outcomes	2 (1.5)	2 (1)	3 (1)	2 (1.5)	2 (0)	2 (1)
**Caregiver health & wellbeing**							
	Caregiver physical health	2 (0.5)	2 (1.5)	2 (1)	3 (1)	2 (0.5)	2 (1)
	Caregiver wellbeing	2 (1)	3 (1)	3 (1)	3 (0.5)	3 (1)	3 (1)
	Caregiver mental distress	3 (1)	3 (1)	3 (0)	3 (0)	3 (0.5)	3 (0.5)
	Caregiver sense of self	1 (1)	2 (1)	2 (1)	2 (1)	2 (0)	2 (1)
	Caregiver self-care & coping	2 (1.25)	2 (0.5)	3 (1)	2 (1)	3 (1)	2 (1)
	Caregiver social relations	2 (2)	2 (1.5)	2 (1)	2 (0.5)	2 (0)	2 (1)
**Caregiver relationships & parenting**							
	Caregiver-child relationship	3 (0)	3 (0)	3 (0)	3 (0)	3 (0)	3 (0)
	Relationship between caregivers	2 (1.5)	2 (1)	2 (1)	2 (1)	2.5 (0.5)	2 (1)
	Parent	3 (1)	2 (1)	3 (0)	2 (1)	3 (1)	3 (1)
	Parental thoughts, feelings, knowledge & understanding of the parental role	2 (0.75)	2 (1)	2 (1)	3 (1)	2 (0)	2 (0.5)
**Home environment & household**							
	Family function	2 (1)	3 (0)	3 (0)	3 (1)	3 (0.5)	3 (0.5)
	Home environment & housing	3 (1)	2 (1)	3 (0.5)	2 (1)	2 (0)	2 (2)
	Family income & employment	2 (2)	2 (1)	2 (1)	2 (1)	2 (1)	2 (1)
**Social support & peer relationships**							
	Child's relationships & social support	3 (0)	3 (0)	3 (1)	3 (1)	3 (1)	3 (1)
	Family relationships & social support	2 (1)	2 (1)	3 (0)	3 (1)	3 (1)	2.5 (1)
**Community resources & institutions**							
	Education/training, delivery & experience (for child/young person)	3 (1)	2 (1)	2 (1)	3 (1)	3 (1)	2 (1)
	Social services delivery & experience	3 (1)	3 (1)	3 (1)	3 (1.5)	3 (0)	3 (1)
	Access, use and knowledge of community resources	2 (1.5)	2 (1)	3 (0.5)	2 (1)	2 (0)	2 (1)
	Experience of community resources	2 (1.75)	2 (1)	2 (1)	2 (0.5)	2 (1)	2 (1)
**Safety, feelings & knowledge related to violence and abuse**							
	Safety	3 (1.5)	3 (1)	3 (0)	3 (0)	3 (0)	3 (0.5)
	Child's contact with harmful people	3 (1.5)	3 (0.5)	3 (0)	3 (0)	3 (1)	2 (1.5)
	Child's thoughts & knowledge about their experiences of CM or DVA	3 (0.5)	3 (1)	3 (1)	3 (1)	3 (0)	2.5 (1)
	Non harming parent's thoughts & feelings about their experience of DVA or maltreatment of their child	2 (0.5)	2 (1)	2 (1)	2 (1)	2 (0)	2 (1)
	Perpetrator/harmful parent perception of responsibility & understanding of violence and abuse	2 (0)	2 (1)	2 (1)	3 (1.5)	2 (0)	2 (0.75)
	Responding after violence & abuse	3 (1)	2 (1)	3 (1)	2 (1)	3 (1)	2 (1)
**Violence, abuse and maltreatment**							
	Child maltreatment	3 (0)	3 (0)	3 (0)	3 (0)	3 (0)	3 (0)
	Child specific exposure to DVA	3 (1)	3 (0.5)	3 (0)	3 (0.5)	3 (0)	3 (0.5)
	Domestic violence and abuse between	2 (1.5)	2 (1)	2 (1)	3 (0)	3 (0.5)	2 (1)
	General violence & abuse	2 (1)	2 (1.5)	2 (1)	2 (0.5)	2 (0)	1.5 (1)
**Intervention outcomes**							
	Process of intervention delivery	2 (0.75)	2 (1)	3 (1)	2 (1)	3 (1)	2 (1)
	Practitioner approach and	2 (1.75)	2 (0.5)	2 (1)	2 (0.5)	2 (0)	2 (1)
	Intervention adverse effects	3 (1)	2 (1)	3 (0)	3 (0.5)	3 (1)	3 (0.5)
	Client experience and evaluation	3 (0.5)	3 (0.5)	3 (1)	2 (1)	3 (1)	3 (1)

Round 2 and 3 scoring: Child maltreatment

Area	Domain	Outcome	Round 2 Survivor Median	(IQR)	Practitioner Median	(IQR)	Researcher Median	(IQR)	Round 3 Survivor Score	(%)	Practitioner Score	(%)	Researcher Score	(%)	TOTAL	RANK
**Child health & wellbeing**																
	Child physical health															
		Child global physical health	3	(2)	2	(2)	2	(2.25)								
		Child sleep	2	(1.5)	3	(2)	2.5	(1.25)								
		Child stress	2	(1)	3	(1.75)	2	(2)	2	(13)	3	(25)	6	(43)	11	14
		Child birth outcomes	3.5	(2)	3	(1)	2	(2)								
		Child pain	2.5	(1)	3	(2)	4	(2)								
		Child physical wellbeing	3	(2)	2.5	(2)	3	(1.25)								
		Child sexual health	3	(2)	2.5	(1)	3	(2)								
	Child wellbeing															
		Child behaviour	2	(2)	2	(2)	1	(1)								
		Child aggression	3	(2)	2	(1)	2	(1)								
		Child development	3	(2)	3	(2)	2	(1.5)								
		Child global functioning	3	(2)	3	(2.5)	2	(2)								
		Child speech and language	3	(1)	3	(2)	3	(1)								
		Child enuresis or soiling related to behaviour	3	(1)	3	(2)	3	(1.5)								
		Child emotional health & wellbeing	2	(1.5)	2	(1)	1	(1)	7	(47)	5	(42)	8	(57)	20	6=
		Child emotional understanding	2	(1)	3	(2)	3	(2)								
		Child ability to concentrate	3	(1.5)	3	(1.5)	3	(2)								
		Child executive functioning	3	(1)	2	(1.75)	3	(1)								
		Child anger	2	(2)	3	(0.5)	4	(1)	3	(20)	1	(8)	3	(21)	7	17
		Child sense of calm	2	(1.5)	3	(1)	2	(1)	1	(7)	0	(0)	0	(0)	1	21
		Child sense of panic	2	(1)	3	(1.5)	3	(2.75)								
		Child happiness	2	(1.5)	3	(1)	2	(1)	3	(20)	1	(8)	2	(14)	6	18
		Child perception of recovery from trauma	2	(0.5)	2	(0.5)	2	(1)	1	(7)	4	(33)	5	(36)	10	15
		Child awareness of own body	3	(2)	3	(2)	2	(1.75)								
		Child overall wellbeing	3	(2)	3	(1.5)	2	(1)								
		Child satisfaction with life	3	(2)	3	(1)	3	(2)								
		Child attitudes towards antisocial behaviour	2	(1.5)	3	(1)	3	(1)								

Child mental distress														
Child overall mental health	2	(2)	1	(1)	1	(1)	9	(60)	6	(50)	8	(57)	23	3
Child anxiety	2	(1)	2	(1)	2	(1)	2	(13)	0	(0)	0	(0)	2	20
Child depression	2	(2.5)	3	(1)	2	(1.5)								
Child dissociation	2	(2)	3	(1.75)	4	(1)								
Child eating disorders	2	(2)	2	(1)	3	(2)								
Child hypervigilance	2	(1)	3	(1.75)	4	(2.5)	3	(20)	1	(8)	1	(7)	5	19
Child irritability	4	(2)	4	(1)	4	(1.75)								
Child post-traumatic stress	2	(1.5)	2	(0.75)	2	(2.75)	5	(33)	4	(33)	5	(36)	14	12=
Child self-harm	2	(2)	2	(1.75)	2	(1)								
Child substance use	3	(1.5)	3	(1)	2	(1)								
Child suicide	1	(1)	1	(0)	1	(1)	8	(53)	5	(42)	5	(36)	18	8=
Child trauma	1	(1)	2	(1)	1	(1)	8	(53)	8	(67)	9	(64)	25	1
**Caregiver health & wellbeing**														
Caregiver mental distress														
Caregiver overall mental health	2	(1.5)	2	(2)	2	(1)								
Caregiver anxiety	3	(1.5)	3	(2)	3	(2)								
Caregiver depression	3	(2)	3	(1)	2	(2)								
Caregiver dissociation	2	(2)	3	(1.75)	4	(1.25)								
Caregiver eating disorders	3	(2)	3	(1)	4	(2)								
Caregiver hypervigilance	2	(1)	3	(1)	4	(1)								
Caregiver irritability	4	(3)	4	(1)	4	(1.25)								
Caregiver post-traumatic stress	2	(1)	2	(1)	3	(2)								
Caregiver self-harm	3	(2.5)	2	(1)	3	(2)								
Caregiver substance use	2	(1)	2	(1)	3	(2)								
Caregiver suicide	2	(1.5)	1	(1)	2	(3)								
Caregiver trauma	2	(2)	2	(1.75)	2.5	(2.25)								
**Caregiver relationships & parenting**														
Caregiver-child relationship														
Parent-child relationship	2	(0)	1	(0.75)	1	(1)	5	(33)	12	(100)	8	(57)	24	2
Parent-child interaction	2	(1.75)	2	(2)	2	(2)								
Parent-child conflict	3	(1)	2	(1)	2	(1)								
Child's feelings about parents	2	(2)	2	(1.75)	3	(3)	7	(47)	7	(58)	3	(21)	17	9=
Relationship with birth family	4	(1)	3.5	(1.75)	3	(1)								
**Home environment & household**														
Family function														

Family functioning and stability	3	(1)	2	(0.75)	2	(1)								
Family relationships	2	(1)	2	(2)	2	(1)	5	(33)	9	(75)	7	(50)	21	5=
Family emotional environment	2	(2)	2	(1)	2	(1)								
Family communication	2	(1.5)	2	(1)	3	(2)								
Missing episodes	4	(1)	4	(1.5)	3	(1)								
**Social support & peer relationships**														
Child's relationships & social support														
Child social support	2	(2)	2	(1.75)	2	(1)	5	(33)	8	(67)	7	(50)	20	6=
Child's relationships with wider family, professionals etc.	3	(1.5)	3	(1.5)	3	(2)								
Child's relationships with peers	2	(1.5)	2	(1)	2	(1)								
Child has trusted relationships	1	(2)	1	(1)	2	(1.25)	6	(40)	7	(58)	8	(57)	21	5=
Connection with other survivors	3	(2)	4	(2)	4	(1)								
Understanding of healthy interpersonal relationships	3	(2)	3	(1)	3	(2.25)								
Ability to seek help	2	(2)	2	(1)	3	(1)								
Family relationships & social support														
Family support	3	(1.5)	2	(2)	2	(1.25)								
Family's relationships with friends	3	(1.5)	3	(2)	4	(1.5)								
Family's relationships with social services	3	(1.5)	4	(3)	3	(1)								
Family feels part of wider community	3	(1)	3	(1)	3.5	(1.25)								
Family social isolation	3	(1)	3	(1)	3	(1.25)								
Fear of social judgement	4	(1)	3	(2)	4	(2)								
**Safety, feelings & knowledge related to violence and abuse**														
Safety														
Caregiver ability to safety plan for self and child	3	(2)	1	(1.75)	2	(2)								
Freedom to go about daily life	2	(1)	2	(1.5)	3	(2)	4	(27)	4	(33)	1	(7)	9	16
Risk of harm	2	(2)	2	(1)	1	(1)	6	(40)	3	(25)	8	(57)	17	9=
Feelings of fear	2	(0.5)	2	(0.5)	3	(3)	6	(40)	2	(17)	4	(29)	12	13
Feelings of safety	2	(1)	2	(1)	2	(2)	7	(47)	8	(67)	4	(29)	19	7
Access to safe spaces	2	(2)	1	(1)	2	(2)								
Child's knowledge and understanding of safety	3	(2)	2	(1.5)	3	(2)								
Child's contact with harmful people														
Child's contact with harmful parent/caregiver	2	(1)	2	(2.75)	1	(2)								
Child's contact with other harmful adults	2	(2)	3	(3)	2	(3)								
**Violence, abuse, and maltreatment**														
Child maltreatment														
Child abuse and neglect	2	(1.5)	1	(1)	1	(1)	8	(53)	5	(42)	9	(64)	22	4

Child physical abuse	1	(1)	1.5	(1)	1	(1.25)	6	(40)	2	(17)	6	(43)	14	12=
Neglect of children by caregivers	2	(2)	1	(1)	1.5	(1)								
Child emotional abuse	1	(1)	1.5	(1.75)	1.5	(2)	8	(53)	2	(17)	5	(36)	15	11=
Child sexual abuse	1	(0.5)	1	(1)	1	(1)	8	(53)	4	(33)	6	(43)	18	8=
Child forced marriage	2	(2)	2	(2)	3	(2.5)								
Child sexual exploitation	1	(2.5)	1.5	(1)	2	(2)								
Child's exposure to DVA	2	(1)	1.5	(2)	2	(3)								
Child experience of witnessing violence and abuse	2.5	(3)	2	(1)	3	(3)								
Risk of re-traumatisation	2	(2)	2	(2)	1	(2.25)								
**Intervention outcomes**														
Intervention adverse effects														
Service harms	2	(1)	2	(1)	2	(2)	6	(40)	6	(50)	4	(29)	16	10
Long term negative impact of intervention	2	(2)	2	(1.5)	2	(1)	4	(27)	4	(33)	7	(50)	15	11=

Powell C, *et al. BMJ Open* 2022; 12:e064397. doi: 10.1136/bmjopen-2022-064397

Round 2 and 3 scoring: Domestic violence and abuse

			Round 2						Round 3							
			Survivor		Practitioner		Researcher		Survivor		Practitioner		Researcher			
Area	Domain	Outcome	Median	(IQR)	Median	(IQR)	Median	(IQR)	Score	(%)	Score	(%)	Score	(%)	TOTAL	RANK
**Child health & wellbeing**																
	Child wellbeing															
		Child behaviour	1	(0)	2	(1)	2	(0.5)	3	(27)	5	(29)	6	(55)	14	9=
		Child aggression	1	(1)	2	(2)	2.5	(1.25)								
		Child development	1	(3)	2	(1.5)	2	(1)								
		Child global functioning	3	(2.5)	2	(1.5)	2	(0.75)								
		Child speech and language	2	(3.5)	2	(1)	3	(1.75)								
		Child enuresis or soiling related to behaviour	2	(3.5)	3	(2)	3.5	(1.75)								
		Child emotional health & wellbeing	1.5	(1.75)	1	(1)	2	(1)								
		Child emotional understanding	1.5	(1.75)	2	(2)	3.5	(2)								
		Child ability to concentrate	2.5	(2.5)	3	(2)	3	(1)								
		Child executive functioning	2	(0.75)	2	(1)	3	(1.75)								
		Child anger	2	(2.5)	2	(2.5)	3.5	(1.75)								
		Child sense of calm	2	(1.75)	2	(2.5)	3	(2)								
		Child sense of panic	1	(1.75)	2	(2)	2.5	(1.75)								
		Child happiness	2	(2.5)	2	(2.5)	3	(2)								
		Child perception of recovery from trauma	1	(0.75)	2	(1)	2	(1.75)	2	(18)	7	(41)	1	(9)	10	13
		Child awareness of own body	1	(1)	1.5	(2)	3	(1.75)								
		Child overall wellbeing	2	(1.5)	2	(2)	3	(2)								
		Child satisfaction with life	1.5	(1.75)	2	(1.75)	3	(2)								
		Child attitudes towards antisocial behaviour	2	(2)	2	(3)	3	(1.75)								
	Child mental distress															
		Child overall mental health	1	(0.5)	1.5	(1)	2	(1)	7	(64)	4	(24)	7	(64)	18	5=
		Child anxiety	2	(1.5)	2.5	(2)	2	(1.5)								
		Child depression	2	(1)	2	(1.75)	2	(2)								
		Child dissociation	1	(2)	2	(1)	3	(1.5)								
		Child eating disorders	2	(2)	3	(1)	3	(1.5)								
		Child hypervigilance	1	(1.5)	2	(1.75)	3	(1.5)								
		Child irritability	3	(3)	3	(3.5)	4	(1.5)								
		Child post-traumatic stress	1	(1)	2	(1.75)	1	(1)	4	(36)	8	(47)	8	(73)	20	4
		Child self-harm	1	(1.5)	3	(2)	2	(2)								
		Child substance use	2	(1.5)	2.5	(1)	3	(1)								
		Child suicide	1	(0.5)	1	(2)	1	(1.5)								

Child trauma	1	(0)	2	(1)	1	(1.5)								
**Caregiver health & wellbeing**														
Caregiver wellbeing														
Caregiver general health & wellbeing	1.5	(1)	2.5	(1)	2	(1.75)	7	(64)	10	(59)	5	(45)	22	3=
Caregiver emotional health & wellbeing	1.5	(1)	2	(1)	2	(2)	3	(27)	8	(47)	4	(36)	15	8=
Caregiver emotional understanding	2	(0.5)	2.5	(1.25)	3	(2)								
Caregiver ability to concentrate	2	(1.25)	3	(2.25)	3	(1)								
Caregiver executive function	3	(1)	2.5	(2.25)	3	(2)								
Caregiver anger	2	(1)	3	(3)	3	(1.5)								
Caregiver sense of calm	2	(2)	2.5	(2.25)	4	(2)								
Caregiver happiness	1	(1.25)	2	(2)	3	(2)								
Caregiver perception of recovery from trauma	2	(2)	2.5	(3)	4	(1.75)								
Caregiver awareness of own body	1	(1.25)	2	(2.25)	3	(1)								
Caregiver satisfaction with life	2	(1.25)	2	(1)	2	(1)								
Caregiver engagement in risky behaviours	2	(1.25)	3	(3)	3	(2)								
Perpetrator health & wellbeing	5	(4)	3	(2.5)	4	(2)								
Caregiver mental distress														
Caregiver overall mental health	1	(0.5)	1	(1)	2	(1)	6	(55)	5	(29)	6	(55)	17	6
Caregiver anxiety	2	(1.5)	3	(1)	3	(1.5)								
Caregiver depression	2	(2)	2	(1)	2	(2)								
Caregiver dissociation	2	(2)	3	(1.5)	3	(1.5)								
Caregiver eating disorders	3	(2)	3	(2)	4	(1)								
Caregiver hypervigilance	2	(1.5)	3	(1.5)	3	(1)								
Caregiver irritability	2	(2.5)	4	(2.75)	4	(0.75)								
Caregiver post-traumatic stress	1	(1)	2	(2)	2	(2)								
Caregiver self-harm	1	(0.5)	3	(2.5)	3	(1.5)								
Caregiver substance use	2	(2)	2	(1.5)	3	(2)								
Caregiver suicide	1	(0)	1	(2)	2	(2)								
Caregiver trauma	1	(0)	1	(1)	2	(2.5)								
**Caregiver relationships & parenting**														
Caregiver-child relationship														
Parent-child relationship	1	(2)	2	(1)	2	(1)	7	(64)	14	(82)	8	(73)	29	2
Parent-child interaction	1	(1.5)	2	(1.5)	2	(1)								
Parent-child conflict	2	(1)	3	(1.5)	3	(1)								
Child's feelings about parents	2	(1)	2	(1.5)	2	(1)								
Relationship with birth family	4	(1)	4	(2.5)	3	(1.5)								
**Home environment & household**														
Family function														
Family functioning and stability	2	(2)	2	(2)	3	(1)	6	(55)	11	(65)	5	(45)	22	3=
Family relationships	2	(1.5)	1.5	(1)	2	(1)	3	(27)	11	(65)	4	(36)	18	5=
Family emotional environment	1	(1)	2	(1.25)	3	(1.75)	4	(36)	8	(47)	3	(27)	15	8=
Family communication	2	(1)	2.5	(3)	3	(1.75)								

Missing episodes	4	(1)	4	(2.75)	3	(1)								
**Safety, feelings & knowledge related to violence and abuse**														
Safety														
Caregiver ability to safety plan for self and child	2	(1.25)	2	(1.5)	2	(2)								
Freedom to go about daily life	1	(0.25)	2	(2)	2	(2)	4	(36)	6	(35)	4	(36)	14	9=
Risk of harm	1	(1)	1	(1)	1	(1)	8	(73)	13	(76)	9	(82)	30	1=
Feelings of fear	1	(1)	2	(1.5)	2	(1.25)	2	(18)	6	(35)	4	(36)	12	11
Feelings of safety	1	(0.5)	1	(1)	1.5	(2)	7	(64)	15	(88)	8	(73)	30	1=
Access to safe spaces	1	(1)	2	(1)	1.5	(1)	5	(45)	4	(24)	4	(36)	13	10
Child's knowledge and understanding of safety	1.5	(1.25)	2	(1)	2	(2)								
Child's thoughts & knowledge about their experiences of CM or DVA														
Child knows DVA is not their fault	1	(0.5)	1	(1)	1	(1)	3	(27)	6	(35)	2	(18)	11	12
Child's ability to make sense of DVA experiences	1	(2)	2	(2)	2.5	(1)								
Child feels validated	2	(2)	1	(2)	2	(2)								
Child's sense that they can move on from violence	1	(1)	1	(1.25)	1.5	(1)	4	(36)	2	(12)	2	(18)	8	15=
Child's ability to identify safe relationships	1	(2)	2	(1.25)	1	(1)								
Child's perception of others' judgements of abuse	2	(1.5)	3	(2.25)	4	(1.75)								
Child's knowledge about DVA	1	(0.5)	1	(1.25)	2	(0.75)	7	(64)	4	(24)	5	(45)	16	7
Child's understanding of impact of violence	2	(2)	2	(2)	3	(1.75)								
Child's understanding of consent	1	(0.5)	2	(1)	2	(2)	4	(36)	2	(12)	3	(27)	9	14
**Violence, abuse, and maltreatment**														
Child maltreatment														
Child abuse and neglect	1	(0)	2	(1)	1	(1.25)	6	(55)	6	(35)	6	(55)	18	5=
Child physical abuse	1	(1)	1.5	(1.25)	1.5	(2)								
Neglect of children by caregivers	1	(0.5)	1.5	(1.25)	1	(1)								
Child emotional abuse	1	(0.5)	1	(1)	2	(2)	2	(18)	4	(24)	2	(18)	8	15=
Child sexual abuse	1	(0)	1	(2.25)	1	(1)								
Child forced marriage	1	(0.5)	2	(2.5)	1	(2)								
Child sexual exploitation	1	(0)	2	(2.25)	1	(1.25)								
Child's exposure to DVA	1	(0)	1	(0)	2	(1.25)								
Child experience of witnessing violence	1	(1)	1.5	(1)	2	(2)	5	(45)	6	(35)	4	(36)	15	8=
Risk of re-traumatisation	1	(0)	2	(1)	2	(3)								
Domestic violence and abuse between caregivers														
Domestic violence and abuse	1	(0)	1	(0)	1	(1)								
Economic abuse	1	(1)	2	(1.5)	2	(2)								
Emotional abuse	1	(0)	1	(2)	1.5	(1.25)								
Physical abuse	1	(1)	2	(2)	1	(1)								
Sexual abuse	1	(0)	2	(2)	1	(1.25)								

Coercive control	1	(0)	1	(1.5)	1	(1)	
Experience of harassment	1	(1)	2	(2)	2	(1)	
Harm to family pets	1	(2)	3	(2.5)	2	(1)	
Injury	1	(0)	2	(2.5)	2	(1)	
Reproductive coercion	1	(1.5)	2	(2)	2	(1.25)	
Perpetrator rehabilitation/desistance	1	(3)	1	(2)	2	(1)	
Perpetration of DVA	1	(1.5)	1	(2)	1.5	(1)	
Risk of re-traumatisation	1	(1)	2	(2)	2	(1.25)	
**Intervention outcomes**							
Intervention adverse effects							
Service harms	1	(0.25)	2	(1.5)	2	(0.25)	
Long term negative impact of intervention	1.5	(2)	2	(3)	2	(1.5)	

Supplement 9: DVA consensus workshop consensus statement

Domestic Violence Core Outcome Set consensus statement

Following the multi-stakeholder workshop on 28th September 2021 the workshop participants agreed to change the outcome *child overall mental health* to *child emotional health and wellbeing* which was part of the original longlist of candidate outcomes. This decision emerged from discussions that emphasised concerns around: i) the medicalisation of young people's distress in response to experiences of DVA; ii) the consensus that emotional health and well-being more readily captures positive functioning and/or adaption rather than emphasising deficits (that some participants felt were implicit in the term *mental health);* iii) the broader applicability of emotional health and wellbeing to sectors outside of health care: the voluntary sector, education and social care. It was agreed that emotional health and wellbeing could encompass specific mental health outcomes such as psychiatric symptoms and diagnoses.

Original outcome

- **Overall mental health** (includes psychiatric symptoms, psychological distress, psychological wellbeing, mental well-being, mental health problems, signs of these including admissions/healthcare or service contacts)

Proposed change:

- **Emotional health & wellbeing** (includes emotions, mood, internalizing problems or symptoms; emotional regulation; emotional security; emotional numbness)

Supplement 10: Consensus workshop outcome rankings

Child maltreatment consensus workshop outcome rankings by number of votes

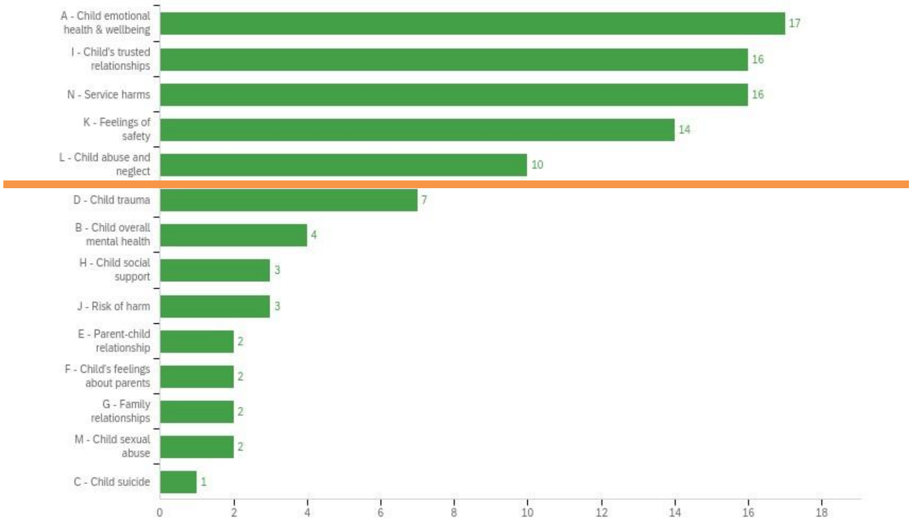

Domestic violence and abuse consensus workshop outcome rankings by number of votes

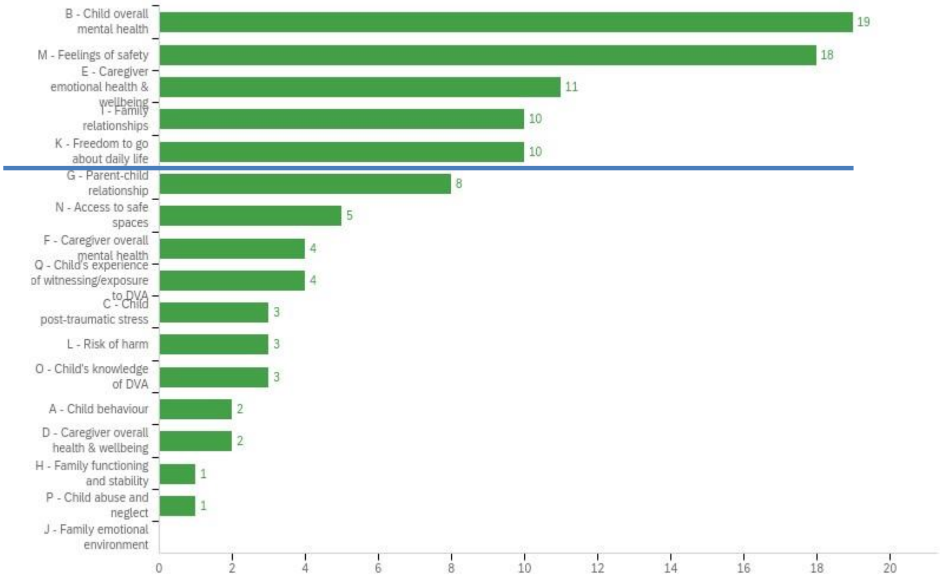

Supplement 11: Summary of discussion about implications of COS for underserved groups

1) Language and meaning

The meaning of concepts related to outcomes was discussed and whether these are understood in the same way by service users and professionals. There was concern about how language could be adapted for all age groups of children without loss of meaning. The cross-cultural understanding of concepts was raised and how accurate translation can be ensured across global settings. Finally, there was discussion about gender in relation to how child behaviour is understood and how violence is analysed.

2) Rights and discrimination

Overall, there was a preference for a rights-based focus as well as understanding of inequalities in any outcome measurement tool. Participants reflected on the impact of socio-economic exclusion and destitution and how particularly minoritised groups, such as migrant children, could be protected. There was a focus on how to centre the child’s voice in any understanding of outcomes. In terms of outcome measurement/assessment there was discussion around how to reduce practitioner bias against marginalised groups, including the misuse of mental health diagnosis.

3) Using measurement tools

Concerns on the practical delivery of any measures used for the core outcome sets, both in terms of the tools themselves and the staff who might be administering the tools.

Powell C, *et al. BMJ Open* 2022; 12:e064397. doi: 10.1136/bmjopen-2022-064397
