# Supplementary material for: Child and family-focused interventions for child maltreatment and domestic abuse: development of core outcome sets
Source: BMJ Open. 2022 Sep 19;12(9):e064397. doi: 10.1136/bmjopen-2022-064397 (PMC9486347; doi:10.1136/bmjopen-2022-064397)
Supplement: Author's manuscript [file bmjopen-2022-064397.draft_revisions.pdf]

BMJ Open

BMJ Open is committed to open peer review. As part of this commitment we make the peer review history of every article we publish publicly available.

When an article is published we post the peer reviewers' comments and the authors' responses online. We also post the versions of the paper that were used during peer review. These are the versions that the peer review comments apply to.

The versions of the paper that follow are the versions that were submitted during the peer review process. They are not the versions of record or the final published versions. They should not be cited or distributed as the published version of this manuscript.

BMJ Open is an open access journal and the full, final, typeset and author-corrected version of record of the manuscript is available on our site with no access controls, subscription charges or pay-per-view fees (http://bmjopen.bmj.com).

If you have any questions on BMJ Open's open peer review process please email info.bmjopen@bmj.com

BMJ Open

Child and family-focused interventions for child maltreatment and domestic abuse: Development of core outcome sets

Journal:	*BMJ Open*
Manuscript ID	bmjopen-2022-064397
Article Type:	Original research
Date Submitted by the Author:	02-May-2022
Complete List of Authors:	Powell, Claire; UCL, Institute of Child Health<br>Feder, Gene; University of Bristol, Community based medicine<br>Gilbert, Ruth; UCL Institute of Child Health, Centre for Paediatric Epidemiology and Biostatistics<br>Paulauskaite, Laura ; UCL, Institute of Education<br>Szilassy, Eszter; University of Bristol, Centre for Academic Primary Care, Population Health Sciences, Bristol Medical School<br>Woodman, Jenny; UCL, Social Research Institute<br>Howarth, Emma; University of East London, School of Psychology
Keywords:	Child protection < PAEDIATRICS, Community child health < PAEDIATRICS, PUBLIC HEALTH, SOCIAL MEDICINE, PRIMARY CARE

SCHOLARONE™
Manuscripts

I, the Submitting Author has the right to grant and does grant on behalf of all authors of the Work (as defined in the below author licence), an exclusive licence and/or a non-exclusive licence for contributions from authors who are: i) UK Crown employees; ii) where BMJ has agreed a CC-BY licence shall apply, and/or iii) in accordance with the terms applicable for US Federal Government officers or employees acting as part of their official duties; on a worldwide, perpetual, irrevocable, royalty-free basis to BMJ Publishing Group Ltd ("BMJ") its licensees and where the relevant Journal is co-owned by BMJ to the co-owners of the Journal, to publish the Work in this journal and any other BMJ products and to exploit all rights, as set out in our licence.

The Submitting Author accepts and understands that any supply made under these terms is made by BMJ to the Submitting Author unless you are acting as an employee on behalf of your employer or a postgraduate student of an affiliated institution which is paying any applicable article publishing charge ("APC") for Open Access articles. Where the Submitting Author wishes to make the Work available on an Open Access basis (and intends to pay the relevant APC), the terms of reuse of such Open Access shall be governed by a Creative Commons licence – details of these licences and which Creative Commons licence will apply to this Work are set out in our licence referred to above.

Other than as permitted in any relevant BMJ Author's Self Archiving Policies, I confirm this Work has not been accepted for publication elsewhere, is not being considered for publication elsewhere and does not duplicate material already published. I confirm all authors consent to publication of this Work and authorise the granting of this licence.

For peer review only

Child and family-focused interventions for child maltreatment and domestic abuse: Development of core outcome sets

Claire Powell* [1], Gene Feder [2], Ruth Gilbert [1], Laura Paulauskaite [3], Eszter Szilassy [2], Jenny Woodman [3], Emma Howarth [4]

Affiliations

[1] Institute of Child Health, UCL, 30 Guilford St, London, UK WC1N 1EH [2] School of Social and Community Medicine, University of Bristol 39 Whatley Road, Bristol, UK BS8 2PS [3] Institute of Education, UCL, 27/8 Woburn Square, London, UK WC1H 0AA [4] School of Psychology, UEL, University Way, London, UK E16 2RD

*Corresponding author

Email: c.powell@ucl.ac.uk

Keywords: domestic abuse, child maltreatment, core outcome sets, interventions, family health

Word count: 3699

For peer review only - http://bmjopen.bmj.com/site/about/guidelines.xhtml

Abstract

Objective

The current evidence for child maltreatment and domestic violence and abuse interventions is limited by the diversity of outcomes evaluated and the variety of measures used. The result is studies that are difficult to compare and lack focus on outcomes reflecting service user or provider priorities. Our aim was to develop core outcome sets (COS) for evaluations of child and family-focused interventions for: i) child maltreatment (CM); and ii) domestic violence and abuse (DVA).

Design

We conducted a two-stage consensus process. Stage one: A longlist of candidate outcomes across CM and DVA was developed through rapid systematic reviews of intervention studies, qualitative and grey literature; stakeholder workshops; survivor interviews. Stage two: Three-panel, three-round e-Delphi surveys for CM and DVA with consensus meetings to agree the final COSs.

Participants

287 stakeholders participated in at least one stage of the process (i.e. either CM or DVA COS development): initial workshops (n=76), two e-Delphi surveys (n=168) and consensus meetings (n=43). Stakeholders included survivors, practitioners, service commissioners, policymakers, and researchers.

Results

Stage one identified 335 outcomes categorised into nine areas and 39 domains. Following stage two, the final five outcomes included in the CM-COS were: child emotional health and wellbeing; child's trusted relationships; feelings of safety; child abuse and neglect; service harms. The final five outcomes in the DVA-COS were: child emotional health and wellbeing; caregiver emotional health and wellbeing; family relationships; freedom to go about daily life; feelings of safety.

Conclusions

We developed two core outcome sets for CM and DVA with two common outcomes (child emotional health and wellbeing; feelings of safety). The COSs reflect shared priorities amongst service users, providers, and researchers. Use of these COSs across trials and service evaluations for children and families affected by CM and DVA will make outcome selection more consistent and help harmonise research and practice.

Article summary

Strengths and Limitations

- This study is a systematic consensus process to develop core outcome sets for interventions for children, parents or families affected by CM and DVA.
- A standardised consensus process revealed the shared priorities between survivors, service providers and researchers.
- Engaging survivors at every stage and reviewing the grey and qualitative literature resulted in core outcomes that are not currently measured in the intervention literature.
- How core outcomes are measured in practice needs further research. Care will be needed to consider the relevance to international settings and minoritised groups.

For peer review only

For peer review only - http://bmjopen.bmj.com/site/about/guidelines.xhtml

Introduction

There is insufficient high-quality evidence for the effectiveness of child maltreatment (CM) and domestic violence and abuse (DVA) interventions for improving child and family outcomes.[1–3] This means service providers do not know which interventions are most useful or might potentially cause harm: this uncertainty could discourage identification of CM or DVA. [4] It is difficult to compare and synthesise studies due to inconsistent outcome reporting and the range of measures used. Even widely reported outcomes such as depression or experience of violence are measured in varied ways,[4,5,6] including across evaluations of similar interventions such as psychological therapies.[7] Current global guidance on evaluation of CM programmes does not promote the use of comparable outcomes. [8]

Decisions about which outcomes to measure tend to be led by researchers, meaning those selected may not be relevant to service users and providers. For example, Howarth et al (2016) reported that clinical trials of interventions for CM or DVA prioritised symptoms and diagnoses, which differed from priorities of affected children and families who emphasised wider outcomes related to everyday wellbeing and functioning.[9] Similarly, work with service users and providers of domestic violence perpetrator programmes found broader definitions of 'success' than are traditionally measured. [10]

Inconsistent or inappropriate outcome reporting and measurement result in research wastage [11] and uncertainty about what interventions work or do not work, and for whom. Consequently, funding might be wasted on ineffective or even harmful interventions.

One way to address these challenges is development of core outcome sets, a minimum set of outcomes which are reported across all clinical trials, and potentially practice-based evaluations as well. COSs should be developed with a standardised consensus process to identify outcomes important to all stakeholders, resulting in the selection of the core outcomes. [10] The aim of a COS is to increase the consistency of outcome measurement and reporting in order that evidence accumulates in helpful ways whilst minimising selective outcome reporting, and ensuring the outcomes are meaningful to service users and providers.

Aim

Our aim was to develop two core outcome sets to be used in evaluations of child, parent, or whole-family interventions for: i) child maltreatment (CM); and ii) domestic violence and abuse (DVA). By developing the two sets in parallel we intended to bring together two areas of practice that may result in a joint service response, [12,13] although they often operate separately.[14] It is acknowledged that collaboration between researchers and practitioners in this area is vital [15] and that the DVA and CM research agendas should be brought together, [16] not least because in up to 60% of homes with DVA, there may also be CM present, [17] leaving children and families with a range of consequences [18,19] and long-term effects. [20]

Scope

The core outcome sets were developed for research and evaluation of any psychosocial interventions (as defined by the Institute of Medicine [21]) for children and families with experience of or at risk of experiencing CM or DVA. We included any intervention globally that aimed to improve child outcomes through targeting parents or family members. The target population was children aged under 19 years with current or previous experience of CM or DVA. Included

For peer review only - http://bmjopen.bmj.com/site/about/guidelines.xhtml

interventions could be delivered in any setting, to an individual or group, and any combination of child, parent/caregiver, family groups alone or in combination. To be in scope, those eligible for the intervention had to have been exposed to CM or DVA or to be at increased risk of CM or DVA.

Methods

We registered the study with the Core Outcome Measures in Effectiveness Trials (COMET) initiative [22] and published the study protocol.[23] Ethics approval was provided by University College London's Research Ethics Committee for involving research participants (17893/001 & 002) and we were guided by a steering group of eight professionals. (For details see acknowledgments.)

Following COMET methodology,[24] we used a two-stage approach. In Stage 1 we devised a longlist of candidate outcomes from stakeholder consultation, qualitative interviews, trials, and the wider literature. In Stage 2, two e-Delphi consensus processes based on this longlist resulted in two core outcome sets. This report of the process and the findings follows COS-STAR guidelines.[25]

See Figure 1 for study flow diagram.

We decided to run Stage 1 jointly for CM and DVA to develop a comprehensive longlist of outcomes that could be relevant to all family-focused solutions and to reflect the high levels of co-occurrence between CM and DVA. However, given differences in current approaches to CM and DVA interventions, for example social care interventions for CM and women's refuge interventions for DVA, we ran Stage 2 as two separate e-Delphi processes to understand differing priorities between these two fields.

Patient and public involvement

We consulted two survivor advisory groups, one comprising adult survivors of domestic violence and abuse, the other comprising young adult care leavers. (For details see acknowledgements). We involved both groups after their participation in the stakeholder workshops and they provided advice on the accessibility and appropriateness of study materials, for example survey wording. They also designed a sensitivity protocol for the qualitative interviews to ensure that these were trauma-informed, consulted on the facilitation of and participated in the consensus workshops. (See online supplement 1 for sensitivity protocol.)

For peer review only

Participants

We recruited from the following groups of participants for the stakeholder workshops (Stage 1), qualitative interviews (Stage 1) and for the e-Delphi survey and consensus workshops (Stage 2):

a. Survivors: UK-based adults with lived experience of CM/DVA in childhood or as the parent of a child who experienced CM/DVA. Survivors did not have to have used any services or interventions to take part, and were recruited through survivor networks, charities and university patient and public involvement (PPI) groups.
b. Practitioners: UK-based professionals working in frontline (i.e. delivering services) or second tier (i.e. supporting service-providers) CM/DVA organisations, local authority commissioning or working in any policy capacity in this area. We recruited through research team networks, directly approaching organisations, and social media.
c. Researchers – English-speaking academic researchers based in high-income country universities or independent research organisations in the UK and internationally. We

For peer review only - http://bmjopen.bmj.com/site/about/guidelines.xhtml

recruited through research networks, directly approaching researchers or teams, and social media.

(See online supplement 2 for Stage 1 and Stage 2 participant details.)

Stage 1: Information sources

In Stage 1 we identified candidate outcomes from three sources: i) rapid evidence reviews, ii) consultation with key stakeholders, and iii) qualitative interviews.

i) Rapid evidence reviews

We carried out a series of rapid reviews separately for CM and DVA, updating previous systematic reviews. [1,2] (See online supplement 3 for inclusion criteria, online supplement 4 for review flow charts and for the search strategies see protocol. [23]) For the intervention literature we searched Medline, Embase, PsycInfo, Cochrane and Web of Science from Jan 2014 to May 2019. For the qualitative literature, ASSIA, CINAHL, GoogleScholar, PsycInfo and SSCI were searched from October 2015 (DVA) and July 2014 (CM) until October 2019. Differences in dates reflect differences in original reviews being updated. The grey literature review was carried out for CM and DVA simultaneously, to identify additional outcomes. We searched websites of relevant organisations (see online supplement 5 for full list) and NICE Evidence Search and Open Grey databases.

Two researchers (CP and EH) dual screened 400 title/abstracts for each of the DVA and CM (200 from the intervention literature searches and 200 from the qualitative literature searches) as a consistency check. The first 10% of full texts were dual screened (CP), with disagreements resolved by discussion as a further consistency check, prior to screening of the rest of the full texts by a single researcher. Study details and outcomes were extracted by a single researcher with 5% cross-checked by the lead researcher. Participant quotations and author-identified themes from the qualitative studies and grey literature were extracted into a matrix to identify outcomes. All outcomes identified from the reviews were added to the longlist and deduplicated as part of the process.

ii) Consultation with key stakeholders

We carried out two half day workshops (19th June 2019 for DVA, 5th September 2019 for CM) in which a total of 76 participants generated a list of candidate outcomes in small groups. The research team and external speakers gave presentations in the workshops on CM and DVA intervention research and interim findings from the rapid evidence reviews. These were followed by small group discussions on definitions of key terms and outcomes. All outcomes from small-group discussions were deduplicated and added to the longlist.

iii) Qualitative interviews

To identify additional outcomes, including those that survivors would like to be measured but are not currently, one researcher (CP) carried out 10 semi-structured interviews in August to December 2020 with survivors of child maltreatment and/or domestic violence and abuse. The interviews were designed in consultation with the study survivor advisory groups and participants gave written, informed consent before each interview, with the option to withdraw at any stage. Interviews lasted 40 - 65 minutes, were audio recorded and transcribed. One interviewee chose to participate by email. Two researchers (CP and LP) extracted outcomes from the interview transcripts, using the questions: i) Is this an outcome? ii) Is this related to an outcome? Outcomes were cross-checked with the longlist and added if they were not already present.

For peer review only - http://bmjopen.bmj.com/site/about/guidelines.xhtml

Stage 1: Longlist and taxonomy development

A single longlist was produced comprising all outcomes gathered from CM and DVA information sources (i.e. the rapid evidence reviews, stakeholder consultations and qualitative interviews). Producing the final longlist involved an iterative process of 1) de-duplicating outcomes produced within each information source (e.g. the consultation workshops); 2) combining outcome lists from information sources to form single longlist; 3) grouping similar outcomes and developing categories to label groups of similar outcomes; 4) deduplicating outcomes across the longlist; 5) combining specific outcome indicators together. Exact duplicates were dropped first, and similar outcomes were reworded to reflect broader meanings. This process was carried out by two researchers (CP and EH) cross-checking and refining the longlist. At several points, the PPI, expert advisory groups, and wider research team provided feedback on specific sections of the list and gave input on the level of detail for the outcomes.

We organised the longlist of outcomes into a taxonomy using an iterative team-based approach. The initial categories, based on participant discussion and notes produced in the stakeholder workshops, were focused on context, and similar to Bronfenbrenner's ecological model in scope. [26] Related theoretical frameworks for CM and DVA were consulted [27–29] and used to further refine the taxonomy, so it accurately reflected the longlist. The taxonomy was finalised by the expert advisory group and the survivor groups reviewed the final categories and outcome wording for sense and missing outcomes. The final longlist and taxonomy were used as the basis for the questionnaires in the e-Delphi survey and final consensus workshop.

See Figure 2 for outcome identification and longlist compilation flowchart

Stage 2: Consensus process

In Stage 2 participants took part in a three-panel three-round e-Delphi survey to reach consensus. The three panels were: survivor, researcher, practitioner (as described above). We used Qualtrics software (Qualtrics, Provo, UT) to collect the data. PPI groups reviewed and edited the survey questions and format for clarity. Survey questions were piloted with one survivor and two team members not involved in its design (LP and RG). Two parallel surveys were conducted, one for CM and one for DVA. Participants gave informed consent as part of the survey, with the option to withdraw their data up to two weeks after completion. (See online supplement 6 for survey.)

See Figure 3 for Stage 2 e-Delphi consensus process and inclusion cut-offs

We asked survivor participants whether they were survivors of CM or DVA and if they had experienced both, they chose whether to participate in the CM or the DVA survey. We assigned professionals to the research or practitioner panels based on information these participants supplied. Although we ran the two surveys in parallel, the first round of the CM and DVA surveys were the same. However, the second round (and subsequently third round and consensus workshop) differed according to the domains and outcomes selected by panels in the CM and DVA surveys. Outcome domains and outcomes were presented in a random order to participants in each round to reduce bias. At the end of each survey we had two shortlists of highly prioritised outcomes, one for CM and one for DVA.

The final stage of the consensus process was online consensus workshops with participants representing the three panels: survivor, practitioner, researcher. Covid-19 restrictions meant the workshops could not be held in person, therefore we invited 24 participants to each workshop (i.e. one for DVA and one for CM), drawing on current recommendations for online consensus

For peer review only - http://bmjopen.bmj.com/site/about/guidelines.xhtml

development meetings from the James Lind Alliance. [30] Information on the purpose of the workshop and the relevant shortlists were sent to all participants ahead of the workshop. Participants discussed their top and bottom three outcomes in two rounds of breakout group sessions, before voting on which to include.

The final core outcome sets include only those outcomes that at least half of workshop participants voted to include. There was a final plenary discussion on the core outcome sets and the implications for marginalised and under-heard groups. To maintain survivor confidentiality, the workshops were not recorded; however, workshop facilitators made detailed notes of discussions. An external facilitator led the workshops to maintain impartiality and members of the research team (CP, EH, ES) acted as neutral co-facilitators of breakout groups. A qualified counsellor was available to speak to survivors during the workshops and for a week afterwards. Further details about how we adapted the process to be trauma-informed will be described in future work.

Results

This study was completed according to the study protocol, any changes were made in response to the evolving COVID-19 situation (e.g. holding interviews and workshops online) and to reduce participant burden (e.g. increasing the survey consensus threshold because the levels of consensus across outcomes was higher than expected). (For full details see online supplement 7).

Stage 1

Six rapid evidence reviews, two stakeholder workshops and ten survivor interviews identified 335 outcomes. (For longlist see https://osf.io/yhnfq/) The outcomes taxonomy comprised 39 domains and nine broad areas which were: 1) child health & wellbeing; 2) caregiver health & wellbeing;3) caregiver relationships and parenting; 4) home environment and household; 5) social support and peer relations; 6) community resources and institutions; 7) safety, feelings and knowledge related to violence and abuse; 8) violence, abuse and maltreatment; 9) intervention outcomes. (For the full taxonomy see https://osf.io/9htz4/)

Stage 2

We recruited a total of 80 participants for the CM e-Delphi survey and 90 participants for the DVA e-Delphi survey. We did not reach our target of 30 participants in all panels; we think this was primarily due to the impact of Covid-19 and associated delays which resulted in the survey running over the school summer holidays.

See Figure 4 for the distribution of participants by round in Stage 2.

Outcomes were dropped at each stage of the e-Delphi. No new outcomes were added throughout the survey process, however suggestions were cross-checked with the longlist and incorporated as details into pre-existing outcomes, for example ‘safety within court proceedings’ was added to the ‘safety’ outcome. Two domains were merged because of feedback from the survey – ‘child specific exposure to DVA’ was incorporated into ‘child maltreatment’.

See Figure 5 for the selection of outcomes

(See online supplement 8 for all ratings of survey items by panel and by round.)

Given ongoing consultation throughout the process regarding practitioner and researcher concerns about the size of the COS, the research team agreed that the core outcome set would be set at five

For peer review only - http://bmjopen.bmj.com/site/about/guidelines.xhtml

outcomes, with discussion around any tied scores. In the event, in both consensus workshops there were five outcomes that scored higher than the rest.

Following discussion in the DVA consensus meeting, it was agreed to change *Child mental health* to *Child emotional health & wellbeing* because participants felt this reflected both survivor and practitioner perspectives in a more holistic way. Workshop participants agreed to a consensus statement to document this change (see online supplement 9).

Core outcome sets

The final core outcome sets, each comprising five outcomes are as follows:

Child Maltreatment COS

1) Child emotional health & wellbeing: includes emotions, mood, internalising problems, emotional regulation, emotional security, and emotional numbness.

2) Child's trusted relationships: includes with friends, family, other adults; network of trust adults – includes access to, quantity and quality, in and out of school.

3) Service harms: includes general harmful service response, iatrogenic harm, replication of abusive dynamics in therapy, re-traumatisation, re-victimisation, secondary abuse, intervention adding to self-blame in women, traumatic physical procedures.

4) Feelings of safety: for non-abusive parent and child; global safety, including psychological, physical, body, family, neighbourhood around perpetrator, at home, at school, in the community, on social media, from abusive individuals, from child removal, from court proceedings.

5) Child abuse and neglect: includes occurrence, recurrence, risk, type.

Domestic Violence and Abuse COS

1) Child emotional health & wellbeing: includes emotions, mood, internalising problems, emotional regulation, emotional security, and emotional numbness.

2) Feelings of safety: for non-abusive parent and child; global safety, including psychological, physical, body, family, neighbourhood around perpetrator, at home, at school, in the community, on social media, from abusive individuals, from child removal, from court proceedings.

3) Caregiver emotional health & wellbeing: includes emotional functioning, emotional reactions, emotions, emotional self-regulation, control over emotions, ability to connect to emotions, mood, frame of mind, general sense of well-being.

4) Family relationships: includes overall family relationships and functioning, quality, and type of relationships, feeling closer as a family, family conflict resolution, feeling closer to children, changes after leaving abusive partner; sibling relationships including after separation; child relationship with birth and foster/adoptive families.

5) Freedom to go about daily life: includes ability to get home safely from school/ work/ friends/ family etc.

See Figure 6 to see the COSs as an infographic.

The outcomes are ordered by number of workshop votes. (For ranking of all discussed outcomes see online supplement 10)

For peer review only - http://bmjopen.bmj.com/site/about/guidelines.xhtml

Core outcome sets and underserved groups

Following agreement on the final sets the afternoon discussion focused on who might be excluded by the core outcome sets. The key groups included: neurodiverse and disabled children and families, families living in poverty, ethnic and racialised minorities. Discussion centred on three topics: 1) language and meaning; 2) rights and discrimination; 3) practical delivery of relevant measurement tools. (For further details see online supplement 11.)

Discussion

We developed two core outcome sets using consensus methods for child and family-focused interventions for CM and DVA. The scope of the outcome sets was broad, including health and wellbeing, safety, and relationship outcomes. The core outcome sets had two outcomes in common - *child emotional health and wellbeing* and *feelings of safety* – reflecting shared priorities between service users and providers across CM and DVA. Furthermore, three of the eight core outcomes are not currently reported in the CM or DVA intervention literature: *child's trusted relationships*, *freedom to go about daily life* or *service harms*. This emphasises the importance of a consensus process involving service users and providers, to capture outcomes critical to all stakeholders, not just researchers.

This is the first time that COS have been developed for CM and DVA, reflecting growing recognition that these experiences co-occur within families and that responses need to consider both CM and DVA. The novelty reflects the fact that research literatures and service provision have largely developed in parallel.

The key strength of our COS development process was the scope of engagement: we involved survivors at every stage both as participants and in an advisory capacity, and we involved professionals from a range of organisations across the non-governmental and statutory sectors. As a result, we identified a broad range of possible outcomes, including those that are not currently measured but nonetheless considered important. The high level of consensus about the importance of many outcomes was unexpected (and resulted in changes to the protocol), highlighting that there are shared priorities across survivors, service providers and researchers. The outcomes identified in this study are consistent with findings from engagement with a range of stakeholders for DVA perpetrator programmes. [10]

Limitations

We faced challenges at every stage because of the Covid-19 pandemic and because we were adapting a process designed for testing medical and surgical interventions, rather than complex psychosocial interventions. Covid-19 restrictions meant we were unable to access as many frontline services as we intended, and it likely affected the survey drop-out rates. Moving online meant we only included survivors who could access the internet, so we did not reach the most marginalised groups of survivors. This limitation needs to be addressed in any future development of CM and DVA core outcome sets.

For these core outcome sets to be used in applied research, the concepts need further refining and linking through an explicit consensus process (with survivors, practitioners and researchers) to appropriate measurement tools. [31] The core outcome sets as they currently stand represent the first stage of harmonising CM and DVA trials and service evaluations, incorporating the survivor perspective. Future work needs to consider the international and cross-cultural relevance of the

For peer review only - http://bmjopen.bmj.com/site/about/guidelines.xhtml

outcomes and their definition, including their application to minoritised groups and families living in varied socioeconomic situations.

Conclusion

Use of COS will enable comparative analyses across multiple intervention studies, including meta-analyses, to understand intervention effectiveness. Furthermore, services will have an evidence base for monitoring outcomes for parent and children service responses to CM and DVA, and to consider the needs of the wider family and the impacts of intervention on carers and children in the short and long term. By developing COS in parallel for CM and IPV, and finding important areas of overlap, such as safety, our findings will help underpin evaluations of family focussed interventions. The shared priorities across the COSs highlight the joint importance of health and wellbeing outcomes rather than a focus on mental health and psychopathology, as well as relationships and safety. The stakeholder involvement and transparent development process have resulted in cross-sector engagement which will be useful for future implementation. Without extensive survivor engagement and reviewing literature beyond clinical studies the core outcome sets would not have reflected all stakeholder priorities.

Acknowledgements

We wish to thank all the survivors who have taken part at every stage of the study; we are grateful to everyone who used their lived experience to improve this work. Further thanks are due to our survivor involvement groups: VOICES (a survivor-led charity for women who have experienced domestic abuse) and Voices from Care (a charity dedicated to the rights of care experienced young people) in partnership with Rachael Vaughan, Cardiff University. We are also grateful to the health and social care professionals and researchers who have participated and the ongoing advice from our steering group: David and Elisabeth Carney Haworth, Hannah Edwards, Victoria Jepson, Elaine Fulton, Dr Deborah Hodes, Professor Sally Kendall and Professor Carol Rivas. Finally, we thank our independent consensus workshop facilitator, Katherine Cowan, and our workshop counsellor, Dr Alison Gregory, for their input.

Legends

Figure 1 – Study design flowchart

Figure 2 – Stage 1: Outcome identification and longlist compilation flowchart

Figure 3 – Stage 2: E-Delphi consensus process to develop and prioritise core outcomes

Figure 4 – Stage 2: Consensus process participant flowchart

Figure 5 – Stage 2: Selection of outcomes flowchart

Figure 6 – Final core outcome sets

Author contributions

EH conceived of the original study design, which was refined and developed by EH, CP, RG, GF, ES, and JW. CP, EH and LP designed data collection tools and collected data for the study. CP cleaned and analysed the data and drafted the paper. EH, RG, GF, ES, JW, CP and LP revised the paper.

For peer review only - http://bmjopen.bmj.com/site/about/guidelines.xhtml

Funding statement

This study is funded by the National Institute for Health Research (NIHR) Policy Research Programme. Funder reference: PR-PRU-1217-21301; UCL award code: 177763. The views expressed are those of the authors and not necessarily those of the NIHR or the Department of Health and Social Care.

Ethics statement

Patient consent for publication not required.

Competing interests

We have read and understood BMJ policy on declaration of interests and declare that we have no competing interests.

Data sharing statement

Please contact the corresponding author or unit manager cpru.data@ucl.ac.uk with enquiries about the data used in this study.

Supplementary material

Online only supplementary material

References

[1] E. Howarth, T.H. Moore, N.J. Welton, N. Lewis, N. Stanley, H. MacMillan, A. Shaw, M. Hester, P. Bryden, G. Feder, IMPRoving Outcomes for children exposed to domestic ViolencE (IMPROVE): an evidence synthesis, Public Health Res. 4 (2016) 1–342. https://doi.org/10.3310/phr04100.

[2] G. Macdonald, N. Livingstone, J. Hanratty, C. McCartan, R. Cotmore, M. Cary, D. Glaser, S. Byford, N.J. Welton, T. Bosqui, L. Bowes, S. Audrey, G. Mezey, H.L. Fisher, W. Riches, R. Churchill, The effectiveness, acceptability and cost-effectiveness of psychosocial interventions for maltreated children and adolescents: an evidence synthesis, Health Technol. Assess. 20 (2016) 1–508. https://doi.org/10.3310/hta20690.

[3] N.E. Latzman, C. Casanueva, J. Brinton, V.L. Forman-Hoffman, The promotion of well-being among children exposed to intimate partner violence: A systematic review of interventions, Campbell Syst. Rev. 15 (2019). http://dx.doi.org/10.1002/cl2.1049.

[4] R. Gilbert, A. Kemp, J. Thoburn, P. Sidebotham, L. Radford, D. Glaser, H.L. MacMillan, Recognising and responding to child maltreatment, The Lancet. 373 (2009) 167–180. https://doi.org/10.1016/S0140-6736(08)61707-9.

[5] S. Jahanfar, L.M. Howard, N. Medley, Interventions for preventing or reducing domestic violence against pregnant women., Cochrane Database Syst. Rev. (2014) CD009414. https://doi.org/10.1002/14651858.CD009414.pub3.

[6] C. Rivas, J. Ramsay, L. Sadowski, L.L. Davidson, D. Dunne, S. Eldridge, K. Hegarty, A. Taft, G. Feder, Advocacy interventions to reduce or eliminate violence and promote the physical and psychosocial well-being of women who experience intimate partner abuse., Cochrane Database Syst. Rev. (2015) CD005043. https://doi.org/10.1002/14651858.CD005043.pub3.

[7] M. Hameed, L. O'Doherty, G. Gilchrist, J. Tirado-Muñoz, A. Taft, P. Chondros, G. Feder, M. Tan, K. Hegarty, Psychological therapies for women who experience intimate partner violence, Cochrane Database Syst. Rev. (2020). https://doi.org/10.1002/14651858.CD013017.pub2.

For peer review only

For peer review only - http://bmjopen.bmj.com/site/about/guidelines.xhtml

[8] L.M. Jones, Improving efforts to prevent children's exposure to violence: A handbook to support the evaluation of child maltreatment prevention programmes., World Health Organization, Geneva Switzerland, 2014. https://apps.who.int/iris/rest/bitstreams/646146/retrieve.
[9] E. Howarth, T.H.M. Moore, A.R.G. Shaw, N.J. Welton, G.S. Feder, M. Hester, H.L. MacMillan, N. Stanley, The Effectiveness of Targeted Interventions for Children Exposed to Domestic Violence: Measuring Success in Ways that Matter to Children, Parents and Professionals, Child Abuse Rev. 24 (2015) 297–310. https://doi.org/10.1002/car.2408.
[10] N. Westmarland, L. Kelly, Why Extending Measurements of "Success" in Domestic Violence Perpetrator Programmes Matters for Social Work, Br. J. Soc. Work. 43 (2013) 1092–1110. https://doi.org/10.1093/bjsw/bcs049.
[11] P. Glasziou, I. Chalmers, Research waste is still a scandal—an essay by Paul Glasziou and Iain Chalmers, BMJ. 363 (2018) k4645. https://doi.org/10.1136/bmj.k4645.
[12] E. Munro, J.S. Taylor, C. Bradbury-Jones, Understanding the Causal Pathways to Child Maltreatment: Implications for Health and Social Care Policy and Practice, Child Abuse Rev. 23 (2014) 61–74. https://doi.org/10.1002/car.2266.
[13] J.L. EDLESON, The Overlap Between Child Maltreatment and Woman Battering, Violence Women. 5 (1999) 134–154. https://doi.org/10.1177/107780129952003.
[14] M. Hester, The Three Planet Model: Towards an Understanding of Contradictions in Approaches to Women and Children's Safety in Contexts of Domestic Violence, Br. J. Soc. Work. 41 (2011) 837–853. https://doi.org/10.1093/bjsw/bcr095.
[15] G. Lessard, P. Alvarez-Lizotte, The exposure of children to intimate partner violence: Potential bridges between two fields in research and psychosocial intervention, Child Abuse Negl. 48 (2015) 29–38. https://doi.org/10.1016/j.chiabu.2015.05.004.
[16] D. Daro, J.L. Edleson, H. Pinderhughes, Finding Common Ground in the Study of Child Maltreatment, Youth Violence, and Adult Domestic Violence, J. Interpers. Violence. 19 (2004) 282–298. https://doi.org/10.1177/0886260503261151.
[17] A.M. Campbell, S.L. Thompson, The emotional maltreatment of children in domestically violent homes: Identifying gaps in education and addressing common misconceptions: The risk of harm to children in domestically violent homes mandates a well-coordinated response, Child Abuse Negl. 48 (2015) 39–49. https://doi.org/10.1016/j.chiabu.2015.08.009.
[18] R. Gilbert, C.S. Widom, K. Browne, D. Fergusson, E. Webb, S. Janson, Burden and consequences of child maltreatment in high-income countries, The Lancet. 373 (2009) 68–81. https://doi.org/10.1016/S0140-6736(08)61706-7.
[19] T.I. Herrenkohl, D.J. Higgins, M.T. Merrick, R.T. Leeb, Positioning a public health framework at the intersection of child maltreatment and intimate partner violence: Primary prevention requires working outside existing systems, Child Abuse Negl. 48 (2015) 22–28. https://doi.org/10.1016/j.chiabu.2015.04.013.
[20] J. Taylor, C. Bradbury-Jones, A. Lazenbatt, F. Soliman, Child maltreatment: pathway to chronic and long-term conditions?, J. Public Health. 38 (2016) 426–431. https://doi.org/10.1093/pubmed/fdv117.
[21] I. of Medicine, Psychosocial Interventions for Mental and Substance Use Disorders: A Framework for Establishing Evidence-Based Standards, The National Academies Press, Washington, DC, 2015. https://www.nap.edu/catalog/19013/psychosocial-interventions-for-mental-and-substance-use-disorders-a-framework (accessed January 31, 2022).
[22] COMET Initiative | Developing Core Outcome Sets for psycho-social interventions to improve outcomes for children with experience, or at risk, of maltreatment or domestic abuse, (n.d.). https://www.cometinitiative.org/Studies/Details/1576 (accessed January 31, 2022).
[23] E. Howarth, C. Powell, J. Woodman, E. Walker, R. Gilbert, G. Feder, Protocol for developing Core Outcome Sets for evaluation of psychosocial interventions for children and families with experience or at risk of child maltreatment or domestic abuse, BMJ Open. (under review).

For peer review only - http://bmjopen.bmj.com/site/about/guidelines.xhtml

[24] J.J. Kirkham, K. Davis, D.G. Altman, J.M. Blazeby, M. Clarke, S. Tunis, P.R. Williamson, Core Outcome Set-STAndards for Development: The COS-STAD recommendations, PLOS Med. 14 (2017) e1002447. https://doi.org/10.1371/journal.pmed.1002447.

[25] J.J. Kirkham, S. Gorst, D.G. Altman, J.M. Blazeby, M. Clarke, D. Devane, E. Gargon, D. Moher, J. Schmitt, P. Tugwell, S. Tunis, P.R. Williamson, Core Outcome Set–STAndards for Reporting: The COS-STAR Statement, PLOS Med. 13 (2016) e1002148. https://doi.org/10.1371/journal.pmed.1002148.

[26] U. Bronfenbrenner, The bioecological model from a life course perspective: Reflections of a participant observer., in: P. Moen, G.H. Elder, K. Lüscher (Eds.), Examining Lives Context Perspect. Ecol. Hum. Dev., American Psychological Association, Washington, 1995: pp. 599–618. https://doi.org/10.1037/10176-017.

[27] C. Firmin, Contextual Safeguarding. An overview of the operational, strategic and conceptual framework., University of Bedfordshire, 2017.

[28] J. Belsky, Etiology of child maltreatment: a developmental-ecological analysis, Psychol. Bull. 114 (1993) 413–434. https://doi.org/10.1037/0033-2909.114.3.413.

[29] L.L. HEISE, Violence Against Women: An Integrated, Ecological Framework, Violence Women. 4 (1998) 262–290. https://doi.org/10.1177/1077801298004003002.

[30] K. Jongsma, J. van Seventer, A. Verwoerd, A. van Rensen, Recommendations from a James Lind Alliance priority setting partnership - a qualitative interview study, Res. Involv. Engagem. 6 (2020) 68. https://doi.org/10.1186/s40900-020-00240-3.

[31] C.A.C. Prinsen, S. Vohra, M.R. Rose, M. Boers, P. Tugwell, M. Clarke, P.R. Williamson, C.B. Terwee, How to select outcome measurement instruments for outcomes included in a "Core Outcome Set" – a practical guideline, Trials. 17 (2016) 449. https://doi.org/10.1186/s13063-016-1555-2.

For peer review only

For peer review only - http://bmjopen.bmj.com/site/about/guidelines.xhtml

Figure 1 – Study design flowchart

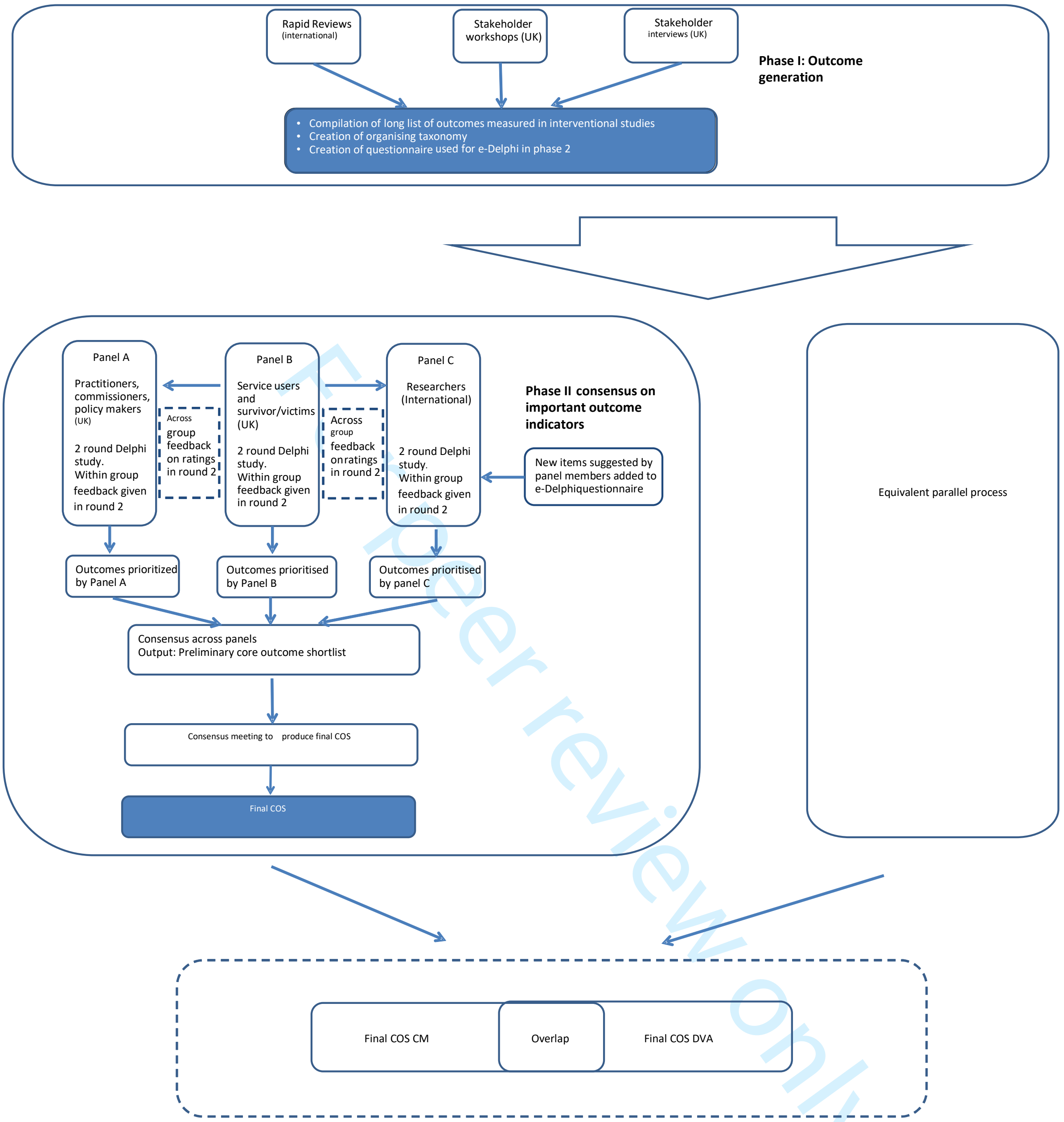

For peer review only

Figure 2 – Stage 1: Outcome identification and longlist compilation flowchart

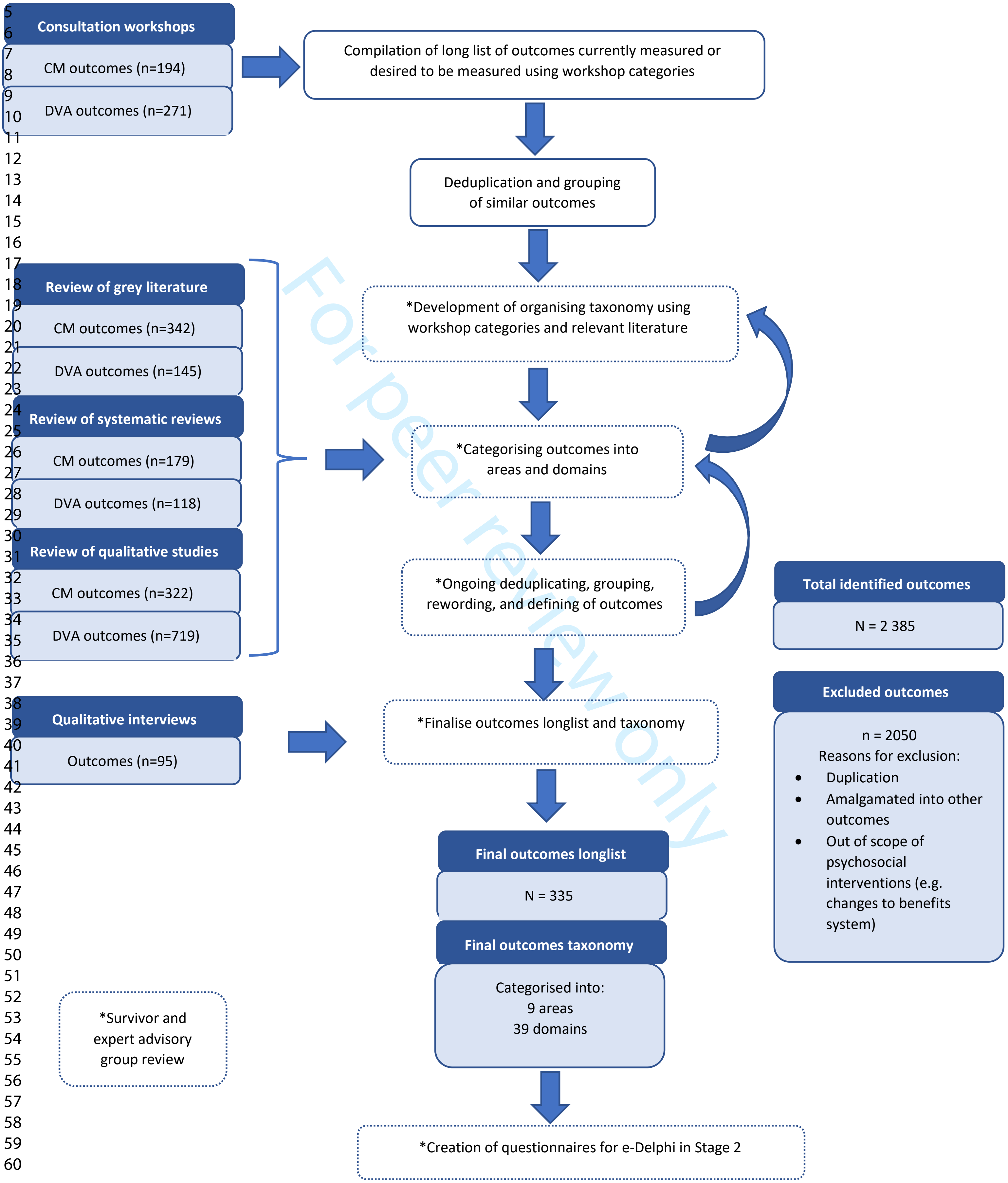

Figure 3 – Stage 2: E-Delphi consensus process to develop and prioritise core outcomes

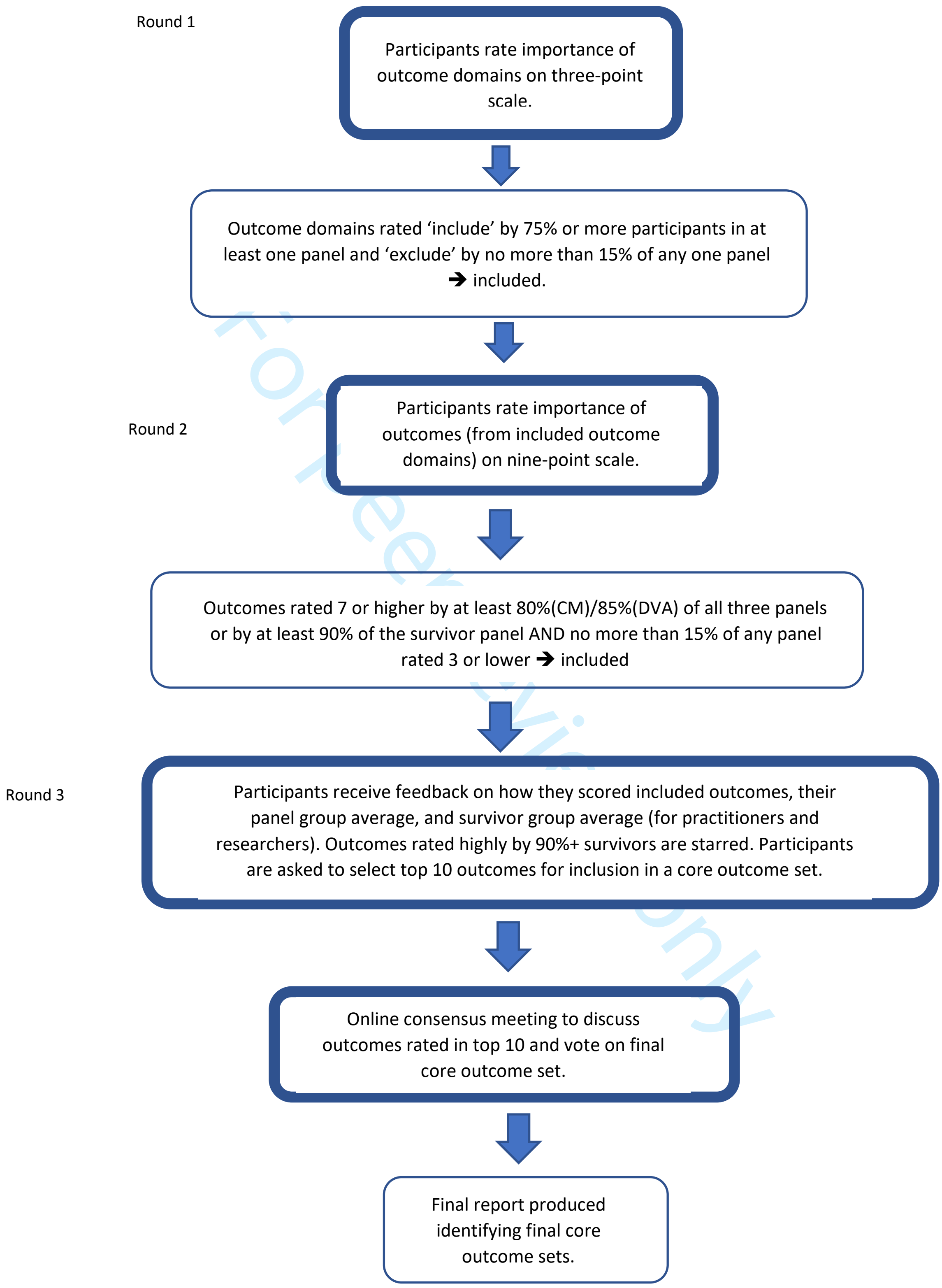

Figure 4 – Stage 2: Consensus process participant flowchart

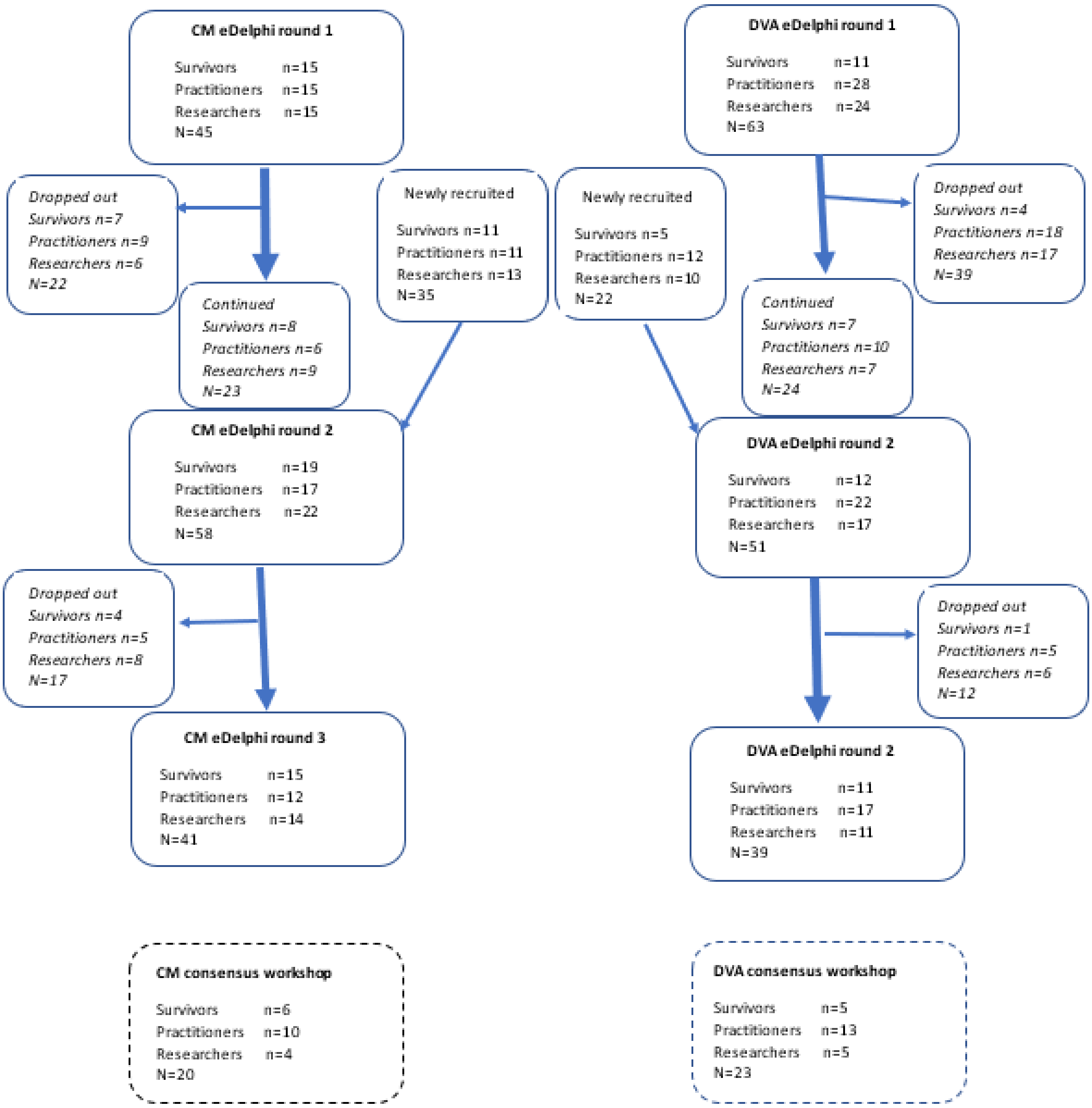

Figure 5 – Stage 2: Selection of outcomes flowchart

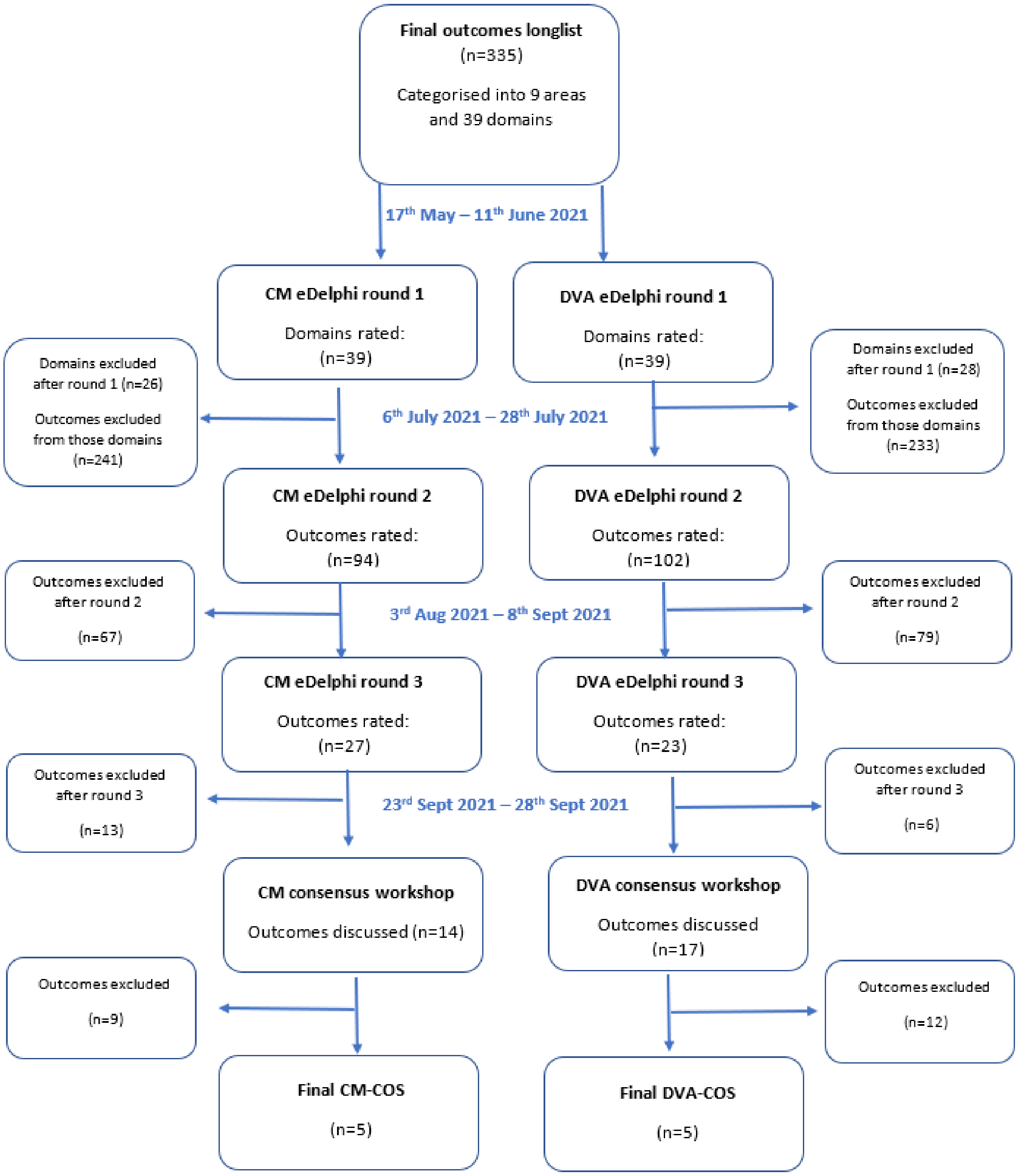

Child maltreatment

- **Child abuse and neglect**
  includes all harms caused to a child by adults in a position of responsibility
- **Service harms**
  Any retraumatisating effects of the inter-vention
- **Child's trusted relationships**
  Positive relationships a child has with any adults
- **Child emotional health & wellbeing**
  All aspects of emotional and mental health
- **Feelings of safety**
  Includes psychological and physical safety
- **Freedom to go about daily life**
  For example, getting home safely from school
- **Family relationships**
  Quality and type of relationship with birth/foster/adoptive family
- **Caregiver emotional health & wellbeing**
  All aspects of emotional and mental health

Domestic violence

Online only supplementary material

Supplement 1: Sensitivity protocol 2

Supplement 2: Characteristics of participants in the identification (Stage 1) and development (Stage 2) of core outcomes for CM and DVA 3

Supplement 3: Table of inclusion criteria for rapid evidence reviews 6

Supplement 4: Rapid evidence review flowcharts 12

Supplement 5: List of websites searched in grey literature review 13

Supplement 6: Overview of e-Delphi survey questions 15

- Round 1: Example question 15
- Round 2: Example question 15
- Round 3: Example question 15

Supplement 7: List of changes to the protocol 16

Supplement 8: Rating of e-Delphi survey outcomes 17

- Round 1 scoring: Child maltreatment and domestic violence and abuse 17
- Round 2 and 3 scoring: Child maltreatment 18
- Round 2 and 3 scoring: Domestic violence and abuse 22

Supplement 9: DVA consensus workshop consensus statement 26

Supplement 10: Consensus workshop outcome rankings 27

Supplement 11: Summary of discussion about implications of COS for underserved groups 28

For peer review only

Supplement 1: Sensitivity protocol

Before the interview

- Ask for permission to let their support organisation know that they're take part
- Offer for interviewee to bring someone to the interview if they want to
- Ask if there's anything that would help make them feel comfortable
- Offer a choice of venue if possible
- Offer possibility that participants can carry interview out by email
- Recap the aim of the interview/research and who is doing the research

During the interview

- Have a box of tissues but not visibly out
- Ask 'are you happy to talk about this?' and skip to the next question if the answer is no
- Allow pauses during the interview – don't rush to fill silences
- Offer break(s) if necessary or to do the interview in two parts
- Allow interviewees chance to go off-topic

After the interview

- Give a list of support options at the end of the interview
- Give option that interviewee can feedback or give more details
- Researcher to send a text message for follow-up and offer a call
- Explain clearly next steps e.g. referrals

For peer review only

For peer review only - http://bmjopen.bmj.com/site/about/guidelines.xhtml

Supplement 2: Characteristics of participants in the identification (Stage 1) and development (Stage 2) of core outcomes for CM and DVA

STAGE ONE

Month 3

Domestic violence and abuse stakeholder workshop held on 19th June 2019 at Friends House, Euston, London		
Survivors	Practitioners	Researchers
10 survivors attended from:<br>• University of Central Lancashire Survivor Researcher Advisory Group<br>• National Survivor User Network (NSUN)<br>• Violence, Abuse and Mental Health Network (VAMHN)	25 practitioners and policymakers attended from:<br>• Frontline and second tier DVA third sector organisations<br>• Local authority service commissioners, government ministries<br>• Schools and education providers	10 researchers and 14 speakers and facilitators attended from:<br>• UK universities and research groups<br>• International universities and research groups

Month 6

Child maltreatment stakeholder workshop held on 5th September 2019 at Institute of Child Health, University College London, London		
Survivors	Practitioners	Researchers
9 survivors attended from:<br>• University of Central Lancashire Survivor Researcher Advisory Group<br>• National Survivor User Network (NSUN)<br>• Violence, Abuse and Mental Health Network (VAMHN)	16 practitioners and policymakers attended from:<br>• Frontline and second tier CM third sector organisations<br>• Local authorities, government ministries<br>• Health service providers and commissioners<br>• Social care professionals and associated organisations	6 researchers and 10 speakers and facilitators attended from:<br>• UK universities and research groups

Months 17 to 21

Qualitative survivor interviews held online from August to December 2020 (N=10)	
Demographics	n (%)
Age	
18 to 35 years	2 (20)
36 to 45 years	2 (20)
46+ years	5 (50)
Unknown	1 (20)
Sex	
Female	8 (80)
Male	2 (20)
Ethnicity	
Asian-European	2 (20)
Black British/Caribbean	2 (20)
White British/UK	5 (50)
Other	1 (10)
Type of abuse	
Child maltreatment only	0 (0)
Domestic violence only	5 (50)
Both	5 (50)

For peer review only

For peer review only - http://bmjopen.bmj.com/site/about/guidelines.xhtml

STAGE TWO

Months 26 to 30

E-Delphi surveys held online from May to Sept 2021 (N=173) *CM and DVA participants amalgamated for anonymity*	
Demographics	n (%)
Panel	
Survivors	42 (25)
Practitioners	66 (39)
Researchers	62 (36)
Age	
18 to 25 years	2 (1)
26 to 35 years	21 (12)
36 to 45 years	49 (29)
46 to 55 years	42(25)
56 to 65 years	44 (26)
66+ years	12 (7)
Sex / gender	
Female	146 (86)
Male	22 (13)
*Non-binary / third gender / Trans**	2 (2)
Ethnicity	
Asian or Asian British (Indian, Pakistani, Bangladeshi, Chinese, Any other Asian background)	10 (6)
Black / African / Caribbean / Black British	6 (4)
Mixed / multiple ethnic groups	16 (9)
White (English, Welsh, Scottish, Northern Irish, British, Irish, Gypsy or Irish Traveller, Any other White background)	137 (81)
Type of abuse (survivors only)	
Child maltreatment only	8 (19)
Domestic violence only	9 (21)
Both	25 (60)

Month 30

Child maltreatment consensus workshop held online on 23rd September 2021		
Survivors	Practitioners	Researchers
6 survivors attended from:<br>• University of Central Lancashire Survivor Researcher Advisory Group<br>• National Survivor User Network (NSUN)<br>• Violence, Abuse and Mental Health Network (VAMHN)<br>• E-Delphi survey participation	*10 practitioners and policymakers attended from:*<br>• Frontline and second tier CM third sector organisations<br>• Local authorities, government ministries<br>• Health service providers and commissioners<br>• Social care professionals and associated organisations	*4 researchers and 4 facilitators attended from:*<br>• UK universities and research groups<br>• International universities and research groups

For peer review only - http://bmjopen.bmj.com/site/about/guidelines.xhtml

Month 30

Domestic violence consensus workshop held online on 28th September 2021		
Survivors	Practitioners	Researchers
5 survivors attended from:<br>• University of Central Lancashire Survivor Researcher Advisory Group<br>• National Survivor User Network (NSUN)<br>• Violence, Abuse and Mental Health Network (VAMHN)<br>• E-Delphi survey participation	*13 practitioners and policymakers attended from:*<br>• Frontline and second tier DVA third sector organisations<br>• Local authorities, government ministries<br>• Health service providers and commissioners<br>• Social care professionals and associated organisation<br>• Police	*5 researchers and 4 facilitators attended from:*<br>• UK universities and research groups

For peer review only

For peer review only - http://bmjopen.bmj.com/site/about/guidelines.xhtml

Supplement 3: Table of inclusion criteria for rapid evidence reviews

Review	Criteria	Inclusion criteria	Exclusion criteria
Review of systematic review	Study type	Peer-reviewed systematic reviews of controlled or quasi experimental comparator intervention studies: with or without randomisation.<br><br>The DARE criteria for SRs are at least 4 of the following: reporting of inclusion/exclusion criteria; adequate search; synthesis of included studies; quality assessment of studies; sufficient detail presented (CRD, 1995). For the purposes of this review, SRs will be included if they use an electronic database and have a structured search strategy.<br><br>Published since 2014.<br><br>No restrictions by country. English language only.<br><br>Individual studies must include DVA/CM in one of the following ways:<br><br>o Entry to the intervention is determined by experience, perpetration or identified as at risk of DVA/CM. (Identification of risk is by researchers, practitioners, or participants thus we do not have a definition)<br><br>o Subgroup analysis is carried out of participants who have experienced (or are considered to be at risk of) DVA/CM<br><br>o DVA/CM is measured as an exposure (this could be retro or prospectively reported)	Non peer-reviewed studies<br><br>Qualitative studies<br><br>General literature reviews<br><br>Protocols<br><br>Case reports<br><br>Cross-sectional studies<br><br>General discussion papers<br><br>Letters<br><br>Commentaries<br><br>Book chapters<br><br>Conference papers<br><br>Theses and dissertations.
	Population	Children or families with children at risk of experiencing or experiencing DVA/CM. This includes unborn children, children (aged 0 to 18 years), designated as victim or witness.<br><br>For DVA any adult family members who have a parenting role (Early Intervention Foundation, 2014), whether designated as perpetrator, victim, witness, or household member.	

For peer review only - http://bmjopen.bmj.com/site/about/guidelines.xhtml

	For CM any adult family members who have a caring role, whether designated as maltreating parent, witness, or household member.<br><br>These adults and children could either be the primary study population of interest or form a subgroup in a wider study population.	
Intervention	Any interventions or services where:<br><br>Experience of or increased risk of experiencing DVA/CM is a criterion for being offered the service<br><br>OR<br><br>DVA/CM is measured as an exposure or outcome of interest<br><br>AND<br><br>At least one child or family-level outcome is measured. Family-level outcomes do not need to be explicitly labelled as 'family' level; we will make a judgement. However, they include any outcome that affects the family/household unit. For example, worklessness in study where at least some participants are reported to be parents would be included.<br><br>Studies must include evaluation of a defined activity/programme and evaluation of a hypothesised effect.<br><br>Interventions may be delivered to any family member(s) as an individual or in a group. Any duration of intervention will be included. Any setting will be considered.	Universal interventions that do not specifically target children and families at risk of<br><br>DVA/CM; targeted interventions that do not measure any child or family level outcomes e.g. perpetrator programmes that focus solely on attitudinal change; DVA (only) interventions focused solely on elder abuse, sibling abuse or child perpetration of domestic violence where participants have not been identified as exposed to DVA.
Comparator	Any control or comparison group/period with participants receiving no care, treatment as usual or any other treatment	

For peer review only

For peer review only - http://bmjopen.bmj.com/site/about/guidelines.xhtml

	Outcome	Any child outcome related to i) the child's experience of adversity ii) child functioning, including risky behaviours.<br><br>Any outcomes related to the quality of the caregiving environment (e.g. parenting, maternal depression, stressful life events, maternal psychological distress, parental substance misuse).<br><br>Any outcomes related to material deprivation e.g. low income, economic hardship, or stress (including perceived), social capital, hunger, food poverty, housing instability.<br><br>Any other outcome judged to relate to children or families by the research team.<br><br>Outcomes can be reported by professionals, child, parent or other family member and they can be retrospective or prospective.<br><br>Outcomes can be end points, surrogate markers for end points or intermediate outcomes.<br><br>No minimum or maximum follow-up is required.	
	Context	Studies from any country in any setting	
Review of qualitative studies	Study type	Primary qualitative (i.e. analysis of interviews, focus groups or other verbal analysis which is not quantified) intervention studies either as a standalone study or a discrete component of mixed method studies.<br><br>Direct and sufficient verbatim text from participants for analysis (i.e. more than two lines) c.f. Arai et al. (2019).<br><br>Published since October 2015 (DVA) and July 2014 (CM) to build on Howarth et al. (2016) and Macdonald et al. (2016).<br><br>No restrictions by country. English language only.<br><br>Individual studies must include DVA/CM in one of the following ways:	Non peer-reviewed studies<br><br>Surveys or quantitative studies with descriptive free text only<br><br>General literature reviews<br><br>Case reports<br><br>General discussion papers<br><br>Letters<br><br>Commentaries

For peer review only - http://bmjopen.bmj.com/site/about/guidelines.xhtml

		o Participation in the study is determined by experience, perpetration or specifically identified as at risk of DVA/CM. Participants may have received an intervention or may be discussing the impact of DVA/CM and their desired outcomes for the future. (To ensure we are not limited by outcomes defined by current interventions).<br><br>OR<br><br>o Stakeholders involved in developing and/or delivering interventions to children/families experiencing DVA/CM (c.f. Howarth et al, 2016, p.52), or stakeholder discussion of outcomes that are sought either in relation to an intervention or the future in general.	Editorials<br><br>Book chapters<br><br>Conference papers<br><br>Theses and dissertations
	Population	Any adult or child stakeholders relevant to DVA/CM. This could be because of experience, perpetration, identified as at risk, delivering, commissioning, or intending to deliver services.	
	Phenomenon of interest	DVA/CM	
	Design	Any qualitative approach to data collection and analysis (e.g. interviews, focus groups).	
	Evaluation	Perspectives of experienced or anticipated benefits or harms of interventions, and/or desired outcomes in general related to DVA/CM.	
Review of grey literature	Literature	Any national or regional policy or practice document that reports on DVA/CM-relevant services or outcomes (e.g. measurement/theory).<br><br>Participation in the service is determined by experience, perpetration or identified as at risk of DVA/CM. (Identification of risk is by practitioners or participants thus we do not have a definition).	Publication in academic journals<br><br>Book chapters<br><br>Conference papers<br><br>Theses and dissertations.

For peer review only - http://bmjopen.bmj.com/site/about/guidelines.xhtml

	Published since 2016 to build on Howarth et al. (2016) and Macdonald et al. (2016).<br><br>England-based only. English language only.	
Population	Children or families with children at risk of experiencing, or experiencing DVA/CM. This includes unborn children, children (aged 0 to 18 years), designated as victim or witness.<br><br>For DVA any adult family members who have a caring or parenting role (Early Intervention Foundation, 2014), whether designated as perpetrator, victim, witness, or household member.<br><br>For CM any adult family members who have a caring role, whether designated as perpetrator, witness, or household member.	
Service	Experience of or increased risk of experiencing DVA/CM is a criterion for being offered the service/intervention.<br><br>Services/interventions may be delivered to any family member(s) as an individual or in a group.<br><br>Any duration of service/intervention will be included.<br><br>Any setting will be considered.<br><br>OR<br><br>Any evaluative work or outcomes framework where at least one child or family-level outcome is evaluated/discussed. Family-level outcomes do not need to be explicitly labelled as 'family' level; we will make a judgement. However, they include any outcome that affects the family/household unit. For example, worklessness in study where at least some participants are reported to be parents would be included<br><br>.	Universal services/interventions that do not specifically target children and families at risk of DVA/CM.<br><br>Targeted services/interventions that do not measure any child or family level outcomes e.g. perpetrator programmes that focus solely on attitudinal change.<br><br>DVA (only) services/interventions focused solely on elder abuse, sibling abuse or child perpetration of domestic violence, where participants have not been identified as exposed to DVA (i.e. perpetration of abuse by a child could feasibly be an outcome associated with exposure).

For peer review only

For peer review only - http://bmjopen.bmj.com/site/about/guidelines.xhtml

Outcome	Any family or child-level outcome measured or evaluated or discussed in any way.<br><br>Intermediate outcomes that could feasibly represent preconditions needed to reach distal/final outcomes (including those relating to the process of service delivery) will be included, along with final/distal outcomes.

For peer review only

For peer review only - http://bmjopen.bmj.com/site/about/guidelines.xhtml

Supplement 4: Rapid evidence review flowcharts

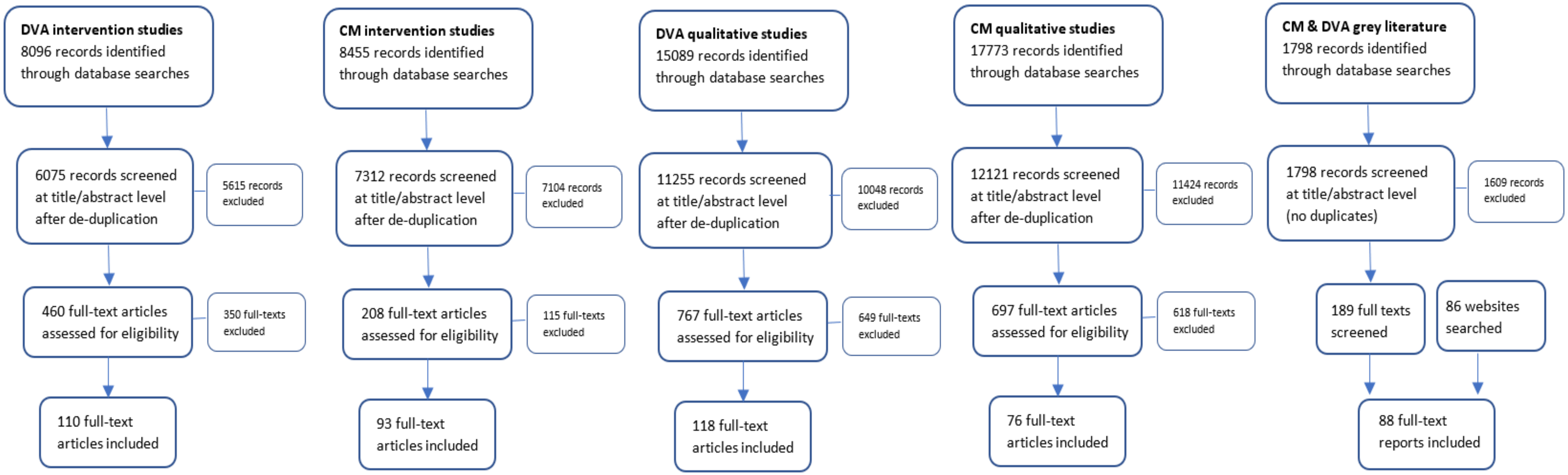

Supplement 5: List of websites searched in grey literature review

Organisation	Website	Date searched
Women's Aid	https://www.womensaid.org.uk/	07/02/20 to 11/02/20
Refuge	www.refuge.org.uk	12/02/2020
Respect	http://respect.uk.net/	12/02/2020
Safe Lives	http://www.safelives.org.uk/	12/02/2020
Voices	http://www.voicescharity.org/	14/02/2020
AVA	https://avaproject.org.uk/	14/02/2020
Standing Together	http://www.standingtogether.org.uk/	14/02/2020
Imkaan	https://www.imkaan.org.uk/	14/02/2020
The Stefanou Foundation	https://www.stefanoufoundation.org/	14/02/2020
Women's Trust	https://womanstrust.org.uk/	14/02/2020
Hestia	https://www.hestia.org/	14/02/2020
DVIP	https://dvip.org/	14/02/2020
Nia	http://www.niaendingviolence.org.uk/	14/02/2020
The Havens	https://www.thehavens.org.uk/	14/02/2020
ManKind Initiative	https://www.mankind.org.uk/	14/02/2020
Everyman Project	http://www.everymanproject.co.uk/	14/02/2020
NCDV (National Centre for Domestic Violence)	https://www.ncdv.org.uk/	14/02/2020
Galop	http://www.galop.org.uk/	14/02/2020
LAWA (Latin American Women's Aid)	http://lawadv.org.uk/en/	14/02/2020
IDAS	https://www.idas.org.uk/	17/02/2020
Advance	http://advancecharity.org.uk/	17/02/2020
Your Sanctuary	https://www.yoursanctuary.org.uk/	17/02/2020
Advocacy After Fatal Domestic Abuse (AAFDA)	https://aafda.org.uk/	17/02/2020
Aurora New Dawn	https://www.aurorand.org.uk/	17/02/2020
My Sister's Place	https://www.mysistersplace.org.uk/	17/02/2020
Surviving Economic Abuse	https://survivingeconomicabuse.org/	17/02/2020
Latin American Women's Rights Service	http://www.lawrs.org.uk/	17/02/2020
Step Up Migrant Women	https://stepupmigrantwomen.org/	17/02/2020
Southall Black Sisters	https://southallblacksisters.org.uk/	17/02/2020
End Violence Against Women	https://www.endviolenceagainstwomen.org.uk/	17/02/2020
Welsh Women's Aid	https://www.welshwomensaid.org.uk/	17/02 2020
Agenda	https://weareagenda.org/	17/02/2020
Rights of women	https://rightsofwomen.org.uk/	17/02/2020
Solace	https://www.solacewomensaid.org/	17/02/2020
Domestic Abuse Housing Alliance	https://www.dahalliance.org.uk/	18/02/2020
Reducing the Risk of Domestic Abuse	https://www.reducingtherisk.org.uk/cms/	18/02/2020
Drive	http://driveproject.org.uk/	18/02/2020
Centre of expertise on child sexual abuse	https://www.csacentre.org.uk/	18/02/2020
FDAC (Family Drug and Alcohol Courts)	https://fdac.org.uk/	18/02/2020
SCIE (Social Care Institute for Excellence)	https://www.scie.org.uk/	18/02/2020
The Survivors Trust	https://www.thesurvivorstrust.org/	19/02/2020
Plan International UK	https://plan-uk.org/	19/02/2020
Victim Support	https://www.victimsupport.org.uk/	19/02/2020
Rape Crisis England & Wales	https://rapecrisis.org.uk/	19/02/2020
IKWRO (Iranian and Kurdish Women's Rights Organisation)	http://ikwro.org.uk/	19/02/2020
Karma Nirvana	https://karmanirvana.org.uk/	19/02/2020
Forward	https://www.forwarduk.org.uk/	19/02/2020
SignHealth	https://www.signhealth.org.uk/	19/02/2020
NSPCC	https://www.nspcc.org.uk/	19/02/2020
NAPAC (The National Association for People Abused in Childhood)	https://napac.org.uk/	19/02/2020
Barnardo's	https://www.barnardos.org.uk/	19/02/2020
Early Intervention Foundation	https://www.eif.org.uk/	19/02/2020
NatCen	http://natcen.ac.uk/	19/02/2020
RCGP (Royal College of General Practitioners)	https://www.rcgp.org.uk/	19/02/2020
RCN (Royal College of Nursing)	https://www.rcn.org.uk/	19/02/2020

For peer review only - http://bmjopen.bmj.com/site/about/guidelines.xhtml

Coercive Control	https://coercivecontrol.ripfa.org.uk/	21/02/2020
Domestic Violence UK	https://domesticviolenceuk.org/	21/02/2020
Live Fear Free Helpline	https://gov.wales/live-fear-free	21/02/2020
Living Without Abuse	http://www.lwa.org.uk/	21/02/2020
Hidden Hurt	http://www.hiddenhurt.co.uk/	21/02/2020
RCM (Royal College of Midwives)	https://www.rcm.org.uk/	21/02/2020
NICE (National Institute for Health and Care Excellence)	https://www.nice.org.uk/	21/02/2020
Broken Rainbow UK	https://www.brokenrainbow.org.uk/	21/02/2020
BPS (The British Psychological Society)	https://www.bps.org.uk/	21/02/2020
iHV (Institute of Health Visiting)	https://ihv.org.uk/	21/02/2020
Working together to safeguard children	https://www.gov.uk/government/publications/working-together-to-safeguard-children--2	21/02/2020
WHO	https://www.who.int/	24/02/2020
Public Health England	https://www.gov.uk/government/organisations/public-health-england	24/02/2020
Public Health Scotland	https://publichealthreform.scot/public-health-scotland	24/02/2020
Public Health Wales	https://phw.nhs.wales/	24/02/2020
Public Health Agency Northern Ireland	https://www.publichealth.hscni.net/	26/02/2020
Family Support NI	https://www.familysupportni.gov.uk/	26/02/2020
UNICEF	https://www.unicef.org.uk/	26/02/2020
The National Lottery Community Fund	https://www.tnlcommunityfund.org.uk/	26/02/2020
Comic Relief	https://www.comicrelief.com/	26/02/2020
The Childhood Trust	https://www.childhoodtrust.org.uk/	26/02/2020
College of Policing UK	https://www.college.police.uk/Pages/Home.aspx	26/02/2020
College What Works	https://whatworks.college.police.uk/Pages/default.aspx	26/02/2020
What Works for Children's Social Care	https://whatworks-csc.org.uk/	26/02/2020
Nuffield Foundation	https://www.nuffieldfoundation.org/	27/02/2020
Children's Commissioner for England	https://www.childrenscommissioner.gov.uk/	05/03/2020
Children's Commissioner for Wales	https://www.childcomwales.org.uk/	06/03/2020
Children and Young People's Commissioner Scotland	https://www.cypcs.org.uk/	09/03/2020
Research in Practice	https://www.researchinpractice.org.uk/children/	09/03/2020
The Joseph Rowntree Foundation	https://www.jrf.org.uk/	11/03/2020
British Medical Association (BMA)	https://www.bma.org.uk	18/03/2020

For peer review only

For peer review only - http://bmjopen.bmj.com/site/about/guidelines.xhtml

Supplement 6: Overview of e-Delphi survey questions

Round 1: Example question

Q1

Below are the domains in **child health & wellbeing**. These relate to children at any age - at this stage we are not specifying any details, just the broad ideas.

To see further details about each domain hover your mouse/cursor over the item. Then drag and drop into the three categories below, depending on how important it is that they are included in a core outcome set for **child abuse and neglect interventions**. Please try and use all 3 categories.

	Definitely include	Include if possible	Can be dropped
Child physical health			
Child wellbeing			
Child mental distress			
Child sense of self			
Child self-care & coping			
Child social relations & social wellbeing			
Child long term outcomes			

Q1a

Please add any comments on child health & wellbeing in general or any of the domains here:

Round 2: Example question

Q6.1

Below are outcomes in the domain **family function**. Please decide how important you think each outcome is for judging how helpful interventions are for children and families who have experienced child abuse and neglect and rate each one on a nine-point scale before moving on to the next domain.

	9 - Extremely unimportant	8 - Very unimportant	7 - Unimportant	6 - Maybe unimportant	5 - Unsure unimportant or important	4 - Maybe important	3 - Important	2 - Very important	1 - Extremely important
**Family functioning and stability**: includes stable carers	○	○	○	○	○	○	○	○	○
**Family relationships**: includes overall family relationships and functioning, quality and type of relationships, feeling closer as a family, family conflict resolution, feeling closer to children, changes after leaving abusive partner; sibling relationships including after separation; child relationship with birth and foster/adoptive families	○	○	○	○	○	○	○	○	○
**Family emotional environment**: includes emotional security, protective environment, secrecy at home	○	○	○	○	○	○	○	○	○
	9 - Extremely unimportant	8 - Very unimportant	7 - Unimportant	6 - Maybe unimportant	5 - Unsure unimportant or important	4 - Maybe important	3 - Important	2 - Very important	1 - Extremely important
**Family communication**: includes with each other and others outside the family	○	○	○	○	○	○	○	○	○
**Missing episodes** i.e., child missing from home	○	○	○	○	○	○	○	○	○

Q6.2

Please add any comments here about outcomes related to **family function**

Round 3: Example question

Please select up to 10 outcomes (out of 27 in total) that you would like to include in a core outcome set. We recommend that you scroll through the list first and then make your choices.

Please consider that the aim is for this core outcome set to be used across different types of interventions and services. **The outcomes that at least 90% of survivors found important are starred so you can keep the survivor group perspective in mind** because the current focus is mostly on researcher or practitioner priorities. The outcomes are presented in a random order to reduce bias along with the average scores for the survivor group and your individual score so you see how your score compared to other groups. This might inform your decision about what to include. If you missed any questions last time, your previous score will be blank.

As a reminder the scale was:	9 - Extremely unimportant	8 - Very unimportant	7 - Unimportant	6 - Maybe unimportant	5 - Unsure unimportant or important	4 - Maybe important	3 - Important	2 - Very important	1 - Extremely important	0 Selected

☐ **Child stress*** includes signs of stress, i.e., cortisol and other biological measures, subjective feelings of stress

Your previous score = ${e://Field/1A3_r2}
Survivor group average score = 2 - very important

Supplement 7: List of changes to the protocol

- Interviews and consensus workshops were held online to meet national Covid-19 advice and to include participants who were self-isolating.
- We adapted the survey design in response to the longer than expected list of outcomes and to reduce participant burden. In the first round we asked participants to rate outcome domains on a three-point scale, rather than outcomes. The second round then involved a survey of just those outcomes whose domains were selected across the three groups (see methods for details).
- Although the e-Delphi was planned to take place before UK school holidays, Covid-19 delays to the UCL research registration process meant the survey was delayed and took place during the UK summer break. To mitigate against anticipated attrition, new participants were invited to join the survey in the second round.
- Unexpectedly we found extremely high levels of consensus at each stage, thus we increased the consensus levels for outcome inclusion. Staying at the lower levels would have involved including all outcomes. Similarly, we adapted the final round (after participants received feedback) to selecting ten outcomes, rather than re-rating, because all the included outcomes had been rated so highly.

For peer review only

For peer review only - http://bmjopen.bmj.com/site/about/guidelines.xhtml

Supplement 8: Rating of e-Delphi survey outcomes

Round 1 scoring: Child maltreatment and domestic violence and abuse

Area	Domain	CM Survivor Median (IQR)	CM Practitioner Median (IQR)	CM Researcher Median (IQR)	DVA Survivor Median (IQR)	DVA Practitioner Median (IQR)	DVA Researcher Median (IQR)
**Child health & wellbeing**							
	Child physical health	3 (1)	3 (0.5)	3 (0)	3 (0.5)	2 (0.5)	2 (1)
	Child wellbeing	3 (1)	3 (0)	3 (1)	3 (0)	3 (0)	3 (1)
	Child mental distress	3 (0)	3 (0.5)	3 (0)	3 (0)	3 (0.5)	3 (0.5)
	Child sense of self	2 (1.5)	2 (1.5)	2 (1)	3 (1)	2 (0)	2 (1)
	Child self-care & coping	2 (1.5)	2 (0)	2 (1)	2 (1)	2 (0)	2 (1)
	Child social relations & social wellbeing	2 (1.5)	2 (2)	3 (1)	2 (1)	3 (1)	2 (1)
	Child long term outcomes	2 (1.5)	2 (1)	3 (1)	2 (1.5)	2 (0)	2 (1)
**Caregiver health & wellbeing**							
	Caregiver physical health	2 (0.5)	2 (1.5)	2 (1)	3 (1)	2 (0.5)	2 (1)
	Caregiver wellbeing	2 (1)	3 (1)	3 (1)	3 (0.5)	3 (1)	3 (1)
	Caregiver mental distress	3 (1)	3 (1)	3 (0)	3 (0)	3 (0.5)	3 (0.5)
	Caregiver sense of self	1 (1)	2 (1)	2 (1)	2 (1)	2 (0)	2 (1)
	Caregiver self-care & coping	2 (1.25)	2 (0.5)	3 (1)	2 (1)	3 (1)	2 (1)
	Caregiver social relations	2 (2)	2 (1.5)	2 (1)	2 (0.5)	2 (0)	2 (1)
**Caregiver relationships & parenting**							
	Caregiver-child relationship	3 (0)	3 (0)	3 (0)	3 (0)	3 (0)	3 (0)
	Relationship between caregivers	2 (1.5)	2 (1)	2 (1)	2 (1)	2.5 (0.5)	2 (1)
	Parent	3 (1)	2 (1)	3 (0)	2 (1)	3 (1)	3 (1)
	Parental thoughts, feelings, knowledge & understanding of the parental role	2 (0.75)	2 (1)	2 (1)	3 (1)	2 (0)	2 (0.5)
**Home environment & household**							
	Family function	2 (1)	3 (0)	3 (0)	3 (1)	3 (0.5)	3 (0.5)
	Home environment & housing	3 (1)	2 (1)	3 (0.5)	2 (1)	2 (0)	2 (2)
	Family income & employment	2 (2)	2 (1)	2 (1)	2 (1)	2 (1)	2 (1)
**Social support & peer relationships**							
	Child's relationships & social support	3 (0)	3 (0)	3 (1)	3 (1)	3 (1)	3 (1)
	Family relationships & social support	2 (1)	2 (1)	3 (0)	3 (1)	3 (1)	2.5 (1)
**Community resources & institutions**							
	Education/training, delivery & experience (for child/young person)	3 (1)	2 (1)	2 (1)	3 (1)	3 (1)	2 (1)
	Social services delivery & experience	3 (1)	3 (1)	3 (1)	3 (1.5)	3 (0)	3 (1)
	Access, use and knowledge of community resources	2 (1.5)	2 (1)	3 (0.5)	2 (1)	2 (0)	2 (1)
	Experience of community resources	2 (1.75)	2 (1)	2 (1)	2 (0.5)	2 (1)	2 (1)
**Safety, feelings & knowledge related to violence and abuse**							
	Safety	3 (1.5)	3 (1)	3 (0)	3 (0)	3 (0)	3 (0.5)
	Child's contact with harmful people	3 (1.5)	3 (0.5)	3 (0)	3 (0)	3 (1)	2 (1.5)
	Child's thoughts & knowledge about their experiences of CM or DVA	3 (0.5)	3 (1)	3 (1)	3 (1)	3 (0)	2.5 (1)
	Non harming parent's thoughts & feelings about their experience of DVA or maltreatment of their child	2 (0.5)	2 (1)	2 (1)	2 (1)	2 (0)	2 (1)
	Perpetrator/harmful parent perception of responsibility & understanding of violence and abuse	2 (0)	2 (1)	2 (1)	3 (1.5)	2 (0)	2 (0.75)
	Responding after violence & abuse	3 (1)	2 (1)	3 (1)	2 (1)	3 (1)	2 (1)
**Violence, abuse and maltreatment**							
	Child maltreatment	3 (0)	3 (0)	3 (0)	3 (0)	3 (0)	3 (0)
	Child specific exposure to DVA	3 (1)	3 (0.5)	3 (0)	3 (0.5)	3 (0)	3 (0.5)
	Domestic violence and abuse between	2 (1.5)	2 (1)	2 (1)	3 (0)	3 (0.5)	2 (1)
	General violence & abuse	2 (1)	2 (1.5)	2 (1)	2 (0.5)	2 (0)	1.5 (1)
**Intervention outcomes**							
	Process of intervention delivery	2 (0.75)	2 (1)	3 (1)	2 (1)	3 (1)	2 (1)
	Practitioner approach and	2 (1.75)	2 (0.5)	2 (1)	2 (0.5)	2 (0)	2 (1)
	Intervention adverse effects	3 (1)	2 (1)	3 (0)	3 (0.5)	3 (1)	3 (0.5)
	Client experience and evaluation	3 (0.5)	3 (0.5)	3 (1)	2 (1)	3 (1)	3 (1)

For peer review only - http://bmjopen.bmj.com/site/about/guidelines.xhtml

Round 2 and 3 scoring: Child maltreatment

			Round 2						Round 3							
			Survivor		Practitioner		Researcher		Survivor		Practitioner		Researcher			
Area	Domain	Outcome	Median	(IQR)	Median	(IQR)	Median	(IQR)	Score	(%)	Score	(%)	Score	(%)	TOTAL	RANK
**Child health & wellbeing**																
	Child physical health															
		Child global physical health	3	(2)	2	(2)	2	(2.25)								
		Child sleep	2	(1.5)	3	(2)	2.5	(1.25)								
		Child stress	2	(1)	3	(1.75)	2	(2)	2	(13)	3	(25)	6	(43)	11	14
		Child birth outcomes	3.5	(2)	3	(1)	2	(2)								
		Child pain	2.5	(1)	3	(2)	4	(2)								
		Child physical wellbeing	3	(2)	2.5	(2)	3	(1.25)								
		Child sexual health	3	(2)	2.5	(1)	3	(2)								
	Child wellbeing															
		Child behaviour	2	(2)	2	(2)	1	(1)								
		Child aggression	3	(2)	2	(1)	2	(1)								
		Child development	3	(2)	3	(2)	2	(1.5)								
		Child global functioning	3	(2)	3	(2.5)	2	(2)								
		Child speech and language	3	(1)	3	(2)	3	(1)								
		Child enuresis or soiling related to behaviour	3	(1)	3	(2)	3	(1.5)								
		Child emotional health & wellbeing	2	(1.5)	2	(1)	1	(1)	7	(47)	5	(42)	8	(57)	20	6=
		Child emotional understanding	2	(1)	3	(2)	3	(2)								
		Child ability to concentrate	3	(1.5)	3	(1.5)	3	(2)								
		Child executive functioning	3	(1)	2	(1.75)	3	(1)								
		Child anger	2	(2)	3	(0.5)	4	(1)	3	(20)	1	(8)	3	(21)	7	17
		Child sense of calm	2	(1.5)	3	(1)	2	(1)	1	(7)	0	(0)	0	(0)	1	21
		Child sense of panic	2	(1)	3	(1.5)	3	(2.75)								
		Child happiness	2	(1.5)	3	(1)	2	(1)	3	(20)	1	(8)	2	(14)	6	18
		Child perception of recovery from trauma	2	(0.5)	2	(0.5)	2	(1)	1	(7)	4	(33)	5	(36)	10	15
		Child awareness of own body	3	(2)	3	(2)	2	(1.75)								
		Child overall wellbeing	3	(2)	3	(1.5)	2	(1)								
		Child satisfaction with life	3	(2)	3	(1)	3	(2)								
		Child attitudes towards antisocial behaviour	2	(1.5)	3	(1)	3	(1)								

For peer review only - http://bmjopen.bmj.com/site/about/guidelines.xhtml

Child mental distress														
Child overall mental health	2	(2)	1	(1)	1	(1)	9	(60)	6	(50)	8	(57)	23	3
Child anxiety	2	(1)	2	(1)	2	(1)	2	(13)	0	(0)	0	(0)	2	20
Child depression	2	(2.5)	3	(1)	2	(1.5)								
Child dissociation	2	(2)	3	(1.75)	4	(1)								
Child eating disorders	2	(2)	2	(1)	3	(2)								
Child hypervigilance	2	(1)	3	(1.75)	4	(2.5)	3	(20)	1	(8)	1	(7)	5	19
Child irritability	4	(2)	4	(1)	4	(1.75)								
Child post-traumatic stress	2	(1.5)	2	(0.75)	2	(2.75)	5	(33)	4	(33)	5	(36)	14	12=
Child self-harm	2	(2)	2	(1.75)	2	(1)								
Child substance use	3	(1.5)	3	(1)	2	(1)								
Child suicide	1	(1)	1	(0)	1	(1)	8	(53)	5	(42)	5	(36)	18	8=
Child trauma	1	(1)	2	(1)	1	(1)	8	(53)	8	(67)	9	(64)	25	1
**Caregiver health & wellbeing**														
Caregiver mental distress														
Caregiver overall mental health	2	(1.5)	2	(2)	2	(1)								
Caregiver anxiety	3	(1.5)	3	(2)	3	(2)								
Caregiver depression	3	(2)	3	(1)	2	(2)								
Caregiver dissociation	2	(2)	3	(1.75)	4	(1.25)								
Caregiver eating disorders	3	(2)	3	(1)	4	(2)								
Caregiver hypervigilance	2	(1)	3	(1)	4	(1)								
Caregiver irritability	4	(3)	4	(1)	4	(1.25)								
Caregiver post-traumatic stress	2	(1)	2	(1)	3	(2)								
Caregiver self-harm	3	(2.5)	2	(1)	3	(2)								
Caregiver substance use	2	(1)	2	(1)	3	(2)								
Caregiver suicide	2	(1.5)	1	(1)	2	(3)								
Caregiver trauma	2	(2)	2	(1.75)	2.5	(2.25)								
**Caregiver relationships & parenting**														
Caregiver-child relationship														
Parent-child relationship	2	(0)	1	(0.75)	1	(1)	5	(33)	12	(100)	8	(57)	24	2
Parent-child interaction	2	(1.75)	2	(2)	2	(2)								
Parent-child conflict	3	(1)	2	(1)	2	(1)								
Child's feelings about parents	2	(2)	2	(1.75)	3	(3)	7	(47)	7	(58)	3	(21)	17	9=
Relationship with birth family	4	(1)	3.5	(1.75)	3	(1)								
**Home environment & household**														
Family function														

Family functioning and stability	3	(1)	2	(0.75)	2	(1)								
Family relationships	2	(1)	2	(2)	2	(1)	5	(33)	9	(75)	7	(50)	21	5=
Family emotional environment	2	(2)	2	(1)	2	(1)								
Family communication	2	(1.5)	2	(1)	3	(2)								
Missing episodes	4	(1)	4	(1.5)	3	(1)								
**Social support & peer relationships**														
Child's relationships & social support														
Child social support	2	(2)	2	(1.75)	2	(1)	5	(33)	8	(67)	7	(50)	20	6=
Child's relationships with wider family, professionals etc.	3	(1.5)	3	(1.5)	3	(2)								
Child's relationships with peers	2	(1.5)	2	(1)	2	(1)								
Child has trusted relationships	1	(2)	1	(1)	2	(1.25)	6	(40)	7	(58)	8	(57)	21	5=
Connection with other survivors	3	(2)	4	(2)	4	(1)								
Understanding of healthy interpersonal relationships	3	(2)	3	(1)	3	(2.25)								
Ability to seek help	2	(2)	2	(1)	3	(1)								
Family relationships & social support														
Family support	3	(1.5)	2	(2)	2	(1.25)								
Family's relationships with friends	3	(1.5)	3	(2)	4	(1.5)								
Family's relationships with social services	3	(1.5)	4	(3)	3	(1)								
Family feels part of wider community	3	(1)	3	(1)	3.5	(1.25)								
Family social isolation	3	(1)	3	(1)	3	(1.25)								
Fear of social judgement	4	(1)	3	(2)	4	(2)								
**Safety, feelings & knowledge related to violence and abuse**														
Safety														
Caregiver ability to safety plan for self and child	3	(2)	1	(1.75)	2	(2)								
Freedom to go about daily life	2	(1)	2	(1.5)	3	(2)	4	(27)	4	(33)	1	(7)	9	16
Risk of harm	2	(2)	2	(1)	1	(1)	6	(40)	3	(25)	8	(57)	17	9=
Feelings of fear	2	(0.5)	2	(0.5)	3	(3)	6	(40)	2	(17)	4	(29)	12	13
Feelings of safety	2	(1)	2	(1)	2	(2)	7	(47)	8	(67)	4	(29)	19	7
Access to safe spaces	2	(2)	1	(1)	2	(2)								
Child's knowledge and understanding of safety	3	(2)	2	(1.5)	3	(2)								
Child's contact with harmful people														
Child's contact with harmful parent/caregiver	2	(1)	2	(2.75)	1	(2)								
Child's contact with other harmful adults	2	(2)	3	(3)	2	(3)								
**Violence, abuse, and maltreatment**														
Child maltreatment														
Child abuse and neglect	2	(1.5)	1	(1)	1	(1)	8	(53)	5	(42)	9	(64)	22	4

Child physical abuse	1	(1)	1.5	(1)	1	(1.25)	6	(40)	2	(17)	6	(43)	14	12=
Neglect of children by caregivers	2	(2)	1	(1)	1.5	(1)								
Child emotional abuse	1	(1)	1.5	(1.75)	1.5	(2)	8	(53)	2	(17)	5	(36)	15	11=
Child sexual abuse	1	(0.5)	1	(1)	1	(1)	8	(53)	4	(33)	6	(43)	18	8=
Child forced marriage	2	(2)	2	(2)	3	(2.5)								
Child sexual exploitation	1	(2.5)	1.5	(1)	2	(2)								
Child's exposure to DVA	2	(1)	1.5	(2)	2	(3)								
Child experience of witnessing violence and abuse	2.5	(3)	2	(1)	3	(3)								
Risk of re-traumatisation	2	(2)	2	(2)	1	(2.25)								
**Intervention outcomes**														
Intervention adverse effects														
Service harms	2	(1)	2	(1)	2	(2)	6	(40)	6	(50)	4	(29)	16	10
Long term negative impact of intervention	2	(2)	2	(1.5)	2	(1)	4	(27)	4	(33)	7	(50)	15	11=

peer review only

For peer review only - http://bmjopen.bmj.com/site/about/guidelines.xhtml

Round 2 and 3 scoring: Domestic violence and abuse

			Round 2						Round 3							
			Survivor		Practitioner		Researcher		Survivor		Practitioner		Researcher			
Area	Domain	Outcome	Median	(IQR)	Median	(IQR)	Median	(IQR)	Score	(%)	Score	(%)	Score	(%)	TOTAL	RANK
**Child health & wellbeing**																
	Child wellbeing															
		Child behaviour	1	(0)	2	(1)	2	(0.5)	3	(27)	5	(29)	6	(55)	14	9=
		Child aggression	1	(1)	2	(2)	2.5	(1.25)								
		Child development	1	(3)	2	(1.5)	2	(1)								
		Child global functioning	3	(2.5)	2	(1.5)	2	(0.75)								
		Child speech and language	2	(3.5)	2	(1)	3	(1.75)								
		Child enuresis or soiling related to behaviour	2	(3.5)	3	(2)	3.5	(1.75)								
		Child emotional health & wellbeing	1.5	(1.75)	1	(1)	2	(1)								
		Child emotional understanding	1.5	(1.75)	2	(2)	3.5	(2)								
		Child ability to concentrate	2.5	(2.5)	3	(2)	3	(1)								
		Child executive functioning	2	(0.75)	2	(1)	3	(1.75)								
		Child anger	2	(2.5)	2	(2.5)	3.5	(1.75)								
		Child sense of calm	2	(1.75)	2	(2.5)	3	(2)								
		Child sense of panic	1	(1.75)	2	(2)	2.5	(1.75)								
		Child happiness	2	(2.5)	2	(2.5)	3	(2)								
		Child perception of recovery from trauma	1	(0.75)	2	(1)	2	(1.75)	2	(18)	7	(41)	1	(9)	10	13
		Child awareness of own body	1	(1)	1.5	(2)	3	(1.75)								
		Child overall wellbeing	2	(1.5)	2	(2)	3	(2)								
		Child satisfaction with life	1.5	(1.75)	2	(1.75)	3	(2)								
		Child attitudes towards antisocial behaviour	2	(2)	2	(3)	3	(1.75)								
	Child mental distress															
		Child overall mental health	1	(0.5)	1.5	(1)	2	(1)	7	(64)	4	(24)	7	(64)	18	5=
		Child anxiety	2	(1.5)	2.5	(2)	2	(1.5)								
		Child depression	2	(1)	2	(1.75)	2	(2)								
		Child dissociation	1	(2)	2	(1)	3	(1.5)								
		Child eating disorders	2	(2)	3	(1)	3	(1.5)								
		Child hypervigilance	1	(1.5)	2	(1.75)	3	(1.5)								
		Child irritability	3	(3)	3	(3.5)	4	(1.5)								
		Child post-traumatic stress	1	(1)	2	(1.75)	1	(1)	4	(36)	8	(47)	8	(73)	20	4
		Child self-harm	1	(1.5)	3	(2)	2	(2)								
		Child substance use	2	(1.5)	2.5	(1)	3	(1)								
		Child suicide	1	(0.5)	1	(2)	1	(1.5)								

For peer review only - http://bmjopen.bmj.com/site/about/guidelines.xhtml

Child trauma	1	(0)	2	(1)	1	(1.5)								
**Caregiver health & wellbeing**														
Caregiver wellbeing														
Caregiver general health & wellbeing	1.5	(1)	2.5	(1)	2	(1.75)	7	(64)	10	(59)	5	(45)	22	3=
Caregiver emotional health & wellbeing	1.5	(1)	2	(1)	2	(2)	3	(27)	8	(47)	4	(36)	15	8=
Caregiver emotional understanding	2	(0.5)	2.5	(1.25)	3	(2)								
Caregiver ability to concentrate	2	(1.25)	3	(2.25)	3	(1)								
Caregiver executive function	3	(1)	2.5	(2.25)	3	(2)								
Caregiver anger	2	(1)	3	(3)	3	(1.5)								
Caregiver sense of calm	2	(2)	2.5	(2.25)	4	(2)								
Caregiver happiness	1	(1.25)	2	(2)	3	(2)								
Caregiver perception of recovery from trauma	2	(2)	2.5	(3)	4	(1.75)								
Caregiver awareness of own body	1	(1.25)	2	(2.25)	3	(1)								
Caregiver satisfaction with life	2	(1.25)	2	(1)	2	(1)								
Caregiver engagement in risky behaviours	2	(1.25)	3	(3)	3	(2)								
Perpetrator health & wellbeing	5	(4)	3	(2.5)	4	(2)								
Caregiver mental distress														
Caregiver overall mental health	1	(0.5)	1	(1)	2	(1)	6	(55)	5	(29)	6	(55)	17	6
Caregiver anxiety	2	(1.5)	3	(1)	3	(1.5)								
Caregiver depression	2	(2)	2	(1)	2	(2)								
Caregiver dissociation	2	(2)	3	(1.5)	3	(1.5)								
Caregiver eating disorders	3	(2)	3	(2)	4	(1)								
Caregiver hypervigilance	2	(1.5)	3	(1.5)	3	(1)								
Caregiver irritability	2	(2.5)	4	(2.75)	4	(0.75)								
Caregiver post-traumatic stress	1	(1)	2	(2)	2	(2)								
Caregiver self-harm	1	(0.5)	3	(2.5)	3	(1.5)								
Caregiver substance use	2	(2)	2	(1.5)	3	(2)								
Caregiver suicide	1	(0)	1	(2)	2	(2)								
Caregiver trauma	1	(0)	1	(1)	2	(2.5)								
**Caregiver relationships & parenting**														
Caregiver-child relationship														
Parent-child relationship	1	(2)	2	(1)	2	(1)	7	(64)	14	(82)	8	(73)	29	2
Parent-child interaction	1	(1.5)	2	(1.5)	2	(1)								
Parent-child conflict	2	(1)	3	(1.5)	3	(1)								
Child's feelings about parents	2	(1)	2	(1.5)	2	(1)								
Relationship with birth family	4	(1)	4	(2.5)	3	(1.5)								
**Home environment & household**														
Family function														
Family functioning and stability	2	(2)	2	(2)	3	(1)	6	(55)	11	(65)	5	(45)	22	3=
Family relationships	2	(1.5)	1.5	(1)	2	(1)	3	(27)	11	(65)	4	(36)	18	5=
Family emotional environment	1	(1)	2	(1.25)	3	(1.75)	4	(36)	8	(47)	3	(27)	15	8=
Family communication	2	(1)	2.5	(3)	3	(1.75)								

Missing episodes	4	(1)	4	(2.75)	3	(1)								
**Safety, feelings & knowledge related to violence and abuse**														
Safety														
Caregiver ability to safety plan for self and child	2	(1.25)	2	(1.5)	2	(2)								
Freedom to go about daily life	1	(0.25)	2	(2)	2	(2)	4	(36)	6	(35)	4	(36)	14	9=
Risk of harm	1	(1)	1	(1)	1	(1)	8	(73)	13	(76)	9	(82)	30	1=
Feelings of fear	1	(1)	2	(1.5)	2	(1.25)	2	(18)	6	(35)	4	(36)	12	11
Feelings of safety	1	(0.5)	1	(1)	1.5	(2)	7	(64)	15	(88)	8	(73)	30	1=
Access to safe spaces	1	(1)	2	(1)	1.5	(1)	5	(45)	4	(24)	4	(36)	13	10
Child's knowledge and understanding of safety	1.5	(1.25)	2	(1)	2	(2)								
Child's thoughts & knowledge about their experiences of CM or DVA														
Child knows DVA is not their fault	1	(0.5)	1	(1)	1	(1)	3	(27)	6	(35)	2	(18)	11	12
Child's ability to make sense of DVA experiences	1	(2)	2	(2)	2.5	(1)								
Child feels validated	2	(2)	1	(2)	2	(2)								
Child's sense that they can move on from violence	1	(1)	1	(1.25)	1.5	(1)	4	(36)	2	(12)	2	(18)	8	15=
Child's ability to identify safe relationships	1	(2)	2	(1.25)	1	(1)								
Child's perception of others' judgements of abuse	2	(1.5)	3	(2.25)	4	(1.75)								
Child's knowledge about DVA	1	(0.5)	1	(1.25)	2	(0.75)	7	(64)	4	(24)	5	(45)	16	7
Child's understanding of impact of violence	2	(2)	2	(2)	3	(1.75)								
Child's understanding of consent	1	(0.5)	2	(1)	2	(2)	4	(36)	2	(12)	3	(27)	9	14
**Violence, abuse, and maltreatment**														
Child maltreatment														
Child abuse and neglect	1	(0)	2	(1)	1	(1.25)	6	(55)	6	(35)	6	(55)	18	5=
Child physical abuse	1	(1)	1.5	(1.25)	1.5	(2)								
Neglect of children by caregivers	1	(0.5)	1.5	(1.25)	1	(1)								
Child emotional abuse	1	(0.5)	1	(1)	2	(2)	2	(18)	4	(24)	2	(18)	8	15=
Child sexual abuse	1	(0)	1	(2.25)	1	(1)								
Child forced marriage	1	(0.5)	2	(2.5)	1	(2)								
Child sexual exploitation	1	(0)	2	(2.25)	1	(1.25)								
Child's exposure to DVA	1	(0)	1	(0)	2	(1.25)								
Child experience of witnessing violence	1	(1)	1.5	(1)	2	(2)	5	(45)	6	(35)	4	(36)	15	8=
Risk of re-traumatisation	1	(0)	2	(1)	2	(3)								
Domestic violence and abuse between caregivers														
Domestic violence and abuse	1	(0)	1	(0)	1	(1)								
Economic abuse	1	(1)	2	(1.5)	2	(2)								
Emotional abuse	1	(0)	1	(2)	1.5	(1.25)								
Physical abuse	1	(1)	2	(2)	1	(1)								
Sexual abuse	1	(0)	2	(2)	1	(1.25)								

Coercive control	1	(0)	1	(1.5)	1	(1)
Experience of harassment	1	(1)	2	(2)	2	(1)
Harm to family pets	1	(2)	3	(2.5)	2	(1)
Injury	1	(0)	2	(2.5)	2	(1)
Reproductive coercion	1	(1.5)	2	(2)	2	(1.25)
Perpetrator rehabilitation/desistance	1	(3)	1	(2)	2	(1)
Perpetration of DVA	1	(1.5)	1	(2)	1.5	(1)
Risk of re-traumatisation	1	(1)	2	(2)	2	(1.25)
**Intervention outcomes**						
Intervention adverse effects						
Service harms	1	(0.25)	2	(1.5)	2	(0.25)
Long term negative impact of intervention	1.5	(2)	2	(3)	2	(1.5)

For peer review only - http://bmjopen.bmj.com/site/about/guidelines.xhtml

Supplement 9: DVA consensus workshop consensus statement

Domestic Violence Core Outcome Set consensus statement

Following the multi-stakeholder workshop on 28th September 2021 the workshop participants agreed to change the outcome *child overall mental health* to *child emotional health and wellbeing* which was part of the original longlist of candidate outcomes. This decision emerged from discussions that emphasised concerns around: i) the medicalisation of young people's distress in response to experiences of DVA; ii) the consensus that emotional health and well-being more readily captures positive functioning and/or adaption rather than emphasising deficits (that some participants felt were implicit in the term *mental health);* iii) the broader applicability of emotional health and wellbeing to sectors outside of health care: the voluntary sector, education and social care. It was agreed that emotional health and wellbeing could encompass specific mental health outcomes such as psychiatric symptoms and diagnoses.

Original outcome

- **Overall mental health** (includes psychiatric symptoms, psychological distress, psychological wellbeing, mental well-being, mental health problems, signs of these including admissions/healthcare or service contacts)

Proposed change:

- **Emotional health & wellbeing** (includes emotions, mood, internalizing problems or symptoms; emotional regulation; emotional security; emotional numbness)

view only

Supplement 10: Consensus workshop outcome rankings

Child maltreatment consensus workshop outcome rankings by number of votes

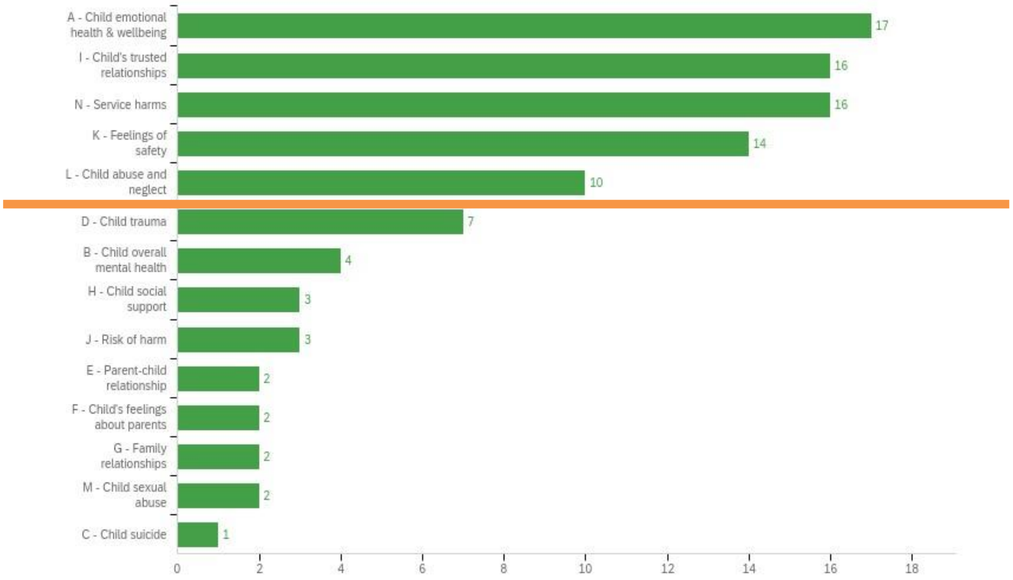

Domestic violence and abuse consensus workshop outcome rankings by number of votes

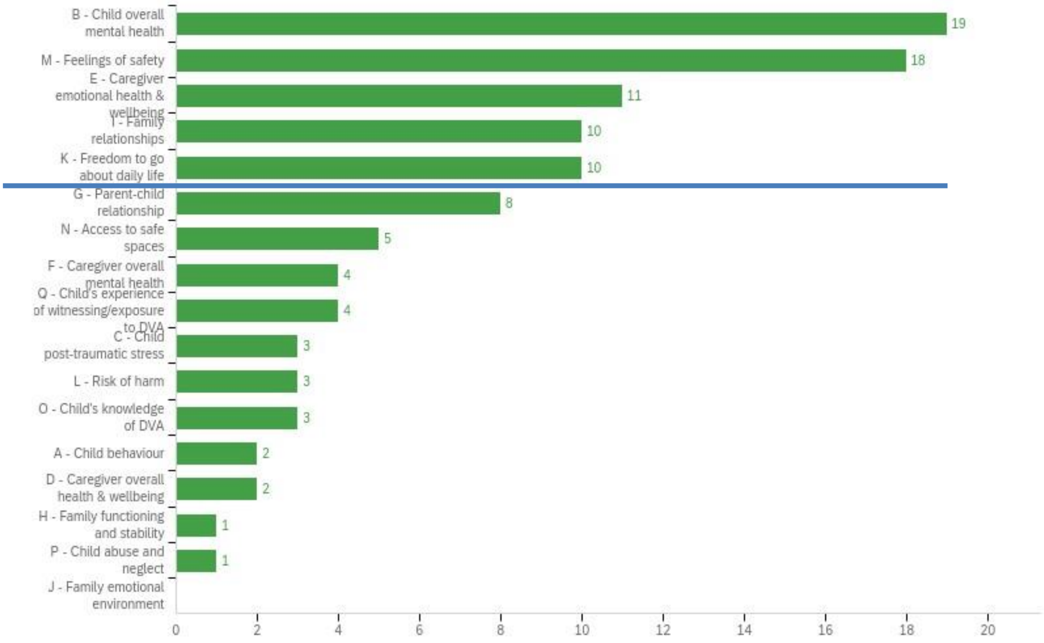

Supplement 11: Summary of discussion about implications of COS for underserved groups

1) Language and meaning

The meaning of concepts related to outcomes was discussed and whether these are understood in the same way by service users and professionals. There was concern about how language could be adapted for all age groups of children without loss of meaning. The cross-cultural understanding of concepts was raised and how accurate translation can be ensured across global settings. Finally, there was discussion about gender in relation to how child behaviour is understood and how violence is analysed.

2) Rights and discrimination

Overall, there was a preference for a rights-based focus as well as understanding of inequalities in any outcome measurement tool. Participants reflected on the impact of socio-economic exclusion and destitution and how particularly minoritised groups, such as migrant children, could be protected. There was a focus on how to centre the child's voice in any understanding of outcomes. In terms of outcome measurement/assessment there was discussion around how to reduce practitioner bias against marginalised groups, including the misuse of mental health diagnosis.

3) Using measurement tools

Concerns on the practical delivery of any measures used for the core outcome sets, both in terms of the tools themselves and the staff who might be administering the tools.

For peer review only - http://bmjopen.bmj.com/site/about/guidelines.xhtml

Core Outcome Set-STandards for Reporting: The COS-STAR Statement Checklist

SECTION/TOPIC	ITEM No.	CHECKLIST ITEM	REPORTED ON PAGE NUMBER
TITLE/ABSTRACT			
Title	1a	Identify in the title that the paper reports the development of a COS	1
Abstract	1b	Provide a structured summary	2
INTRODUCTION			
Background and Objectives	2a	Describe the background and explain the rationale for developing the COS.	3
	2b	Describe the specific objectives with reference to developing a COS.	3
Scope	3a	Describe the health condition(s) and population(s) covered by the COS.	3
	3b	Describe the intervention(s) covered by the COS.	3
	3c	Describe the setting(s) in which the COS is to be applied.	3
METHODS			
Protocol/Registry Entry	4	Indicate where the COS development protocol can be accessed, if available, and/or the study registration details.	4
Participants	5	Describe the rationale for stakeholder groups involved in the COS development process, eligibility criteria for participants from each group, and a description of how the individuals involved were identified.	4
Information Sources	6a	Describe the information sources used to identify an initial list of outcomes.	5
	6b	Describe how outcomes were dropped/combined, with reasons (if applicable).	5-6
Consensus Process	7	Describe how the consensus process was undertaken.	6-7
Outcome Scoring	8	Describe how outcomes were scored and how scores were summarised.	6-7, online supplementary materials
Consensus Definition	9a	Describe the consensus definition.	6-7, online supplementary materials
	9b	Describe the procedure for determining how outcomes were included or excluded from consideration during the consensus process.	6-7, online supplementary materials
Ethics and Consent	10	Provide a statement regarding the ethics and consent issues for the study.	4,5,6
RESULTS			
Protocol Deviations	11	Describe any changes from the protocol (if applicable), with reasons, and describe what impact these changes have on the results.	7, online supplementary materials

For peer review only

Participants	12	Present data on the number and relevant characteristics of the people involved at all stages of COS development.	Online supplementary materials
Outcomes	13a	List all outcomes considered at the start of the consensus process.	Link to longlist in repository
	13b	Describe any new outcomes introduced and any outcomes dropped, with reasons, during the consensus process.	7
COS	14	List the outcomes in the final COS.	8
DISCUSSION			
Limitations	15	Discuss any limitations in the COS development process.	9
Conclusions	16	Provide an interpretation of the final COS in the context of other evidence, and implications for future research.	9-10
OTHER INFORMATION			
Funding	17	Describe sources of funding/role of funders.	10
Conflicts of Interest	18	Describe any conflicts of interest within the study team and how these were managed.	10

From: Kirkham JJ, Gorst S, Altman DG, Blazeby JM, Clarke M, Devane D, et al. (2016) Core Outcome Set–STAndards for Reporting: The COS-STAR Statement. PLoS Med 13(10): e1002148. https://doi.org/10.1371/journal.pmed.1002148

For peer review only

BMJ Open

Child and family-focused interventions for child maltreatment and domestic abuse: Development of core outcome sets

Journal:	*BMJ Open*
Manuscript ID	bmjopen-2022-064397.R1
Article Type:	Original research
Date Submitted by the Author:	12-Aug-2022
Complete List of Authors:	Powell, Claire; UCL, Institute of Child Health<br>Feder, Gene; University of Bristol, Community based medicine<br>Gilbert, Ruth; UCL Institute of Child Health, Centre for Paediatric Epidemiology and Biostatistics<br>Paulauskaite, Laura ; UCL, Institute of Education<br>Szilassy, Eszter; University of Bristol, Centre for Academic Primary Care, Population Health Sciences, Bristol Medical School<br>Woodman, Jenny; UCL, Social Research Institute<br>Howarth, Emma; University of East London, School of Psychology
**Primary Subject Heading**:	Public health
Secondary Subject Heading:	Evidence based practice, General practice / Family practice, Health services research, Mental health
Keywords:	Child protection < PAEDIATRICS, Community child health < PAEDIATRICS, PUBLIC HEALTH, SOCIAL MEDICINE, PRIMARY CARE

SCHOLARONE™
Manuscripts

I, the Submitting Author has the right to grant and does grant on behalf of all authors of the Work (as defined in the below author licence), an exclusive licence and/or a non-exclusive licence for contributions from authors who are: i) UK Crown employees; ii) where BMJ has agreed a CC-BY licence shall apply, and/or iii) in accordance with the terms applicable for US Federal Government officers or employees acting as part of their official duties; on a worldwide, perpetual, irrevocable, royalty-free basis to BMJ Publishing Group Ltd ("BMJ") its licensees and where the relevant Journal is co-owned by BMJ to the co-owners of the Journal, to publish the Work in this journal and any other BMJ products and to exploit all rights, as set out in our licence.

The Submitting Author accepts and understands that any supply made under these terms is made by BMJ to the Submitting Author unless you are acting as an employee on behalf of your employer or a postgraduate student of an affiliated institution which is paying any applicable article publishing charge ("APC") for Open Access articles. Where the Submitting Author wishes to make the Work available on an Open Access basis (and intends to pay the relevant APC), the terms of reuse of such Open Access shall be governed by a Creative Commons licence – details of these licences and which Creative Commons licence will apply to this Work are set out in our licence referred to above.

Other than as permitted in any relevant BMJ Author's Self Archiving Policies, I confirm this Work has not been accepted for publication elsewhere, is not being considered for publication elsewhere and does not duplicate material already published. I confirm all authors consent to publication of this Work and authorise the granting of this licence.

For peer review only

Child and family-focused interventions for child maltreatment and domestic abuse: Development of core outcome sets

Claire Powell* [1], Gene Feder [2], Ruth Gilbert [1], Laura Paulauskaite [3], Eszter Szilassy [2], Jenny Woodman [3], Emma Howarth [4]

Affiliations

[1] Institute of Child Health, UCL, 30 Guilford St, London, UK WC1N 1EH [2] School of Social and Community Medicine, University of Bristol 39 Whatley Road, Bristol, UK BS8 2PS [3] Institute of Education, UCL, 27/8 Woburn Square, London, UK WC1H 0AA [4] School of Psychology, UEL, University Way, London, UK E16 2RD

*Corresponding author

Email: c.powell@ucl.ac.uk

Keywords: domestic abuse, child maltreatment, core outcome sets, interventions, family health

Word count: 3699

For peer review only - http://bmjopen.bmj.com/site/about/guidelines.xhtml

Abstract

Background

The current evidence for child maltreatment and domestic violence and abuse interventions is limited by the diversity of outcomes evaluated and the variety of measures used. The result is studies that are difficult to compare and lack focus on outcomes reflecting service user or provider priorities.

Objective

To develop core outcome sets (COS) for evaluations of child and family-focused interventions for: i) child maltreatment (CM); and ii) domestic violence and abuse (DVA).

Design

We conducted a two-stage consensus process. Stage one: A longlist of candidate outcomes across CM and DVA was developed through rapid systematic reviews of intervention studies, qualitative and grey literature; stakeholder workshops; survivor interviews. Stage two: Three-panel, three-round e-Delphi surveys for CM and DVA with consensus meetings to agree the final COSs.

Participants

287 stakeholders participated in at least one stage of the process (i.e. either CM or DVA COS development): workshops (n=76), two e-Delphi surveys (n=168) and consensus meetings (n=43). Stakeholders included CM and DVA survivors, practitioners, commissioners, policymakers, researchers.

Results

Stage one identified 335 outcomes categorised into nine areas and 39 domains. Following stage two, the final five outcomes included in the CM-COS were: child emotional health and wellbeing; child's trusted relationships; feelings of safety; child abuse and neglect; service harms. The final five outcomes in the DVA-COS were: child emotional health and wellbeing; caregiver emotional health and wellbeing; family relationships; freedom to go about daily life; feelings of safety.

Conclusions

We developed two core outcome sets for CM and DVA with two common outcomes (child emotional health and wellbeing; feelings of safety). The COSs reflect shared priorities amongst service users, providers, and researchers. Use of these COSs across trials and service evaluations for children and families affected by CM and DVA will make outcome selection more consistent and help harmonise research and practice.

Article summary

Strengths and Limitations

- This study used a systematic consensus process which involved a large number of practitioners, policymakers, researchers and adult survivors of violence and abuse.
- The use of multiple forms of engagement enabled participants to take part in a range of ways: workshops, interviews, online survey.
- Survivor involvement was central to the process and enabled us to shape the study as we carried it out.

For peer review only

For peer review only - http://bmjopen.bmj.com/site/about/guidelines.xhtml

- Given the impact of the covid-19 pandemic we were unable to reach as many frontline DVA and CM organisations as we intended.
- Our reach into minoritised communities was not as extensive as we would have liked so survivors in the study do not reflect the extent of diverse communities in the UK. We sought explicit feedback on this limitation in the final stage of the study.

Introduction

There is insufficient high-quality evidence for the effectiveness of child maltreatment (CM) and domestic violence and abuse (DVA) interventions for improving child and family outcomes.[1–3] This means service providers do not know which interventions are most useful or might potentially cause harm: this uncertainty could discourage identification of CM or DVA. [4] It is difficult to compare and synthesise studies due to inconsistent outcome reporting and the range of measures used. Even widely reported outcomes such as depression or experience of violence are measured in varied ways,[4,5,6] including across evaluations of similar interventions such as psychological therapies.[7] Current global guidance on evaluation of CM programmes does not promote the use of comparable outcomes. [8]

Decisions about which outcomes to measure tend to be led by researchers, meaning those selected may not be relevant to service users and providers. For example, Howarth et al (2016) reported that clinical trials of interventions for CM or DVA prioritised symptoms and diagnoses, which differed from priorities of affected children and families who emphasised wider outcomes related to everyday wellbeing and functioning.[9] Similarly, work with service users and providers of domestic violence perpetrator programmes found broader definitions of 'success' than are traditionally measured. [10]

Inconsistent or inappropriate outcome reporting and measurement result in research wastage [11] and uncertainty about what interventions work or do not work, and for whom. Consequently, funding might be wasted on ineffective or even harmful interventions.

One way to address these challenges is development of core outcome sets, a minimum set of outcomes which are reported across all clinical trials, and potentially practice-based evaluations as well. COSs should be developed with a standardised consensus process to identify outcomes important to all stakeholders, resulting in the selection of the core outcomes. [10] The aim of a COS is to increase the consistency of outcome measurement and reporting in order that evidence accumulates in helpful ways whilst minimising selective outcome reporting, and ensuring the outcomes are meaningful to service users and providers.

Aim

Our aim was to develop two core outcome sets to be used in evaluations of child, parent, or whole-family interventions for: i) child maltreatment (CM); and ii) domestic violence and abuse (DVA). For DVA, we focused on interpersonal violence and abuse between parents/caregivers which is the most prevalent form of DVA. [12] By developing the two sets in parallel we intended to bring together two areas of practice that may result in a joint service response, [13,14] although they often operate separately. [15] It is acknowledged that collaboration between researchers and practitioners in this area is vital [16] and that the DVA and CM research agendas should be brought together, [17] not least because exposure to DVA is considered by many to be a form of CM, and in up to 60% of homes with DVA, there may also be CM present. [18]

For peer review only - http://bmjopen.bmj.com/site/about/guidelines.xhtml

Scope

The core outcome sets were developed for research and evaluation of any psychosocial interventions (as defined by the Institute of Medicine [19]) for children and families with experience of or at risk of experiencing CM or DVA. We included any intervention globally that aimed to improve child outcomes through targeting parents or family members. The target population was children aged under 19 years with current or previous experience of CM or DVA. Included interventions could be delivered in any setting, to an individual, dyad or group, and any combination of child, parent/caregiver, family groups alone or in combination. To be in scope, those eligible for the intervention had to have been exposed to/experienced CM or DVA or to be at increased risk of CM or DVA.

Methods

For peer review only

We registered the study with the Core Outcome Measures in Effectiveness Trials (COMET) initiative [20] and published the study protocol.[21] Ethics approval was provided by University College London's Research Ethics Committee for involving research participants (17893/001 & 002) and we were guided by a steering group of eight professionals. (For details see acknowledgments.)

Following COMET methodology,[22] we used a two-stage approach. In Stage 1 we devised a longlist of candidate outcomes from stakeholder consultation, qualitative interviews, trials, and the wider literature. In Stage 2, two e-Delphi consensus processes based on this longlist resulted in two core outcome sets. This report of the process and the findings follows COS-STAR guidelines.[23]

See Figure 1 for study flow diagram.

We decided to run Stage 1 jointly for CM and DVA to develop a comprehensive longlist of outcomes that could be relevant to all family-focused solutions and to reflect the high levels of co-occurrence between CM and DVA. However, given differences in current approaches to CM and DVA interventions, for example social care interventions for CM and women's refuge interventions for DVA, we ran Stage 2 as two separate e-Delphi processes to understand differing priorities between these two fields.

Patient and public involvement

We consulted two survivor advisory groups, one comprising adult survivors of domestic violence and abuse, the other comprising young adult care leavers. (For details see acknowledgements). We involved both groups after their participation in the stakeholder workshops and they provided advice on the accessibility and appropriateness of study materials, for example survey wording. They also designed a sensitivity protocol for the qualitative interviews to ensure that these were trauma-informed, consulted on the facilitation of and participated in the consensus workshops. (See online supplement 1 for sensitivity protocol.)

Participants

We recruited from the following groups of participants for the stakeholder workshops (Stage 1), qualitative interviews (Stage 1) and for the e-Delphi survey and consensus workshops (Stage 2):

a. Survivors: UK-based adults with lived experience of CM/DVA in childhood or as the parent of a child who experienced CM/DVA. Survivors did not have to have used any

services or interventions to take part, and were recruited through survivor networks, charities and university patient and public involvement (PPI) groups.

b. Practitioners: UK-based professionals working in frontline (i.e. delivering services) or second tier (i.e. supporting service-providers) CM/DVA organisations, local authority commissioning or working in any policy capacity in this area. We recruited through research team networks, directly approaching organisations, and social media.

c. Researchers – English-speaking academic researchers based in high-income country universities or independent research organisations in the UK and internationally. We recruited through research networks, directly approaching researchers or teams, and social media.

(See online supplement 2 for Stage 1 and Stage 2 participant details.)

Stage 1: Information sources

In Stage 1 we identified candidate outcomes from three sources: i) rapid evidence reviews, ii) consultation with key stakeholders, and iii) qualitative interviews.

i) Rapid evidence reviews

We carried out a series of rapid reviews separately for CM and DVA, updating previous systematic reviews. [1,2] (See online supplement 3 for inclusion criteria, online supplement 4 for review flow charts and for the search strategies see protocol. [21]) For the intervention literature we searched Medline, Embase, PsycInfo, Cochrane and Web of Science from Jan 2014 to May 2019. For the qualitative literature, ASSIA, CINAHL, GoogleScholar, PsycInfo and SSCI were searched from October 2015 (DVA) and July 2014 (CM) until October 2019. Differences in dates reflect differences in original reviews being updated. The grey literature review was carried out for CM and DVA simultaneously, to identify additional outcomes. We searched websites of relevant organisations (see online supplement 5 for full list) and NICE Evidence Search and Open Grey databases.

Two researchers (CP and EH) dual screened 400 title/abstracts for each of the DVA and CM (200 from the intervention literature searches and 200 from the qualitative literature searches) as a consistency check. The first 10% of full texts were dual screened (CP), with disagreements resolved by discussion as a further consistency check, prior to screening of the rest of the full texts by a single researcher. Study details and outcomes were extracted by a single researcher with 5% cross-checked by the lead researcher. Participant quotations and author-identified themes from the qualitative studies and grey literature were extracted into a matrix to identify outcomes. All outcomes identified from the reviews were added to the longlist and deduplicated as part of the process.

ii) Consultation with key stakeholders

We carried out two half day workshops (19th June 2019 for DVA, 5th September 2019 for CM) in which a total of 76 participants generated a list of candidate outcomes in small groups. The research team and external speakers gave presentations in the workshops on CM and DVA intervention research and interim findings from the rapid evidence reviews. These were followed by small group discussions on definitions of key terms and outcomes. All outcomes from small-group discussions were deduplicated and added to the longlist.

iii) Qualitative interviews

To identify additional outcomes, including those that survivors would like to be measured but are not currently, one researcher (CP) carried out 10 semi-structured interviews in August to December

For peer review only - http://bmjopen.bmj.com/site/about/guidelines.xhtml

2020 with survivors of child maltreatment and/or domestic violence and abuse. The interviews were designed in consultation with the study survivor advisory groups and participants gave written, informed consent before each interview, with the option to withdraw at any stage. Interviews lasted 40 - 65 minutes, were audio recorded and transcribed. One interviewee chose to participate by email. Two researchers (CP and LP) extracted outcomes from the interview transcripts, using the questions: i) Is this an outcome? ii) Is this related to an outcome? Outcomes were cross-checked with the longlist and added if they were not already present.

Stage 1: Longlist and taxonomy development

A single longlist was produced comprising all outcomes gathered from CM and DVA information sources (i.e. the rapid evidence reviews, stakeholder consultations and qualitative interviews). Producing the final longlist involved an iterative process of 1) de-duplicating outcomes produced within each information source (e.g. the consultation workshops); 2) combining outcome lists from information sources to form a single longlist; 3) grouping similar outcomes and developing categories to label groups of similar outcomes; 4) deduplicating outcomes across the longlist; 5) combining specific outcome indicators together. Exact duplicates were dropped first, and similar outcomes were reworded to reflect broader meanings. For example, the child health outcome 'sleep' was created by combining: 'amount of sleep', 'quality of sleep', 'experience of nightmares', 'sleep routine', 'insomnia' and 'sleep-walking'. This process was carried out by two researchers (CP and EH) cross-checking and refining the longlist. At several points, the PPI, expert advisory groups, and wider research team provided feedback on specific sections of the list and gave input on the level of detail for the outcomes, for example creating one 'sleep' outcome as described above, rather than two.

We organised the longlist of outcomes into a taxonomy using an iterative team-based approach. The initial categories, based on participant discussion and notes produced in the stakeholder workshops, were structured using Bronfenbrenner's ecological model as a framework. [24] Related theoretical frameworks for CM and DVA were consulted [25–27] and used to further refine the taxonomy, so it accurately reflected the longlist. The taxonomy was finalised by the expert advisory group and the survivor groups reviewed the final categories and outcome wording for sense and missing outcomes. The final longlist and taxonomy were used as the basis for the questionnaires in the e-Delphi survey and final consensus workshop.

See Figure 2 for outcome identification and longlist compilation flowchart

Stage 2: Consensus process

In Stage 2 participants took part in a three-panel three-round e-Delphi survey to reach consensus. The three panels were: survivor, researcher, practitioner (as described above). Participants were informed that the operationalisation and measurement of each indicator would take place in a subsequent, as yet unfunded study, and reflects the development process of other outcome sets. We used Qualtrics software (Qualtrics, Provo, UT) to collect the data. PPI groups reviewed and edited the survey questions and format for clarity. Survey questions were piloted with one survivor and two team members not involved in its design (LP and RG). Two parallel surveys were conducted, one for CM and one for DVA. Participants gave informed consent as part of the survey, with the option to withdraw their data up to two weeks after completion. (See online supplement 6 for survey.)

See Figure 3 for Stage 2 e-Delphi consensus process and inclusion cut-offs

For peer review only

We asked survivor participants whether they were survivors of CM or DVA. If they had experienced both, they chose whether to participate in the CM or the DVA survey. We assigned professionals to the research or practitioner panels based on information these participants supplied. Although we ran the two surveys in parallel, the first round of the CM and DVA surveys were the same. However, the second round (and subsequently third round and consensus workshop) differed according to the domains and outcomes selected by panels in the CM and DVA surveys. The first round of both surveys asked participants to select broad outcome domains to reduce the longlist. The second round offered the more specific outcome indicators from the selected domains, and the third round offered the remaining outcome indicators. Outcome domains and indicators were presented in a random order to participants in each round to reduce bias. At the end of each survey we had two shortlists of highly prioritised outcomes, one for CM and one for DVA.

The final stage of the consensus process was online consensus workshops with participants representing the three panels: survivor, practitioner, researcher. Covid-19 restrictions meant the workshops could not be held in person, therefore we invited 24 participants to each workshop (i.e. one for DVA and one for CM), drawing on current recommendations for online consensus development meetings from the James Lind Alliance. [28] Information on the purpose of the workshop and the relevant shortlists were sent to all participants ahead of the workshop. Participants discussed their top and bottom three outcomes in two rounds of breakout group sessions, before voting on which to include.

The final core outcome sets included only those outcomes that at least half of workshop participants voted to include. There was a final plenary discussion on the core outcome sets and the implications for marginalised and under-heard groups. To maintain survivor confidentiality, the workshops were not recorded; however, workshop facilitators made detailed notes of discussions. An external facilitator led the workshops to maintain impartiality and members of the research team (CP, EH, ES) acted as neutral co-facilitators of breakout groups. A qualified counsellor was available to speak to survivors during the workshops and for a week afterwards. Further details about how we adapted the process to be trauma-informed will be described in future work.

Results

This study was completed according to the study protocol, any changes were made in response to the evolving COVID-19 situation (e.g. holding interviews and workshops online) and to reduce participant burden (e.g. increasing the survey consensus threshold because the levels of consensus across outcomes was higher than expected). (For full details see online supplement 7).

Stage 1

Six rapid evidence reviews, two stakeholder workshops and ten survivor interviews identified 335 unique outcomes. (For longlist see https://osf.io/yhnfq/) The outcomes taxonomy comprised 39 domains nested within nine broad areas which were: 1) child health & wellbeing; 2) caregiver health & wellbeing;3) caregiver relationships and parenting; 4) home environment and household; 5) social support and peer relations; 6) community resources and institutions; 7) safety, feelings and knowledge related to violence and abuse; 8) violence, abuse and maltreatment; 9) intervention outcomes. (For the full taxonomy see https://osf.io/9htz4/)

For peer review only - http://bmjopen.bmj.com/site/about/guidelines.xhtml

Stage 2

We recruited a total of 80 participants for the CM e-Delphi survey and 90 participants for the DVA e-Delphi survey (not all participants took part in all rounds). We did not reach our recruitment target of 30 participants in each panel; we think this was primarily due to the impact of Covid-19 and associated delays which resulted in the survey running over the school summer holidays.

See Figure 4 for the distribution of participants by round in Stage 2.

Outcomes were dropped at each stage of the e-Delphi. No new outcomes were added throughout the survey process, however suggestions offered by participants (in free text within the survey) were cross-checked with the longlist and incorporated as details into pre-existing outcomes, for example ‘safety within court proceedings’ was added to the ‘safety’ outcome. Two domains were merged because of feedback from the survey – ‘child specific exposure to DVA’ was incorporated into ‘child maltreatment’.

See Figure 5 for the selection of outcomes

(See online supplement 8 for all ratings of survey items by panel and by round.)

Given ongoing consultation throughout the process regarding practitioner and researcher concerns about the potential size of the COS, the research team agreed that the core outcome set would be limited to five outcomes, with discussion around any tied scores. Practitioners and researchers were concerned about the possible burden if the outcomes in the final COS were very different from those already collected in the context of service monitioring. Through informal discussions with collaborators, five was agreed as enough to capture and compare shared outcomes but a small enough number to be feasible to implement in research and evaluation. In the event, both consensus workshops yielded five outcomes that scored higher than the rest and met our inclusion (criteria that at least 50% of participants voted for them).

Following the final round of discussion in the DVA consensus meeting, it was agreed to change *Child mental health* to *Child emotional health & wellbeing* because participants felt this reflected both survivor and practitioner perspectives in a more holistic way. Workshop participants agreed to a consensus statement to document this change (see online supplement 9).

Core outcome sets

The final core outcome sets, each comprising five outcomes are as follows:

Child Maltreatment COS

1) Child emotional health & wellbeing: includes emotions, mood, internalising problems, emotional regulation, emotional security, and emotional numbness.

2) Child’s trusted relationships: includes with friends, family, other adults; network of trust adults – includes access to, quantity and quality, in and out of school.

3) Service harms: includes general harmful service response, iatrogenic harm, replication of abusive dynamics in therapy, re-traumatisation, re-victimisation, secondary abuse, intervention adding to self-blame in women, traumatic physical procedures.

4) Feelings of safety: for non-abusive parent and child; global safety, including psychological, physical, body, family, neighbourhood around perpetrator, at home, at school, in the community, on social media, from abusive individuals, from child removal, from court proceedings.

For peer review only - http://bmjopen.bmj.com/site/about/guidelines.xhtml

5) Child abuse and neglect: includes occurrence, recurrence, risk, type.

Domestic Violence and Abuse COS

1) Child emotional health & wellbeing: includes emotions, mood, internalising problems, emotional regulation, emotional security, and emotional numbness.

2) Feelings of safety: for non-abusive parent and child; global safety, including psychological, physical, body, family, neighbourhood around perpetrator, at home, at school, in the community, on social media, from abusive individuals, from child removal, from court proceedings.

3) Caregiver emotional health & wellbeing: includes emotional functioning, emotional reactions, emotions, emotional self-regulation, control over emotions, ability to connect to emotions, mood, frame of mind, general sense of well-being.

4) Family relationships: includes overall family relationships and functioning, quality, and type of relationships, feeling closer as a family, family conflict resolution, feeling closer to children, changes after leaving abusive partner; sibling relationships including after separation; child relationship with birth and foster/adoptive families.

5) Freedom to go about daily life: includes ability to get home safely from school/ work/ friends/ family etc.

See Figure 6 to see the COSs as an infographic.

The outcomes are ordered by number of workshop votes. (For ranking of all discussed outcomes see online supplement 10)

Core outcome sets and underserved groups

Following agreement on the final sets the afternoon discussion focused on who might be excluded by the core outcome sets. The key groups included: neurodiverse and disabled children and families, families living in poverty, and ethnic and racialised minorities. Discussion centred on three topics: 1) language and meaning; 2) rights and discrimination; 3) practical delivery of relevant measurement tools. (For further details see online supplement 11.)

Discussion

We developed two core outcome sets using consensus methods for child and family-focused interventions for CM and DVA. The scope of the outcome sets was broad, including health and wellbeing, safety, and relationship outcomes. The core outcome sets had two outcomes in common - *child emotional health and wellbeing* and *feelings of safety* – reflecting shared priorities between service users and providers across CM and DVA. Furthermore, three of the eight core outcomes are not currently reported in the CM or DVA intervention literature: *child's trusted relationships*, *freedom to go about daily life* or *service harms*. This emphasises the importance of a consensus process involving service users and providers, to capture outcomes critical to all stakeholders, not just researchers.

This is the first time that COSs have been developed for CM and DVA, reflecting a methodological leap forward for the fields. The overlapping nature of the COSs reflects growing recognition that these experiences co-occur within families and that service and therapeutic responses need to

For peer review only - http://bmjopen.bmj.com/site/about/guidelines.xhtml

consider both CM and DVA. This is novel given research literatures and service provision have largely developed in parallel.

The next step is to develop consensus and guidance about how best to further operationalise and measure outcomes consistent with the domains we identify, three of which have not before been included in any quantitative research or service evaluations. In the longer term, consensus around the best measures to use to capture outcomes will facilitate meta-analyses of outcomes which require the same measure to have been used across studies. In the shorter term, our core outcome sets can be used as a framework for service evaluation and research. Even if different measures are used across service evaluations, narrative methods can be used to synthesise findings across studies within a core outcome. This represents a significant step forward.

Additionally, using our core outcome sets as a guide to both developing and evaluating interventions will mean that interventions in this field will have a better chance of making a meaningful difference to the lives of those experiencing DVA or CM given they are aligned with the proirities of representatives of these groups. This two-stage process of determining 'what to measure' followed by 'how' reflects the development process of many other COS and is dictated by pragmatic considerations such as securing funding to undertake both parts and also the desire to communicate useful interim findings to stakeholders.

The key strength of our COS development process was the scope of engagement: we involved survivors at every stage both as participants and in an advisory capacity, and we involved professionals from a range of organisations across the non-governmental and statutory sectors. As a result, we identified a broad range of possible outcomes, including those that are not currently measured but nonetheless considered important. The high level of consensus about the importance of many outcomes was unexpected (and resulted in changes to the protocol), highlighting that there are many shared priorities across survivors, service providers and researchers. The outcomes identified in this study are consistent with findings from engagement with a range of stakeholders for DVA perpetrator programmes. [10]

By developing COS in parallel for CM and IPV, and finding important areas of overlap, such as safety, our findings will help underpin evaluations of family focussed interventions. The shared priorities across the COSs highlight the joint importance of health and wellbeing outcomes rather than a focus on mental health and psychopathology, as well as relationships and safety. The stakeholder involvement and transparent development process have resulted in cross-sector engagement which will be useful for future implementation. Without extensive survivor engagement and reviewing literature beyond clinical studies the core outcome sets would not have reflected all stakeholder priorities.

Limitations

We faced challenges at every stage because of the Covid-19 pandemic and because we were adapting a process developed to support the evaluation of clearly defined medical and surgical interventions, rather than complex psychosocial interventions. Covid-19 restrictions meant we were unable to access as many frontline services as we intended, and it likely affected the survey drop-out rates. Moving online meant we only included survivors who could access the internet, so we did not reach the most marginalised groups of survivors. This limitation needs to be addressed in any future development of CM and DVA core outcome sets.

For these core outcome sets to be used in applied research, the concepts need further refinment and operationalisation via an explicit consensus process (with survivors, practitioners and

For peer review only - http://bmjopen.bmj.com/site/about/guidelines.xhtml

researchers) to identify appropriate measurement tools. [29] The core outcome sets as they currently stand represent the first stage of harmonising outcome measurement within and across CM and DVA trials and service evaluations. In order for the core outcome sets to be used widely across interventions, service providers may need to change what they are measuring and how, which will potentially involve additional costs and staff training. However, UK-based family and children's services already collect outcomes to support service planning and delivery, [30–32] so our hope is the CM- and DVA-COS would supplement pre-existing frameworks and support their harmonisation.

Future work needs to consider the international and cross-cultural relevance of the outcomes and their definition, including their application to minoritised groups and families living in varied socioeconomic situations.

Conclusion

Our core outcome sets represent an important first step in developing consistent outcome measurement strategies within and across these two related fields, and advancement towards inclusion of outcomes most important to all stakeholders. Our hope is that researchers will be able to more easily compare outcomes across studies and the evidence base can be synthesised and thus build cumulatively; the ultimate goal being that better quality evidence is available to assist decision makers regarding which services and interventions to support.

Acknowledgements

We wish to thank all the survivors who have taken part at every stage of the study; we are grateful to everyone who used their lived experience to improve this work. Further thanks are due to our survivor involvement groups: VOICES (a survivor-led charity for women who have experienced domestic abuse) and Voices from Care (a charity dedicated to the rights of care experienced young people) in partnership with Rachael Vaughan, Cardiff University. We are also grateful to the health and social care professionals and researchers who have participated and the ongoing advice from our steering group: David and Elisabeth Carney Haworth, Hannah Edwards, Victoria Jepson, Elaine Fulton, Dr Deborah Hodes, Professor Sally Kendall and Professor Carol Rivas. Finally, we thank our independent consensus workshop facilitator, Katherine Cowan, and our workshop counsellor, Dr Alison Gregory, for their input.

Legends

Figure 1 – Study design flowchart

Figure 2 – Stage 1: Outcome identification and longlist compilation flowchart

Figure 3 – Stage 2: E-Delphi consensus process to develop and prioritise core outcomes

Figure 4 – Stage 2: Consensus process participant flowchart

Figure 5 – Stage 2: Selection of outcomes flowchart

Figure 6 – Final core outcome sets

For peer review only - http://bmjopen.bmj.com/site/about/guidelines.xhtml

Author contributions

EH conceived of the original study design, which was refined and developed by EH, CP, RG, GF, ES, and JW. CP, EH and LP designed data collection tools and collected data for the study. CP cleaned and analysed the data and drafted the paper. EH, RG, GF, ES, JW, CP and LP revised the paper.

Funding statement

This study is funded by the National Institute for Health Research (NIHR) Policy Research Programme. Funder reference: PR-PRU-1217-21301; UCL award code: 177763. The views expressed are those of the authors and not necessarily those of the NIHR or the Department of Health and Social Care.

Ethics statement

This study was approved by University College London's Research Ethics Committee (17893/001 & 002).

Competing interests

We have read and understood BMJ policy on declaration of interests and declare that we have no competing interests.

Data sharing statement

Please contact the corresponding author or unit manager cpru.data@ucl.ac.uk with enquiries about the data used in this study.

Supplementary material

Online only supplementary material

References

[1] E. Howarth, T.H. Moore, N.J. Welton, N. Lewis, N. Stanley, H. MacMillan, A. Shaw, M. Hester, P. Bryden, G. Feder, IMPRoving Outcomes for children exposed to domestic ViolencE (IMPROVE): an evidence synthesis, Public Health Res. 4 (2016) 1–342. https://doi.org/10.3310/phr04100.

[2] G. Macdonald, N. Livingstone, J. Hanratty, C. McCartan, R. Cotmore, M. Cary, D. Glaser, S. Byford, N.J. Welton, T. Bosqui, L. Bowes, S. Audrey, G. Mezey, H.L. Fisher, W. Riches, R. Churchill, The effectiveness, acceptability and cost-effectiveness of psychosocial interventions for maltreated children and adolescents: an evidence synthesis, Health Technol. Assess. 20 (2016) 1–508. https://doi.org/10.3310/hta20690.

[3] N.E. Latzman, C. Casanueva, J. Brinton, V.L. Forman-Hoffman, The promotion of well-being among children exposed to intimate partner violence: A systematic review of interventions, Campbell Syst. Rev. 15 (2019). http://dx.doi.org/10.1002/cl2.1049.

[4] R. Gilbert, A. Kemp, J. Thoburn, P. Sidebotham, L. Radford, D. Glaser, H.L. MacMillan, Recognising and responding to child maltreatment, The Lancet. 373 (2009) 167–180. https://doi.org/10.1016/S0140-6736(08)61707-9.

[5] S. Jahanfar, L.M. Howard, N. Medley, Interventions for preventing or reducing domestic violence against pregnant women., Cochrane Database Syst. Rev. (2014) CD009414. https://doi.org/10.1002/14651858.CD009414.pub3.

[6] C. Rivas, J. Ramsay, L. Sadowski, L.L. Davidson, D. Dunne, S. Eldridge, K. Hegarty, A. Taft, G. Feder, Advocacy interventions to reduce or eliminate violence and promote the physical and

For peer review only

For peer review only - http://bmjopen.bmj.com/site/about/guidelines.xhtml

psychosocial well-being of women who experience intimate partner abuse., Cochrane Database Syst. Rev. (2015) CD005043. https://doi.org/10.1002/14651858.CD005043.pub3.

[7] M. Hameed, L. O'Doherty, G. Gilchrist, J. Tirado-Muñoz, A. Taft, P. Chondros, G. Feder, M. Tan, K. Hegarty, Psychological therapies for women who experience intimate partner violence, Cochrane Database Syst. Rev. (2020). https://doi.org/10.1002/14651858.CD013017.pub2.

[8] L.M. Jones, Improving efforts to prevent children's exposure to violence: A handbook to support the evaluation of child maltreatment prevention programmes., World Health Organization, Geneva Switzerland, 2014. https://apps.who.int/iris/rest/bitstreams/646146/retrieve.

[9] E. Howarth, T.H.M. Moore, A.R.G. Shaw, N.J. Welton, G.S. Feder, M. Hester, H.L. MacMillan, N. Stanley, The Effectiveness of Targeted Interventions for Children Exposed to Domestic Violence: Measuring Success in Ways that Matter to Children, Parents and Professionals, Child Abuse Rev. 24 (2015) 297–310. https://doi.org/10.1002/car.2408.

[10] N. Westmarland, L. Kelly, Why Extending Measurements of "Success" in Domestic Violence Perpetrator Programmes Matters for Social Work, Br. J. Soc. Work. 43 (2013) 1092–1110. https://doi.org/10.1093/bjsw/bcs049.

[11] P. Glasziou, I. Chalmers, Research waste is still a scandal—an essay by Paul Glasziou and Iain Chalmers, BMJ. 363 (2018) k4645. https://doi.org/10.1136/bmj.k4645.

[12] Office for National Statistics, Census 2021: Domestic abuse prevalance and trends, Engalnd and Wales, 2021. https://www.ons.gov.uk/peoplepopulationandcommunity/crimeandjustice/articles/domesticabuseprevalenceandtrendsenglandandwales/yearendingmarch2021.

[13] E. Munro, J.S. Taylor, C. Bradbury-Jones, Understanding the Causal Pathways to Child Maltreatment: Implications for Health and Social Care Policy and Practice, Child Abuse Rev. 23 (2014) 61–74. https://doi.org/10.1002/car.2266.

[14] J.L. EDLESON, The Overlap Between Child Maltreatment and Woman Battering, Violence Women. 5 (1999) 134–154. https://doi.org/10.1177/107780129952003.

[15] M. Hester, The Three Planet Model: Towards an Understanding of Contradictions in Approaches to Women and Children's Safety in Contexts of Domestic Violence, Br. J. Soc. Work. 41 (2011) 837–853. https://doi.org/10.1093/bjsw/bcr095.

[16] G. Lessard, P. Alvarez-Lizotte, The exposure of children to intimate partner violence: Potential bridges between two fields in research and psychosocial intervention, Child Abuse Negl. 48 (2015) 29–38. https://doi.org/10.1016/j.chiabu.2015.05.004.

[17] D. Daro, J.L. Edleson, H. Pinderhughes, Finding Common Ground in the Study of Child Maltreatment, Youth Violence, and Adult Domestic Violence, J. Interpers. Violence. 19 (2004) 282–298. https://doi.org/10.1177/0886260503261151.

[18] A.M. Campbell, S.L. Thompson, The emotional maltreatment of children in domestically violent homes: Identifying gaps in education and addressing common misconceptions: The risk of harm to children in domestically violent homes mandates a well-coordinated response, Child Abuse Negl. 48 (2015) 39–49. https://doi.org/10.1016/j.chiabu.2015.08.009.

[19] I. of Medicine, Psychosocial Interventions for Mental and Substance Use Disorders: A Framework for Establishing Evidence-Based Standards, The National Academies Press, Washington, DC, 2015. https://www.nap.edu/catalog/19013/psychosocial-interventions-for-mental-and-substance-use-disorders-a-framework (accessed January 31, 2022).

[20] COMET Initiative | Developing Core Outcome Sets for psycho-social interventions to improve outcomes for children with experience, or at risk, of maltreatment or domestic abuse, (n.d.). https://www.cometinitiative.org/Studies/Details/1576 (accessed January 31, 2022).

[21] E. Howarth, C. Powell, J. Woodman, E. Walker, R. Gilbert, G. Feder, Protocol for developing Core Outcome Sets for evaluation of psychosocial interventions for children and families with experience or at risk of child maltreatment or domestic abuse, BMJ Open. (under review).

For peer review only

[22] J.J. Kirkham, K. Davis, D.G. Altman, J.M. Blazeby, M. Clarke, S. Tunis, P.R. Williamson, Core Outcome Set-STAndards for Development: The COS-STAD recommendations, PLOS Med. 14 (2017) e1002447. https://doi.org/10.1371/journal.pmed.1002447.
[23] J.J. Kirkham, S. Gorst, D.G. Altman, J.M. Blazeby, M. Clarke, D. Devane, E. Gargon, D. Moher, J. Schmitt, P. Tugwell, S. Tunis, P.R. Williamson, Core Outcome Set–STAndards for Reporting: The COS-STAR Statement, PLOS Med. 13 (2016) e1002148. https://doi.org/10.1371/journal.pmed.1002148.
[24] U. Bronfenbrenner, The bioecological model from a life course perspective: Reflections of a participant observer., in: P. Moen, G.H. Elder, K. Lüscher (Eds.), Examining Lives Context Perspect. Ecol. Hum. Dev., American Psychological Association, Washington, 1995: pp. 599–618. https://doi.org/10.1037/10176-017.
[25] C. Firmin, Contextual Safeguarding. An overview of the operational, strategic and conceptual framework., University of Bedfordshire, 2017.
[26] J. Belsky, Etiology of child maltreatment: a developmental-ecological analysis, Psychol. Bull. 114 (1993) 413–434. https://doi.org/10.1037/0033-2909.114.3.413.
[27] L.L. HEISE, Violence Against Women: An Integrated, Ecological Framework, Violence Women. 4 (1998) 262–290. https://doi.org/10.1177/1077801298004003002.
[28] K. Jongsma, J. van Seventer, A. Verwoerd, A. van Rensen, Recommendations from a James Lind Alliance priority setting partnership - a qualitative interview study, Res. Involv. Engagem. 6 (2020) 68. https://doi.org/10.1186/s40900-020-00240-3.
[29] C.A.C. Prinsen, S. Vohra, M.R. Rose, M. Boers, P. Tugwell, M. Clarke, P.R. Williamson, C.B. Terwee, How to select outcome measurement instruments for outcomes included in a “Core Outcome Set” – a practical guideline, Trials. 17 (2016) 449. https://doi.org/10.1186/s13063-016-1555-2.
[30] I.L. Valle, D. Hart, V.S. Pinto, How do we know if children’s social care services make a difference? Development of an outcomes framework, (n.d.) 63.
[31] Cabinet Office, Outcomes Frameworks: a guide for providers and commissioners of youth services, 2014.
[32] C. Montague, R. Pears, N. Hicks, A. Smith, E. Cunningham, S. Bacon, N. Alwan, Developing an outcomes framework for children and young people in Hampshire, England, The Lancet. 392 (2018) S65. https://doi.org/10.1016/S0140-6736(18)32889-7.

For peer review only

For peer review only - http://bmjopen.bmj.com/site/about/guidelines.xhtml

Figure 1 – Study design flowchart

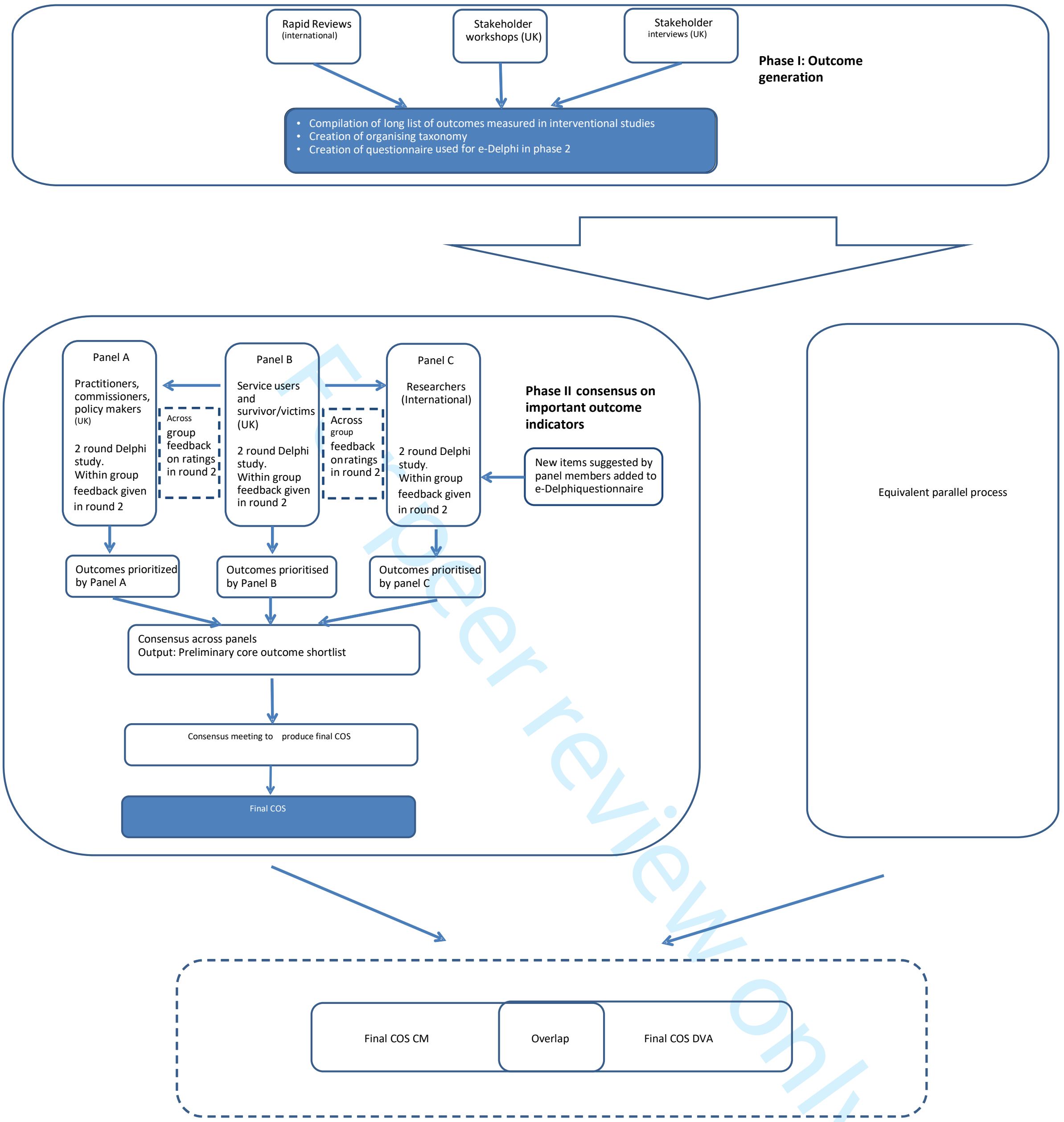

For peer review only

Figure 2 – Stage 1: Outcome identification and longlist compilation flowchart

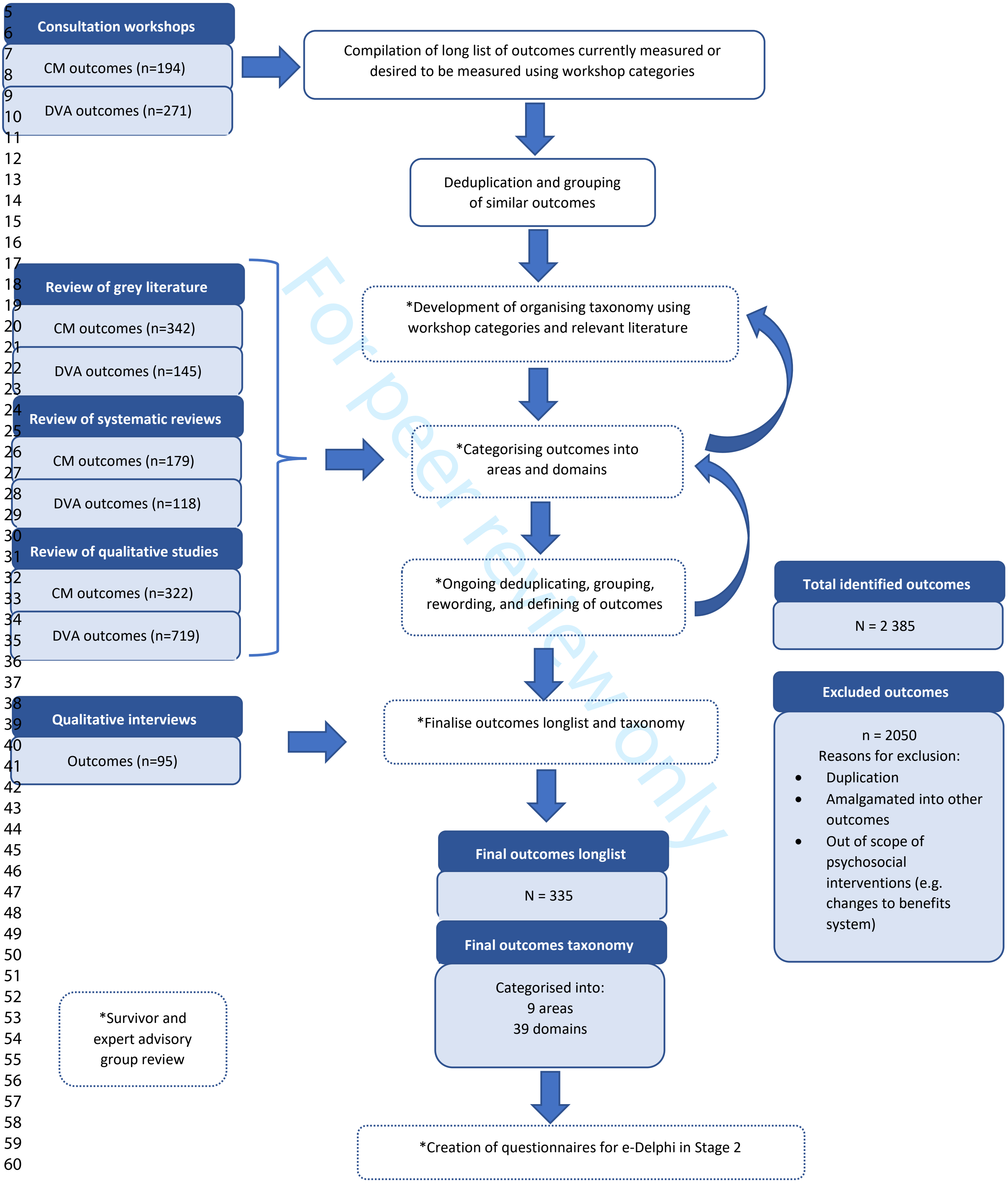

For peer review only

Figure 3 – Stage 2: E-Delphi consensus process to develop and prioritise core outcomes

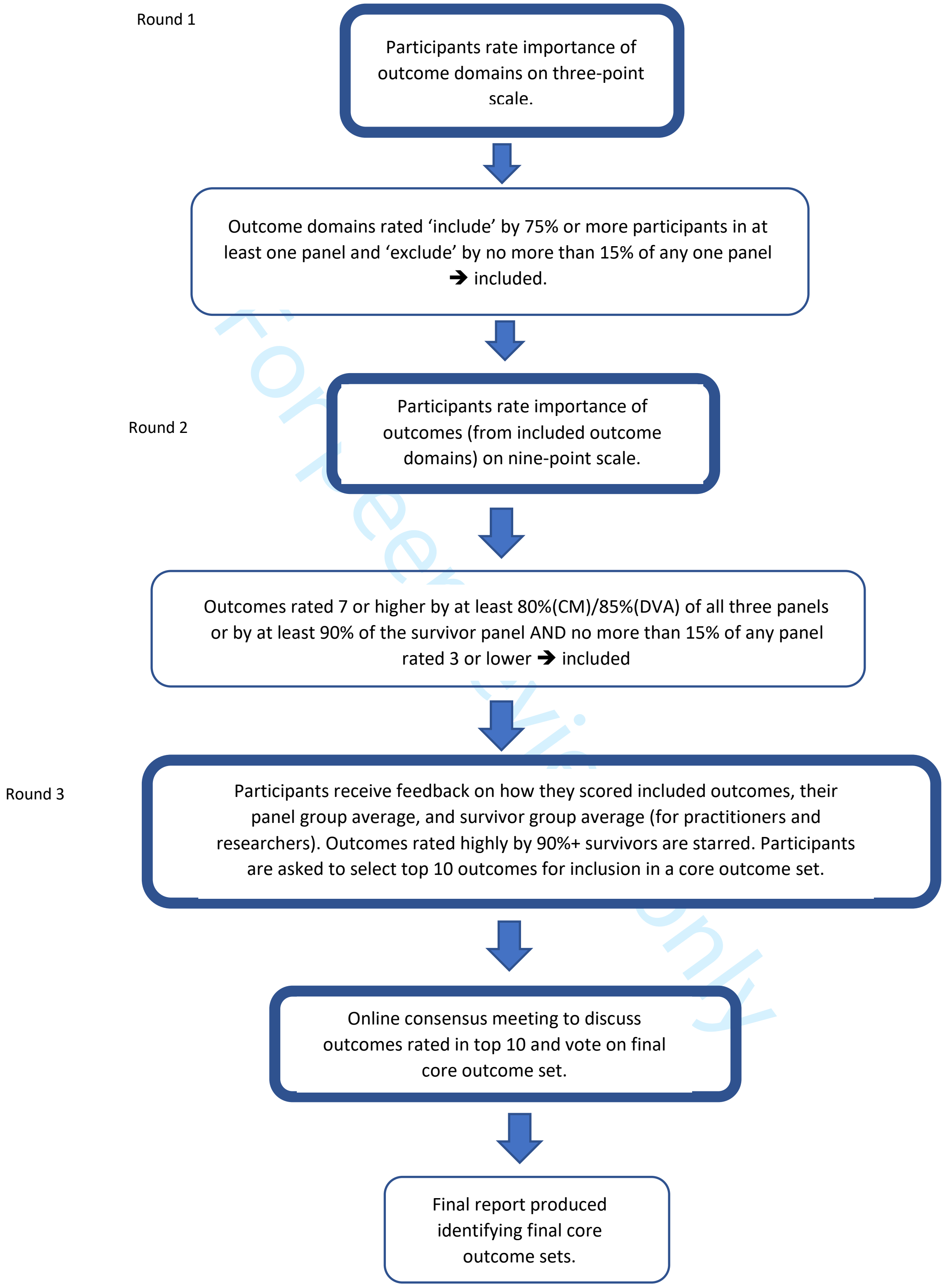

Figure 4 – Stage 2: Consensus process participant flowchart

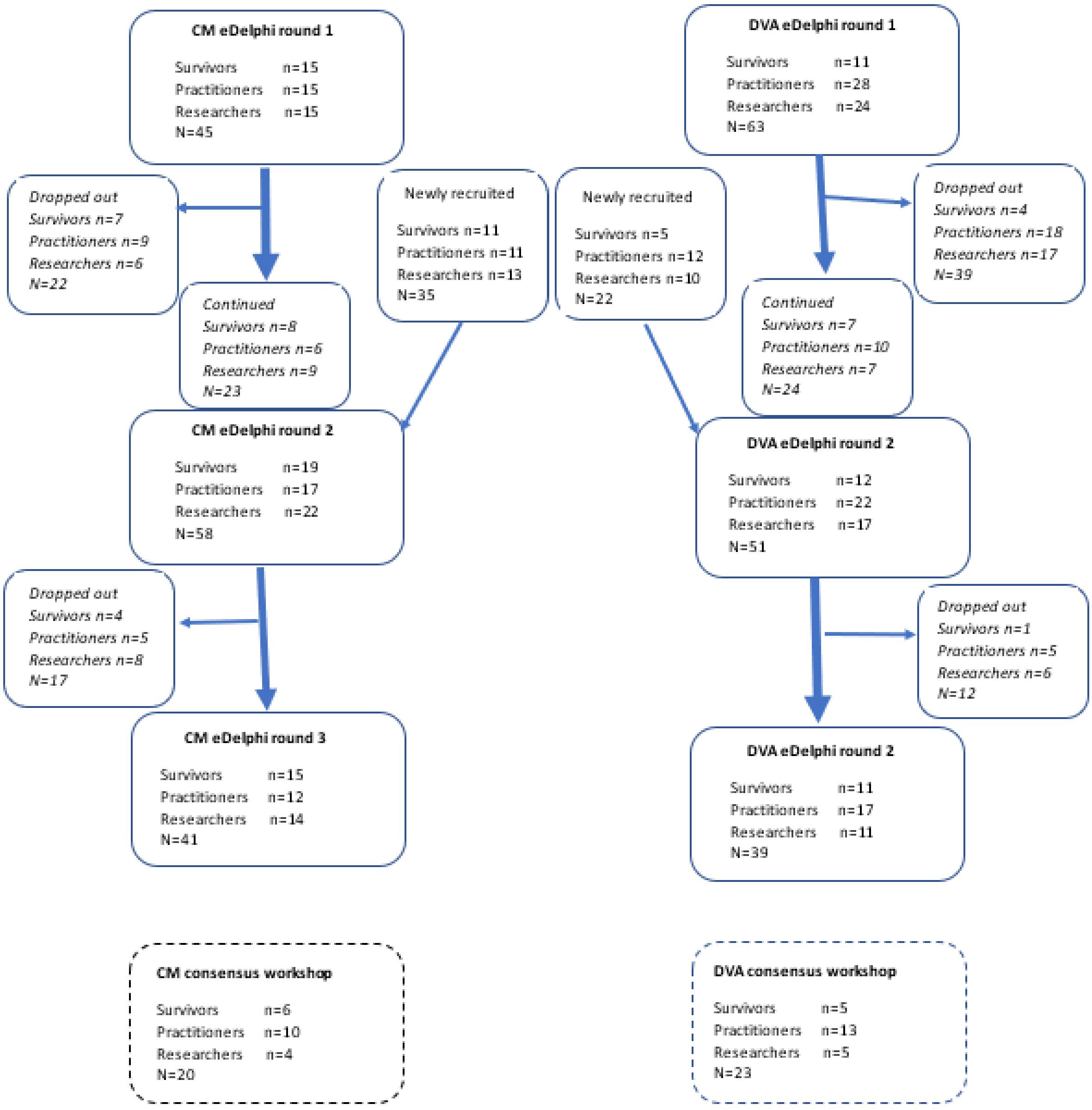

Figure 5 – Stage 2: Selection of outcomes flowchart

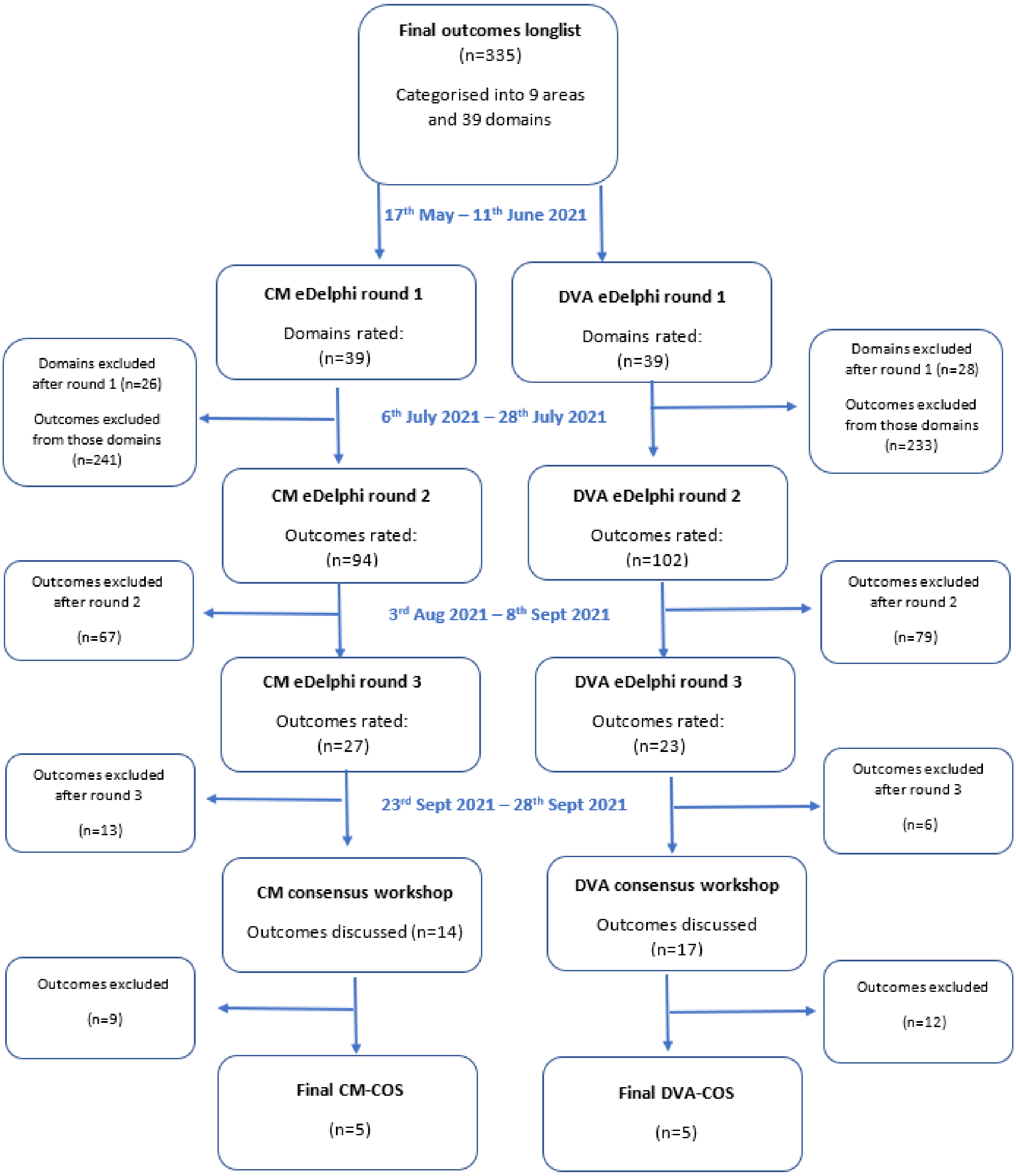

NIHR | Policy Research Unit Children and Families

Child maltreatment

- **Child abuse and neglect**
  includes all harms caused to a child by adults in a position of responsibility

- **Service harms**
  Any retraumatisating effects of the inter-vention

- **Child's trusted relationships**
  Positive relationships a child has with any adults

- **Child emotional health & wellbeing**
  All aspects of emotional and mental health

- **Feelings of safety**
  Includes psychological and physical safety

- **Freedom to go about daily life**
  For example, getting home safely from school

- **Family relationships**
  Quality and type of relationship with birth/foster/adoptive family

- **Caregiver emotional health & wellbeing**
  All aspects of emotional and mental health

Domestic violence

For peer review only - http://bmjopen.bmj.com/site/about/guidelines.xhtml

ucl.ac.uk/children-policy-research

Online only supplementary material

Supplement 1: Sensitivity protocol 2
Supplement 2: Characteristics of participants in the identification (Stage 1) and development (Stage 2) of core outcomes for CM and DVA 3
Supplement 3: Table of inclusion criteria for rapid evidence reviews 6
Supplement 4: Rapid evidence review flowcharts........ 12
Supplement 5: List of websites searched in grey literature review........ 13
Supplement 6: Overview of e-Delphi survey questions........ 15
Round 1: Example question 15
Round 2: Example question 15
Round 3: Example question 15
Supplement 7: List of changes to the protocol 16
Supplement 8: Rating of e-Delphi survey outcomes 17
Round 1 scoring: Child maltreatment and domestic violence and abuse 17
Round 2 and 3 scoring: Child maltreatment 18
Round 2 and 3 scoring: Domestic violence and abuse 22
Supplement 9: DVA consensus workshop consensus statement 26
Supplement 10: Consensus workshop outcome rankings 27
Supplement 11: Summary of discussion about implications of COS for underserved groups 28

For peer review only

Supplement 1: Sensitivity protocol

Before the interview

- Ask for permission to let their support organisation know that they're take part
- Offer for interviewee to bring someone to the interview if they want to
- Ask if there's anything that would help make them feel comfortable
- Offer a choice of venue if possible
- Offer possibility that participants can carry interview out by email
- Recap the aim of the interview/research and who is doing the research

During the interview

- Have a box of tissues but not visibly out
- Ask 'are you happy to talk about this?' and skip to the next question if the answer is no
- Allow pauses during the interview – don't rush to fill silences
- Offer break(s) if necessary or to do the interview in two parts
- Allow interviewees chance to go off-topic

After the interview

- Give a list of support options at the end of the interview
- Give option that interviewee can feedback or give more details
- Researcher to send a text message for follow-up and offer a call
- Explain clearly next steps e.g. referrals

For peer review only - http://bmjopen.bmj.com/site/about/guidelines.xhtml

Supplement 2: Characteristics of participants in the identification (Stage 1) and development (Stage 2) of core outcomes for CM and DVA

STAGE ONE

Month 3

Domestic violence and abuse stakeholder workshop held on 19th June 2019 at Friends House, Euston, London		
Survivors	Practitioners	Researchers
10 survivors attended from:<br>• University of Central Lancashire Survivor Researcher Advisory Group<br>• National Survivor User Network (NSUN)<br>• Violence, Abuse and Mental Health Network (VAMHN)	25 practitioners and policymakers attended from:<br>• Frontline and second tier DVA third sector organisations<br>• Local authority service commissioners, government ministries<br>• Schools and education providers	10 researchers and 14 speakers and facilitators attended from:<br>• UK universities and research groups<br>• International universities and research groups

Month 6

Child maltreatment stakeholder workshop held on 5th September 2019 at Institute of Child Health, University College London, London		
Survivors	Practitioners	Researchers
9 survivors attended from:<br>• University of Central Lancashire Survivor Researcher Advisory Group<br>• National Survivor User Network (NSUN)<br>• Violence, Abuse and Mental Health Network (VAMHN)	16 practitioners and policymakers attended from:<br>• Frontline and second tier CM third sector organisations<br>• Local authorities, government ministries<br>• Health service providers and commissioners<br>• Social care professionals and associated organisations	6 researchers and 10 speakers and facilitators attended from:<br>• UK universities and research groups

Months 17 to 21

Qualitative survivor interviews held online from August to December 2020 (N=10)	
Demographics	n (%)
Age	
18 to 35 years	2 (20)
36 to 45 years	2 (20)
46+ years	5 (50)
Unknown	1 (20)
Sex	
Female	8 (80)
Male	2 (20)
Ethnicity	
Asian-European	2 (20)
Black British/Caribbean	2 (20)
White British/UK	5 (50)
Other	1 (10)
Type of abuse	
Child maltreatment only	0 (0)
Domestic violence only	5 (50)
Both	5 (50)

For peer review only - http://bmjopen.bmj.com/site/about/guidelines.xhtml

STAGE TWO

Months 26 to 30

E-Delphi surveys held online from May to Sept 2021 (N=173)<br>*CM and DVA participants amalgamated for anonymity*	
Demographics	n (%)
Panel	
Survivors	42 (25)
Practitioners	66 (39)
Researchers	62 (36)
Age	
18 to 25 years	2 (1)
26 to 35 years	21 (12)
36 to 45 years	49 (29)
46 to 55 years	42(25)
56 to 65 years	44 (26)
66+ years	12 (7)
Sex / gender	
Female	146 (86)
Male	22 (13)
*Non-binary / third gender / Trans**	2 (2)
Ethnicity	
Asian or Asian British (Indian, Pakistani, Bangladeshi, Chinese, Any other Asian background)	10 (6)
Black / African / Caribbean / Black British	6 (4)
Mixed / multiple ethnic groups	16 (9)
White (English, Welsh, Scottish, Northern Irish, British, Irish, Gypsy or Irish Traveller, Any other White background)	137 (81)
Type of abuse (survivors only)	
Child maltreatment only	8 (19)
Domestic violence only	9 (21)
Both	25 (60)

Month 30

Child maltreatment consensus workshop held online on 23rd September 2021		
Survivors	Practitioners	Researchers
6 survivors attended from:<br>• University of Central Lancashire Survivor Researcher Advisory Group<br>• National Survivor User Network (NSUN)<br>• Violence, Abuse and Mental Health Network (VAMHN)<br>• E-Delphi survey participation	*10 practitioners and policymakers attended from:*<br>• Frontline and second tier CM third sector organisations<br>• Local authorities, government ministries<br>• Health service providers and commissioners<br>• Social care professionals and associated organisations	*4 researchers and 4 facilitators attended from:*<br>• UK universities and research groups<br>• International universities and research groups

For peer review only - http://bmjopen.bmj.com/site/about/guidelines.xhtml

Month 30

Domestic violence consensus workshop held online on 28th September 2021		
Survivors	Practitioners	Researchers
5 survivors attended from:<br>• University of Central Lancashire Survivor Researcher Advisory Group<br>• National Survivor User Network (NSUN)<br>• Violence, Abuse and Mental Health Network (VAMHN)<br>• E-Delphi survey participation	*13 practitioners and policymakers attended from:*<br>• Frontline and second tier DVA third sector organisations<br>• Local authorities, government ministries<br>• Health service providers and commissioners<br>• Social care professionals and associated organisation<br>• Police	*5 researchers and 4 facilitators attended from:*<br>• UK universities and research groups

For peer review only

For peer review only - http://bmjopen.bmj.com/site/about/guidelines.xhtml

Supplement 3: Table of inclusion criteria for rapid evidence reviews

Review	Criteria	Inclusion criteria	Exclusion criteria
Review of systematic review	Study type	Peer-reviewed systematic reviews of controlled or quasi experimental comparator intervention studies: with or without randomisation.<br><br>The DARE criteria for SRs are at least 4 of the following: reporting of inclusion/exclusion criteria; adequate search; synthesis of included studies; quality assessment of studies; sufficient detail presented (CRD, 1995). For the purposes of this review, SRs will be included if they use an electronic database and have a structured search strategy.<br><br>Published since 2014.<br><br>No restrictions by country. English language only.<br><br>Individual studies must include DVA/CM in one of the following ways:<br><br>o Entry to the intervention is determined by experience, perpetration or identified as at risk of DVA/CM. (Identification of risk is by researchers, practitioners, or participants thus we do not have a definition)<br><br>o Subgroup analysis is carried out of participants who have experienced (or are considered to be at risk of) DVA/CM<br><br>o DVA/CM is measured as an exposure (this could be retro or prospectively reported)	Non peer-reviewed studies<br><br>Qualitative studies<br><br>General literature reviews<br><br>Protocols<br><br>Case reports<br><br>Cross-sectional studies<br><br>General discussion papers<br><br>Letters<br><br>Commentaries<br><br>Book chapters<br><br>Conference papers<br><br>Theses and dissertations.
	Population	Children or families with children at risk of experiencing or experiencing DVA/CM. This includes unborn children, children (aged 0 to 18 years), designated as victim or witness.<br><br>For DVA any adult family members who have a parenting role (Early Intervention Foundation, 2014), whether designated as perpetrator, victim, witness, or household member.	

For peer review only

For peer review only - http://bmjopen.bmj.com/site/about/guidelines.xhtml

	For CM any adult family members who have a caring role, whether designated as maltreating parent, witness, or household member.<br><br>These adults and children could either be the primary study population of interest or form a subgroup in a wider study population.	
Intervention	Any interventions or services where:<br><br>Experience of or increased risk of experiencing DVA/CM is a criterion for being offered the service<br><br>OR<br><br>DVA/CM is measured as an exposure or outcome of interest<br><br>AND<br><br>At least one child or family-level outcome is measured. Family-level outcomes do not need to be explicitly labelled as 'family' level; we will make a judgement. However, they include any outcome that affects the family/household unit. For example, worklessness in study where at least some participants are reported to be parents would be included.<br><br>Studies must include evaluation of a defined activity/programme and evaluation of a hypothesised effect.<br><br>Interventions may be delivered to any family member(s) as an individual or in a group. Any duration of intervention will be included. Any setting will be considered.	Universal interventions that do not specifically target children and families at risk of<br><br>DVA/CM; targeted interventions that do not measure any child or family level outcomes e.g. perpetrator programmes that focus solely on attitudinal change; DVA (only) interventions focused solely on elder abuse, sibling abuse or child perpetration of domestic violence where participants have not been identified as exposed to DVA.
Comparator	Any control or comparison group/period with participants receiving no care, treatment as usual or any other treatment	

For peer review only

For peer review only - http://bmjopen.bmj.com/site/about/guidelines.xhtml

	Outcome	Any child outcome related to i) the child's experience of adversity ii) child functioning, including risky behaviours.<br><br>Any outcomes related to the quality of the caregiving environment (e.g. parenting, maternal depression, stressful life events, maternal psychological distress, parental substance misuse).<br><br>Any outcomes related to material deprivation e.g. low income, economic hardship, or stress (including perceived), social capital, hunger, food poverty, housing instability.<br><br>Any other outcome judged to relate to children or families by the research team.<br><br>Outcomes can be reported by professionals, child, parent or other family member and they can be retrospective or prospective.<br><br>Outcomes can be end points, surrogate markers for end points or intermediate outcomes.<br><br>No minimum or maximum follow-up is required.	
	Context	Studies from any country in any setting	
Review of qualitative studies	Study type	Primary qualitative (i.e. analysis of interviews, focus groups or other verbal analysis which is not quantified) intervention studies either as a standalone study or a discrete component of mixed method studies.<br><br>Direct and sufficient verbatim text from participants for analysis (i.e. more than two lines) c.f. Arai et al. (2019).<br><br>Published since October 2015 (DVA) and July 2014 (CM) to build on Howarth et al. (2016) and Macdonald et al. (2016).<br><br>No restrictions by country. English language only.<br><br>Individual studies must include DVA/CM in one of the following ways:	Non peer-reviewed studies<br><br>Surveys or quantitative studies with descriptive free text only<br><br>General literature reviews<br><br>Case reports<br><br>General discussion papers<br><br>Letters<br><br>Commentaries

For peer review only - http://bmjopen.bmj.com/site/about/guidelines.xhtml

		o Participation in the study is determined by experience, perpetration or specifically identified as at risk of DVA/CM. Participants may have received an intervention or may be discussing the impact of DVA/CM and their desired outcomes for the future. (To ensure we are not limited by outcomes defined by current interventions).<br><br>OR<br><br>o Stakeholders involved in developing and/or delivering interventions to children/families experiencing DVA/CM (c.f. Howarth et al, 2016, p.52), or stakeholder discussion of outcomes that are sought either in relation to an intervention or the future in general.	Editorials<br><br>Book chapters<br><br>Conference papers<br><br>Theses and dissertations
	Population	Any adult or child stakeholders relevant to DVA/CM. This could be because of experience, perpetration, identified as at risk, delivering, commissioning, or intending to deliver services.	
	Phenomenon of interest	DVA/CM	
	Design	Any qualitative approach to data collection and analysis (e.g. interviews, focus groups).	
	Evaluation	Perspectives of experienced or anticipated benefits or harms of interventions, and/or desired outcomes in general related to DVA/CM.	
Review of grey literature	Literature	Any national or regional policy or practice document that reports on DVA/CM-relevant services or outcomes (e.g. measurement/theory).<br><br>Participation in the service is determined by experience, perpetration or identified as at risk of DVA/CM. (Identification of risk is by practitioners or participants thus we do not have a definition).	Publication in academic journals<br><br>Book chapters<br><br>Conference papers<br><br>Theses and dissertations.

For peer review only - http://bmjopen.bmj.com/site/about/guidelines.xhtml

	Published since 2016 to build on Howarth et al. (2016) and Macdonald et al. (2016).<br><br>England-based only. English language only.	
Population	Children or families with children at risk of experiencing, or experiencing DVA/CM. This includes unborn children, children (aged 0 to 18 years), designated as victim or witness.<br><br>For DVA any adult family members who have a caring or parenting role (Early Intervention Foundation, 2014), whether designated as perpetrator, victim, witness, or household member.<br><br>For CM any adult family members who have a caring role, whether designated as perpetrator, witness, or household member.	
Service	Experience of or increased risk of experiencing DVA/CM is a criterion for being offered the service/intervention.<br><br>Services/interventions may be delivered to any family member(s) as an individual or in a group.<br><br>Any duration of service/intervention will be included.<br><br>Any setting will be considered.<br><br>OR<br><br>Any evaluative work or outcomes framework where at least one child or family-level outcome is evaluated/discussed. Family-level outcomes do not need to be explicitly labelled as 'family' level; we will make a judgement. However, they include any outcome that affects the family/household unit. For example, worklessness in study where at least some participants are reported to be parents would be included<br><br>.	Universal services/interventions that do not specifically target children and families at risk of DVA/CM.<br><br>Targeted services/interventions that do not measure any child or family level outcomes e.g. perpetrator programmes that focus solely on attitudinal change.<br><br>DVA (only) services/interventions focused solely on elder abuse, sibling abuse or child perpetration of domestic violence, where participants have not been identified as exposed to DVA (i.e. perpetration of abuse by a child could feasibly be an outcome associated with exposure).

Outcome	Any family or child-level outcome measured or evaluated or discussed in any way.<br><br>Intermediate outcomes that could feasibly represent preconditions needed to reach distal/final outcomes (including those relating to the process of service delivery) will be included, along with final/distal outcomes.

For peer review only

For peer review only - http://bmjopen.bmj.com/site/about/guidelines.xhtml

Supplement 4: Rapid evidence review flowcharts

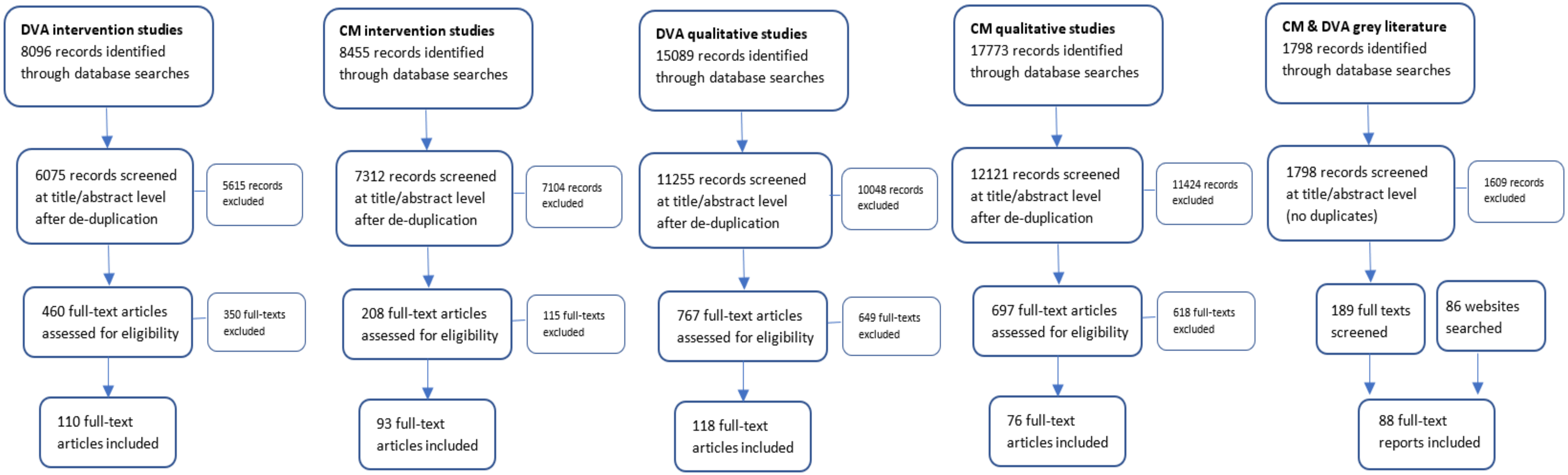

For peer review only - http://bmjopen.bmj.com/site/about/guidelines.xhtml

Supplement 5: List of websites searched in grey literature review

Organisation	Website	Date searched
Women's Aid	https://www.womensaid.org.uk/	07/02/20 to 11/02/20
Refuge	www.refuge.org.uk	12/02/2020
Respect	http://respect.uk.net/	12/02/2020
Safe Lives	http://www.safelives.org.uk/	12/02/2020
Voices	http://www.voicescharity.org/	14/02/2020
AVA	https://avaproject.org.uk/	14/02/2020
Standing Together	http://www.standingtogether.org.uk/	14/02/2020
Imkaan	https://www.imkaan.org.uk/	14/02/2020
The Stefanou Foundation	https://www.stefanoufoundation.org/	14/02/2020
Women's Trust	https://womanstrust.org.uk/	14/02/2020
Hestia	https://www.hestia.org/	14/02/2020
DVIP	https://dvip.org/	14/02/2020
Nia	http://www.niaendingviolence.org.uk/	14/02/2020
The Havens	https://www.thehavens.org.uk/	14/02/2020
ManKind Initiative	https://www.mankind.org.uk/	14/02/2020
Everyman Project	http://www.everymanproject.co.uk/	14/02/2020
NCDV (National Centre for Domestic Violence)	https://www.ncdv.org.uk/	14/02/2020
Galop	http://www.galop.org.uk/	14/02/2020
LAWA (Latin American Women's Aid)	http://lawadv.org.uk/en/	14/02/2020
IDAS	https://www.idas.org.uk/	17/02/2020
Advance	http://advancecharity.org.uk/	17/02/2020
Your Sanctuary	https://www.yoursanctuary.org.uk/	17/02/2020
Advocacy After Fatal Domestic Abuse (AAFDA)	https://aafda.org.uk/	17/02/2020
Aurora New Dawn	https://www.aurorand.org.uk/	17/02/2020
My Sister's Place	https://www.mysistersplace.org.uk/	17/02/2020
Surviving Economic Abuse	https://survivingeconomicabuse.org/	17/02/2020
Latin American Women's Rights Service	http://www.lawrs.org.uk/	17/02/2020
Step Up Migrant Women	https://stepupmigrantwomen.org/	17/02/2020
Southall Black Sisters	https://southallblacksisters.org.uk/	17/02/2020
End Violence Against Women	https://www.endviolenceagainstwomen.org.uk/	17/02/2020
Welsh Women's Aid	https://www.welshwomensaid.org.uk/	17/02 2020
Agenda	https://weareagenda.org/	17/02/2020
Rights of women	https://rightsofwomen.org.uk/	17/02/2020
Solace	https://www.solacewomensaid.org/	17/02/2020
Domestic Abuse Housing Alliance	https://www.dahalliance.org.uk/	18/02/2020
Reducing the Risk of Domestic Abuse	https://www.reducingtherisk.org.uk/cms/	18/02/2020
Drive	http://driveproject.org.uk/	18/02/2020
Centre of expertise on child sexual abuse	https://www.csacentre.org.uk/	18/02/2020
FDAC (Family Drug and Alcohol Courts)	https://fdac.org.uk/	18/02/2020
SCIE (Social Care Institute for Excellence)	https://www.scie.org.uk/	18/02/2020
The Survivors Trust	https://www.thesurvivorstrust.org/	19/02/2020
Plan International UK	https://plan-uk.org/	19/02/2020
Victim Support	https://www.victimsupport.org.uk/	19/02/2020
Rape Crisis England & Wales	https://rapecrisis.org.uk/	19/02/2020
IKWRO (Iranian and Kurdish Women's Rights Organisation)	http://ikwro.org.uk/	19/02/2020
Karma Nirvana	https://karmanirvana.org.uk/	19/02/2020
Forward	https://www.forwarduk.org.uk/	19/02/2020
SignHealth	https://www.signhealth.org.uk/	19/02/2020
NSPCC	https://www.nspcc.org.uk/	19/02/2020
NAPAC (The National Association for People Abused in Childhood)	https://napac.org.uk/	19/02/2020
Barnardo's	https://www.barnardos.org.uk/	19/02/2020
Early Intervention Foundation	https://www.eif.org.uk/	19/02/2020
NatCen	http://natcen.ac.uk/	19/02/2020
RCGP (Royal College of General Practitioners)	https://www.rcgp.org.uk/	19/02/2020
RCN (Royal College of Nursing)	https://www.rcn.org.uk/	19/02/2020

For peer review only

For peer review only - http://bmjopen.bmj.com/site/about/guidelines.xhtml

Coercive Control	https://coercivecontrol.ripfa.org.uk/	21/02/2020
Domestic Violence UK	https://domesticviolenceuk.org/	21/02/2020
Live Fear Free Helpline	https://gov.wales/live-fear-free	21/02/2020
Living Without Abuse	http://www.lwa.org.uk/	21/02/2020
Hidden Hurt	http://www.hiddenhurt.co.uk/	21/02/2020
RCM (Royal College of Midwives)	https://www.rcm.org.uk/	21/02/2020
NICE (National Institute for Health and Care Excellence)	https://www.nice.org.uk/	21/02/2020
Broken Rainbow UK	https://www.brokenrainbow.org.uk/	21/02/2020
BPS (The British Psychological Society)	https://www.bps.org.uk/	21/02/2020
iHV (Institute of Health Visiting)	https://ihv.org.uk/	21/02/2020
Working together to safeguard children	https://www.gov.uk/government/publications/working-together-to-safeguard-children--2	21/02/2020
WHO	https://www.who.int/	24/02/2020
Public Health England	https://www.gov.uk/government/organisations/public-health-england	24/02/2020
Public Health Scotland	https://publichealthreform.scot/public-health-scotland	24/02/2020
Public Health Wales	https://phw.nhs.wales/	24/02/2020
Public Health Agency Northern Ireland	https://www.publichealth.hscni.net/	26/02/2020
Family Support NI	https://www.familysupportni.gov.uk/	26/02/2020
UNICEF	https://www.unicef.org.uk/	26/02/2020
The National Lottery Community Fund	https://www.tnlcommunityfund.org.uk/	26/02/2020
Comic Relief	https://www.comicrelief.com/	26/02/2020
The Childhood Trust	https://www.childhoodtrust.org.uk/	26/02/2020
College of Policing UK	https://www.college.police.uk/Pages/Home.aspx	26/02/2020
College What Works	https://whatworks.college.police.uk/Pages/default.aspx	26/02/2020
What Works for Children's Social Care	https://whatworks-csc.org.uk/	26/02/2020
Nuffield Foundation	https://www.nuffieldfoundation.org/	27/02/2020
Children's Commissioner for England	https://www.childrenscommissioner.gov.uk/	05/03/2020
Children's Commissioner for Wales	https://www.childcomwales.org.uk/	06/03/2020
Children and Young People's Commissioner Scotland	https://www.cypcs.org.uk/	09/03/2020
Research in Practice	https://www.researchinpractice.org.uk/children/	09/03/2020
The Joseph Rowntree Foundation	https://www.jrf.org.uk/	11/03/2020
British Medical Association (BMA)	https://www.bma.org.uk	18/03/2020

For peer review only - http://bmjopen.bmj.com/site/about/guidelines.xhtml

Supplement 6: Overview of e-Delphi survey questions

Round 1: Example question

Q1

Below are the domains in **child health & wellbeing**. These relate to children at any age - at this stage we are not specifying any details, just the broad ideas.

To see further details about each domain hover your mouse/cursor over the item. Then drag and drop into the three categories below, depending on how important it is that they are included in a core outcome set for **child abuse and neglect interventions**. Please try and use all 3 categories.

	Definitely include	Include if possible	Can be dropped
Child physical health			
Child wellbeing			
Child mental distress			
Child sense of self			
Child self-care & coping			
Child social relations & social wellbeing			
Child long term outcomes			

Q1a

Please add any comments on child health & wellbeing in general or any of the domains here:

Round 2: Example question

Q6.1

Below are outcomes in the domain **family function**. Please decide how important you think each outcome is for judging how helpful interventions are for children and families who have experienced child abuse and neglect and rate each one on a nine-point scale before moving on to the next domain.

	9 - Extremely unimportant	8 - Very unimportant	7 - Unimportant	6 - Maybe unimportant	5 - Unsure unimportant or important	4 - Maybe important	3 - Important	2 - Very important	1 - Extremely important
**Family functioning and stability**: includes stable carers	○	○	○	○	○	○	○	○	○
**Family relationships**: includes overall family relationships and functioning, quality and type of relationships, feeling closer as a family, family conflict resolution, feeling closer to children, changes after leaving abusive partner; sibling relationships including after separation; child relationship with birth and foster/adoptive families	○	○	○	○	○	○	○	○	○
**Family emotional environment**: includes emotional security, protective environment, secrecy at home	○	○	○	○	○	○	○	○	○
	9 - Extremely unimportant	8 - Very unimportant	7 - Unimportant	6 - Maybe unimportant	5 - Unsure unimportant or important	4 - Maybe important	3 - Important	2 - Very important	1 - Extremely important
**Family communication**: includes with each other and others outside the family	○	○	○	○	○	○	○	○	○
**Missing episodes** i.e., child missing from home	○	○	○	○	○	○	○	○	○

Q6.2

Please add any comments here about outcomes related to **family function**

Round 3: Example question

Please select up to 10 outcomes (out of 27 in total) that you would like to include in a core outcome set. We recommend that you scroll through the list first and then make your choices.

Please consider that the aim is for this core outcome set to be used across different types of interventions and services. **The outcomes that at least 90% of survivors found important are starred so you can keep the survivor group perspective in mind** because the current focus is mostly on researcher or practitioner priorities. The outcomes are presented in a random order to reduce bias along with the average scores for the survivor group and your individual score so you see how your score compared to other groups. This might inform your decision about what to include. If you missed any questions last time, your previous score will be blank.

As a reminder the scale was:	9 - Extremely unimportant	8 - Very unimportant	7 - Unimportant	6 - Maybe unimportant	5 - Unsure unimportant or important	4 - Maybe important	3 - Important	2 - Very important	1 - Extremely important	0 Selected

☐ **Child stress*** includes signs of stress, i.e., cortisol and other biological measures, subjective feelings of stress

Your previous score = ${e://Field/1A3_r2}
Survivor group average score = 2 - very important

Supplement 7: List of changes to the protocol

- Interviews and consensus workshops were held online to meet national Covid-19 advice and to include participants who were self-isolating.
- We adapted the survey design in response to the longer than expected list of outcomes and to reduce participant burden. In the first round we asked participants to rate outcome domains on a three-point scale, rather than outcomes. The second round then involved a survey of just those outcomes whose domains were selected across the three groups (see methods for details).
- Although the e-Delphi was planned to take place before UK school holidays, Covid-19 delays to the UCL research registration process meant the survey was delayed and took place during the UK summer break. To mitigate against anticipated attrition, new participants were invited to join the survey in the second round.
- Unexpectedly we found extremely high levels of consensus at each stage, thus we increased the consensus levels for outcome inclusion. Staying at the lower levels would have involved including all outcomes. Similarly, we adapted the final round (after participants received feedback) to selecting ten outcomes, rather than re-rating, because all the included outcomes had been rated so highly.

For peer review only

For peer review only - http://bmjopen.bmj.com/site/about/guidelines.xhtml

Supplement 8: Rating of e-Delphi survey outcomes

Round 1 scoring: Child maltreatment and domestic violence and abuse

		CM			DVA		
		Survivor	Practitioner	Researcher	Survivor	Practitioner	Researcher
Area	Domain	Median (IQR)	Median (IQR)	Median (IQR)	Median (IQR)	Median (IQR)	Median (IQR)
**Child health & wellbeing**							
	Child physical health	3 (1)	3 (0.5)	3 (0)	3 (0.5)	2 (0.5)	2 (1)
	Child wellbeing	3 (1)	3 (0)	3 (1)	3 (0)	3 (0)	3 (1)
	Child mental distress	3 (0)	3 (0.5)	3 (0)	3 (0)	3 (0.5)	3 (0.5)
	Child sense of self	2 (1.5)	2 (1.5)	2 (1)	3 (1)	2 (0)	2 (1)
	Child self-care & coping	2 (1.5)	2 (0)	2 (1)	2 (1)	2 (0)	2 (1)
	Child social relations & social wellbeing	2 (1.5)	2 (2)	3 (1)	2 (1)	3 (1)	2 (1)
	Child long term outcomes	2 (1.5)	2 (1)	3 (1)	2 (1.5)	2 (0)	2 (1)
**Caregiver health & wellbeing**							
	Caregiver physical health	2 (0.5)	2 (1.5)	2 (1)	3 (1)	2 (0.5)	2 (1)
	Caregiver wellbeing	2 (1)	3 (1)	3 (1)	3 (0.5)	3 (1)	3 (1)
	Caregiver mental distress	3 (1)	3 (1)	3 (0)	3 (0)	3 (0.5)	3 (0.5)
	Caregiver sense of self	1 (1)	2 (1)	2 (1)	2 (1)	2 (0)	2 (1)
	Caregiver self-care & coping	2 (1.25)	2 (0.5)	3 (1)	2 (1)	3 (1)	2 (1)
	Caregiver social relations	2 (2)	2 (1.5)	2 (1)	2 (0.5)	2 (0)	2 (1)
**Caregiver relationships & parenting**							
	Caregiver-child relationship	3 (0)	3 (0)	3 (0)	3 (0)	3 (0)	3 (0)
	Relationship between caregivers	2 (1.5)	2 (1)	2 (1)	2 (1)	2.5 (0.5)	2 (1)
	Parent	3 (1)	2 (1)	3 (0)	2 (1)	3 (1)	3 (1)
	Parental thoughts, feelings, knowledge & understanding of the parental role	2 (0.75)	2 (1)	2 (1)	3 (1)	2 (0)	2 (0.5)
**Home environment & household**							
	Family function	2 (1)	3 (0)	3 (0)	3 (1)	3 (0.5)	3 (0.5)
	Home environment & housing	3 (1)	2 (1)	3 (0.5)	2 (1)	2 (0)	2 (2)
	Family income & employment	2 (2)	2 (1)	2 (1)	2 (1)	2 (1)	2 (1)
**Social support & peer relationships**							
	Child's relationships & social support	3 (0)	3 (0)	3 (1)	3 (1)	3 (1)	3 (1)
	Family relationships & social support	2 (1)	2 (1)	3 (0)	3 (1)	3 (1)	2.5 (1)
**Community resources & institutions**							
	Education/training, delivery & experience (for child/young person)	3 (1)	2 (1)	2 (1)	3 (1)	3 (1)	2 (1)
	Social services delivery & experience	3 (1)	3 (1)	3 (1)	3 (1.5)	3 (0)	3 (1)
	Access, use and knowledge of community resources	2 (1.5)	2 (1)	3 (0.5)	2 (1)	2 (0)	2 (1)
	Experience of community resources	2 (1.75)	2 (1)	2 (1)	2 (0.5)	2 (1)	2 (1)
**Safety, feelings & knowledge related to violence and abuse**							
	Safety	3 (1.5)	3 (1)	3 (0)	3 (0)	3 (0)	3 (0.5)
	Child's contact with harmful people	3 (1.5)	3 (0.5)	3 (0)	3 (0)	3 (1)	2 (1.5)
	Child's thoughts & knowledge about their experiences of CM or DVA	3 (0.5)	3 (1)	3 (1)	3 (1)	3 (0)	2.5 (1)
	Non harming parent's thoughts & feelings about their experience of DVA or maltreatment of their child	2 (0.5)	2 (1)	2 (1)	2 (1)	2 (0)	2 (1)
	Perpetrator/harmful parent perception of responsibility & understanding of violence and abuse	2 (0)	2 (1)	2 (1)	3 (1.5)	2 (0)	2 (0.75)
	Responding after violence & abuse	3 (1)	2 (1)	3 (1)	2 (1)	3 (1)	2 (1)
**Violence, abuse and maltreatment**							
	Child maltreatment	3 (0)	3 (0)	3 (0)	3 (0)	3 (0)	3 (0)
	Child specific exposure to DVA	3 (1)	3 (0.5)	3 (0)	3 (0.5)	3 (0)	3 (0.5)
	Domestic violence and abuse between	2 (1.5)	2 (1)	2 (1)	3 (0)	3 (0.5)	2 (1)
	General violence & abuse	2 (1)	2 (1.5)	2 (1)	2 (0.5)	2 (0)	1.5 (1)
**Intervention outcomes**							
	Process of intervention delivery	2 (0.75)	2 (1)	3 (1)	2 (1)	3 (1)	2 (1)
	Practitioner approach and	2 (1.75)	2 (0.5)	2 (1)	2 (0.5)	2 (0)	2 (1)
	Intervention adverse effects	3 (1)	2 (1)	3 (0)	3 (0.5)	3 (1)	3 (0.5)
	Client experience and evaluation	3 (0.5)	3 (0.5)	3 (1)	2 (1)	3 (1)	3 (1)

For peer review only - http://bmjopen.bmj.com/site/about/guidelines.xhtml

Round 2 and 3 scoring: Child maltreatment

			Round 2						Round 3							
			Survivor		Practitioner		Researcher		Survivor		Practitioner		Researcher			
Area	Domain	Outcome	Median	(IQR)	Median	(IQR)	Median	(IQR)	Score	(%)	Score	(%)	Score	(%)	TOTAL	RANK
**Child health & wellbeing**																
	Child physical health															
		Child global physical health	3	(2)	2	(2)	2	(2.25)								
		Child sleep	2	(1.5)	3	(2)	2.5	(1.25)								
		Child stress	2	(1)	3	(1.75)	2	(2)	2	(13)	3	(25)	6	(43)	11	14
		Child birth outcomes	3.5	(2)	3	(1)	2	(2)								
		Child pain	2.5	(1)	3	(2)	4	(2)								
		Child physical wellbeing	3	(2)	2.5	(2)	3	(1.25)								
		Child sexual health	3	(2)	2.5	(1)	3	(2)								
	Child wellbeing															
		Child behaviour	2	(2)	2	(2)	1	(1)								
		Child aggression	3	(2)	2	(1)	2	(1)								
		Child development	3	(2)	3	(2)	2	(1.5)								
		Child global functioning	3	(2)	3	(2.5)	2	(2)								
		Child speech and language	3	(1)	3	(2)	3	(1)								
		Child enuresis or soiling related to behaviour	3	(1)	3	(2)	3	(1.5)								
		Child emotional health & wellbeing	2	(1.5)	2	(1)	1	(1)	7	(47)	5	(42)	8	(57)	20	6=
		Child emotional understanding	2	(1)	3	(2)	3	(2)								
		Child ability to concentrate	3	(1.5)	3	(1.5)	3	(2)								
		Child executive functioning	3	(1)	2	(1.75)	3	(1)								
		Child anger	2	(2)	3	(0.5)	4	(1)	3	(20)	1	(8)	3	(21)	7	17
		Child sense of calm	2	(1.5)	3	(1)	2	(1)	1	(7)	0	(0)	0	(0)	1	21
		Child sense of panic	2	(1)	3	(1.5)	3	(2.75)								
		Child happiness	2	(1.5)	3	(1)	2	(1)	3	(20)	1	(8)	2	(14)	6	18
		Child perception of recovery from trauma	2	(0.5)	2	(0.5)	2	(1)	1	(7)	4	(33)	5	(36)	10	15
		Child awareness of own body	3	(2)	3	(2)	2	(1.75)								
		Child overall wellbeing	3	(2)	3	(1.5)	2	(1)								
		Child satisfaction with life	3	(2)	3	(1)	3	(2)								
		Child attitudes towards antisocial behaviour	2	(1.5)	3	(1)	3	(1)								

Child mental distress								
Child overall mental health	2 (2)	1 (1)	1 (1)	9 (60)	6 (50)	8 (57)	23	3
Child anxiety	2 (1)	2 (1)	2 (1)	2 (13)	0 (0)	0 (0)	2	20
Child depression	2 (2.5)	3 (1)	2 (1.5)					
Child dissociation	2 (2)	3 (1.75)	4 (1)					
Child eating disorders	2 (2)	2 (1)	3 (2)					
Child hypervigilance	2 (1)	3 (1.75)	4 (2.5)	3 (20)	1 (8)	1 (7)	5	19
Child irritability	4 (2)	4 (1)	4 (1.75)					
Child post-traumatic stress	2 (1.5)	2 (0.75)	2 (2.75)	5 (33)	4 (33)	5 (36)	14	12=
Child self-harm	2 (2)	2 (1.75)	2 (1)					
Child substance use	3 (1.5)	3 (1)	2 (1)					
Child suicide	1 (1)	1 (0)	1 (1)	8 (53)	5 (42)	5 (36)	18	8=
Child trauma	1 (1)	2 (1)	1 (1)	8 (53)	8 (67)	9 (64)	25	1
**Caregiver health & wellbeing**								
Caregiver mental distress								
Caregiver overall mental health	2 (1.5)	2 (2)	2 (1)					
Caregiver anxiety	3 (1.5)	3 (2)	3 (2)					
Caregiver depression	3 (2)	3 (1)	2 (2)					
Caregiver dissociation	2 (2)	3 (1.75)	4 (1.25)					
Caregiver eating disorders	3 (2)	3 (1)	4 (2)					
Caregiver hypervigilance	2 (1)	3 (1)	4 (1)					
Caregiver irritability	4 (3)	4 (1)	4 (1.25)					
Caregiver post-traumatic stress	2 (1)	2 (1)	3 (2)					
Caregiver self-harm	3 (2.5)	2 (1)	3 (2)					
Caregiver substance use	2 (1)	2 (1)	3 (2)					
Caregiver suicide	2 (1.5)	1 (1)	2 (3)					
Caregiver trauma	2 (2)	2 (1.75)	2.5 (2.25)					
**Caregiver relationships & parenting**								
Caregiver-child relationship								
Parent-child relationship	2 (0)	1 (0.75)	1 (1)	5 (33)	12 (100)	8 (57)	24	2
Parent-child interaction	2 (1.75)	2 (2)	2 (2)					
Parent-child conflict	3 (1)	2 (1)	2 (1)					
Child's feelings about parents	2 (2)	2 (1.75)	3 (3)	7 (47)	7 (58)	3 (21)	17	9=
Relationship with birth family	4 (1)	3.5 (1.75)	3 (1)					
**Home environment & household**								
Family function								

Family functioning and stability	3	(1)	2	(0.75)	2	(1)								
Family relationships	2	(1)	2	(2)	2	(1)	5	(33)	9	(75)	7	(50)	21	5=
Family emotional environment	2	(2)	2	(1)	2	(1)								
Family communication	2	(1.5)	2	(1)	3	(2)								
Missing episodes	4	(1)	4	(1.5)	3	(1)								
**Social support & peer relationships**														
Child's relationships & social support														
Child social support	2	(2)	2	(1.75)	2	(1)	5	(33)	8	(67)	7	(50)	20	6=
Child's relationships with wider family, professionals etc.	3	(1.5)	3	(1.5)	3	(2)								
Child's relationships with peers	2	(1.5)	2	(1)	2	(1)								
Child has trusted relationships	1	(2)	1	(1)	2	(1.25)	6	(40)	7	(58)	8	(57)	21	5=
Connection with other survivors	3	(2)	4	(2)	4	(1)								
Understanding of healthy interpersonal relationships	3	(2)	3	(1)	3	(2.25)								
Ability to seek help	2	(2)	2	(1)	3	(1)								
Family relationships & social support														
Family support	3	(1.5)	2	(2)	2	(1.25)								
Family's relationships with friends	3	(1.5)	3	(2)	4	(1.5)								
Family's relationships with social services	3	(1.5)	4	(3)	3	(1)								
Family feels part of wider community	3	(1)	3	(1)	3.5	(1.25)								
Family social isolation	3	(1)	3	(1)	3	(1.25)								
Fear of social judgement	4	(1)	3	(2)	4	(2)								
**Safety, feelings & knowledge related to violence and abuse**														
Safety														
Caregiver ability to safety plan for self and child	3	(2)	1	(1.75)	2	(2)								
Freedom to go about daily life	2	(1)	2	(1.5)	3	(2)	4	(27)	4	(33)	1	(7)	9	16
Risk of harm	2	(2)	2	(1)	1	(1)	6	(40)	3	(25)	8	(57)	17	9=
Feelings of fear	2	(0.5)	2	(0.5)	3	(3)	6	(40)	2	(17)	4	(29)	12	13
Feelings of safety	2	(1)	2	(1)	2	(2)	7	(47)	8	(67)	4	(29)	19	7
Access to safe spaces	2	(2)	1	(1)	2	(2)								
Child's knowledge and understanding of safety	3	(2)	2	(1.5)	3	(2)								
Child's contact with harmful people														
Child's contact with harmful parent/caregiver	2	(1)	2	(2.75)	1	(2)								
Child's contact with other harmful adults	2	(2)	3	(3)	2	(3)								
**Violence, abuse, and maltreatment**														
Child maltreatment														
Child abuse and neglect	2	(1.5)	1	(1)	1	(1)	8	(53)	5	(42)	9	(64)	22	4

For peer review only - http://bmjopen.bmj.com/site/about/guidelines.xhtml

Child physical abuse	1	(1)	1.5	(1)	1	(1.25)	6	(40)	2	(17)	6	(43)	14	12=
Neglect of children by caregivers	2	(2)	1	(1)	1.5	(1)								
Child emotional abuse	1	(1)	1.5	(1.75)	1.5	(2)	8	(53)	2	(17)	5	(36)	15	11=
Child sexual abuse	1	(0.5)	1	(1)	1	(1)	8	(53)	4	(33)	6	(43)	18	8=
Child forced marriage	2	(2)	2	(2)	3	(2.5)								
Child sexual exploitation	1	(2.5)	1.5	(1)	2	(2)								
Child's exposure to DVA	2	(1)	1.5	(2)	2	(3)								
Child experience of witnessing violence and abuse	2.5	(3)	2	(1)	3	(3)								
Risk of re-traumatisation	2	(2)	2	(2)	1	(2.25)								
**Intervention outcomes**														
Intervention adverse effects														
Service harms	2	(1)	2	(1)	2	(2)	6	(40)	6	(50)	4	(29)	16	10
Long term negative impact of intervention	2	(2)	2	(1.5)	2	(1)	4	(27)	4	(33)	7	(50)	15	11=

For peer review only - http://bmjopen.bmj.com/site/about/guidelines.xhtml

Round 2 and 3 scoring: Domestic violence and abuse

			Round 2						Round 3							
			Survivor		Practitioner		Researcher		Survivor		Practitioner		Researcher			
Area	Domain	Outcome	Median	(IQR)	Median	(IQR)	Median	(IQR)	Score	(%)	Score	(%)	Score	(%)	TOTAL	RANK
**Child health & wellbeing**																
	Child wellbeing															
		Child behaviour	1	(0)	2	(1)	2	(0.5)	3	(27)	5	(29)	6	(55)	14	9=
		Child aggression	1	(1)	2	(2)	2.5	(1.25)								
		Child development	1	(3)	2	(1.5)	2	(1)								
		Child global functioning	3	(2.5)	2	(1.5)	2	(0.75)								
		Child speech and language	2	(3.5)	2	(1)	3	(1.75)								
		Child enuresis or soiling related to behaviour	2	(3.5)	3	(2)	3.5	(1.75)								
		Child emotional health & wellbeing	1.5	(1.75)	1	(1)	2	(1)								
		Child emotional understanding	1.5	(1.75)	2	(2)	3.5	(2)								
		Child ability to concentrate	2.5	(2.5)	3	(2)	3	(1)								
		Child executive functioning	2	(0.75)	2	(1)	3	(1.75)								
		Child anger	2	(2.5)	2	(2.5)	3.5	(1.75)								
		Child sense of calm	2	(1.75)	2	(2.5)	3	(2)								
		Child sense of panic	1	(1.75)	2	(2)	2.5	(1.75)								
		Child happiness	2	(2.5)	2	(2.5)	3	(2)								
		Child perception of recovery from trauma	1	(0.75)	2	(1)	2	(1.75)	2	(18)	7	(41)	1	(9)	10	13
		Child awareness of own body	1	(1)	1.5	(2)	3	(1.75)								
		Child overall wellbeing	2	(1.5)	2	(2)	3	(2)								
		Child satisfaction with life	1.5	(1.75)	2	(1.75)	3	(2)								
		Child attitudes towards antisocial behaviour	2	(2)	2	(3)	3	(1.75)								
	Child mental distress															
		Child overall mental health	1	(0.5)	1.5	(1)	2	(1)	7	(64)	4	(24)	7	(64)	18	5=
		Child anxiety	2	(1.5)	2.5	(2)	2	(1.5)								
		Child depression	2	(1)	2	(1.75)	2	(2)								
		Child dissociation	1	(2)	2	(1)	3	(1.5)								
		Child eating disorders	2	(2)	3	(1)	3	(1.5)								
		Child hypervigilance	1	(1.5)	2	(1.75)	3	(1.5)								
		Child irritability	3	(3)	3	(3.5)	4	(1.5)								
		Child post-traumatic stress	1	(1)	2	(1.75)	1	(1)	4	(36)	8	(47)	8	(73)	20	4
		Child self-harm	1	(1.5)	3	(2)	2	(2)								
		Child substance use	2	(1.5)	2.5	(1)	3	(1)								
		Child suicide	1	(0.5)	1	(2)	1	(1.5)								

For peer review only - http://bmjopen.bmj.com/site/about/guidelines.xhtml

Child trauma	1	(0)	2	(1)	1	(1.5)								
**Caregiver health & wellbeing**														
Caregiver wellbeing														
Caregiver general health & wellbeing	1.5	(1)	2.5	(1)	2	(1.75)	7	(64)	10	(59)	5	(45)	22	3=
Caregiver emotional health & wellbeing	1.5	(1)	2	(1)	2	(2)	3	(27)	8	(47)	4	(36)	15	8=
Caregiver emotional understanding	2	(0.5)	2.5	(1.25)	3	(2)								
Caregiver ability to concentrate	2	(1.25)	3	(2.25)	3	(1)								
Caregiver executive function	3	(1)	2.5	(2.25)	3	(2)								
Caregiver anger	2	(1)	3	(3)	3	(1.5)								
Caregiver sense of calm	2	(2)	2.5	(2.25)	4	(2)								
Caregiver happiness	1	(1.25)	2	(2)	3	(2)								
Caregiver perception of recovery from trauma	2	(2)	2.5	(3)	4	(1.75)								
Caregiver awareness of own body	1	(1.25)	2	(2.25)	3	(1)								
Caregiver satisfaction with life	2	(1.25)	2	(1)	2	(1)								
Caregiver engagement in risky behaviours	2	(1.25)	3	(3)	3	(2)								
Perpetrator health & wellbeing	5	(4)	3	(2.5)	4	(2)								
Caregiver mental distress														
Caregiver overall mental health	1	(0.5)	1	(1)	2	(1)	6	(55)	5	(29)	6	(55)	17	6
Caregiver anxiety	2	(1.5)	3	(1)	3	(1.5)								
Caregiver depression	2	(2)	2	(1)	2	(2)								
Caregiver dissociation	2	(2)	3	(1.5)	3	(1.5)								
Caregiver eating disorders	3	(2)	3	(2)	4	(1)								
Caregiver hypervigilance	2	(1.5)	3	(1.5)	3	(1)								
Caregiver irritability	2	(2.5)	4	(2.75)	4	(0.75)								
Caregiver post-traumatic stress	1	(1)	2	(2)	2	(2)								
Caregiver self-harm	1	(0.5)	3	(2.5)	3	(1.5)								
Caregiver substance use	2	(2)	2	(1.5)	3	(2)								
Caregiver suicide	1	(0)	1	(2)	2	(2)								
Caregiver trauma	1	(0)	1	(1)	2	(2.5)								
**Caregiver relationships & parenting**														
Caregiver-child relationship														
Parent-child relationship	1	(2)	2	(1)	2	(1)	7	(64)	14	(82)	8	(73)	29	2
Parent-child interaction	1	(1.5)	2	(1.5)	2	(1)								
Parent-child conflict	2	(1)	3	(1.5)	3	(1)								
Child's feelings about parents	2	(1)	2	(1.5)	2	(1)								
Relationship with birth family	4	(1)	4	(2.5)	3	(1.5)								
**Home environment & household**														
Family function														
Family functioning and stability	2	(2)	2	(2)	3	(1)	6	(55)	11	(65)	5	(45)	22	3=
Family relationships	2	(1.5)	1.5	(1)	2	(1)	3	(27)	11	(65)	4	(36)	18	5=
Family emotional environment	1	(1)	2	(1.25)	3	(1.75)	4	(36)	8	(47)	3	(27)	15	8=
Family communication	2	(1)	2.5	(3)	3	(1.75)								

Missing episodes	4	(1)	4	(2.75)	3	(1)								
**Safety, feelings & knowledge related to violence and abuse**														
Safety														
Caregiver ability to safety plan for self and child	2	(1.25)	2	(1.5)	2	(2)								
Freedom to go about daily life	1	(0.25)	2	(2)	2	(2)	4	(36)	6	(35)	4	(36)	14	9=
Risk of harm	1	(1)	1	(1)	1	(1)	8	(73)	13	(76)	9	(82)	30	1=
Feelings of fear	1	(1)	2	(1.5)	2	(1.25)	2	(18)	6	(35)	4	(36)	12	11
Feelings of safety	1	(0.5)	1	(1)	1.5	(2)	7	(64)	15	(88)	8	(73)	30	1=
Access to safe spaces	1	(1)	2	(1)	1.5	(1)	5	(45)	4	(24)	4	(36)	13	10
Child's knowledge and understanding of safety	1.5	(1.25)	2	(1)	2	(2)								
Child's thoughts & knowledge about their experiences of CM or DVA														
Child knows DVA is not their fault	1	(0.5)	1	(1)	1	(1)	3	(27)	6	(35)	2	(18)	11	12
Child's ability to make sense of DVA experiences	1	(2)	2	(2)	2.5	(1)								
Child feels validated	2	(2)	1	(2)	2	(2)								
Child's sense that they can move on from violence	1	(1)	1	(1.25)	1.5	(1)	4	(36)	2	(12)	2	(18)	8	15=
Child's ability to identify safe relationships	1	(2)	2	(1.25)	1	(1)								
Child's perception of others' judgements of abuse	2	(1.5)	3	(2.25)	4	(1.75)								
Child's knowledge about DVA	1	(0.5)	1	(1.25)	2	(0.75)	7	(64)	4	(24)	5	(45)	16	7
Child's understanding of impact of violence	2	(2)	2	(2)	3	(1.75)								
Child's understanding of consent	1	(0.5)	2	(1)	2	(2)	4	(36)	2	(12)	3	(27)	9	14
**Violence, abuse, and maltreatment**														
Child maltreatment														
Child abuse and neglect	1	(0)	2	(1)	1	(1.25)	6	(55)	6	(35)	6	(55)	18	5=
Child physical abuse	1	(1)	1.5	(1.25)	1.5	(2)								
Neglect of children by caregivers	1	(0.5)	1.5	(1.25)	1	(1)								
Child emotional abuse	1	(0.5)	1	(1)	2	(2)	2	(18)	4	(24)	2	(18)	8	15=
Child sexual abuse	1	(0)	1	(2.25)	1	(1)								
Child forced marriage	1	(0.5)	2	(2.5)	1	(2)								
Child sexual exploitation	1	(0)	2	(2.25)	1	(1.25)								
Child's exposure to DVA	1	(0)	1	(0)	2	(1.25)								
Child experience of witnessing violence	1	(1)	1.5	(1)	2	(2)	5	(45)	6	(35)	4	(36)	15	8=
Risk of re-traumatisation	1	(0)	2	(1)	2	(3)								
Domestic violence and abuse between caregivers														
Domestic violence and abuse	1	(0)	1	(0)	1	(1)								
Economic abuse	1	(1)	2	(1.5)	2	(2)								
Emotional abuse	1	(0)	1	(2)	1.5	(1.25)								
Physical abuse	1	(1)	2	(2)	1	(1)								
Sexual abuse	1	(0)	2	(2)	1	(1.25)								

For peer review only - http://bmjopen.bmj.com/site/about/guidelines.xhtml

Coercive control	1	(0)	1	(1.5)	1	(1)
Experience of harassment	1	(1)	2	(2)	2	(1)
Harm to family pets	1	(2)	3	(2.5)	2	(1)
Injury	1	(0)	2	(2.5)	2	(1)
Reproductive coercion	1	(1.5)	2	(2)	2	(1.25)
Perpetrator rehabilitation/desistance	1	(3)	1	(2)	2	(1)
Perpetration of DVA	1	(1.5)	1	(2)	1.5	(1)
Risk of re-traumatisation	1	(1)	2	(2)	2	(1.25)
**Intervention outcomes**						
Intervention adverse effects						
Service harms	1	(0.25)	2	(1.5)	2	(0.25)
Long term negative impact of intervention	1.5	(2)	2	(3)	2	(1.5)

For peer review only

For peer review only - http://bmjopen.bmj.com/site/about/guidelines.xhtml

Supplement 9: DVA consensus workshop consensus statement

Domestic Violence Core Outcome Set consensus statement

Following the multi-stakeholder workshop on 28th September 2021 the workshop participants agreed to change the outcome *child overall mental health* to *child emotional health and wellbeing* which was part of the original longlist of candidate outcomes. This decision emerged from discussions that emphasised concerns around: i) the medicalisation of young people's distress in response to experiences of DVA; ii) the consensus that emotional health and well-being more readily captures positive functioning and/or adaption rather than emphasising deficits (that some participants felt were implicit in the term *mental health);* iii) the broader applicability of emotional health and wellbeing to sectors outside of health care: the voluntary sector, education and social care. It was agreed that emotional health and wellbeing could encompass specific mental health outcomes such as psychiatric symptoms and diagnoses.

Original outcome

- **Overall mental health** (includes psychiatric symptoms, psychological distress, psychological wellbeing, mental well-being, mental health problems, signs of these including admissions/healthcare or service contacts)

Proposed change:

- **Emotional health & wellbeing** (includes emotions, mood, internalizing problems or symptoms; emotional regulation; emotional security; emotional numbness)

view only

Supplement 10: Consensus workshop outcome rankings

Child maltreatment consensus workshop outcome rankings by number of votes

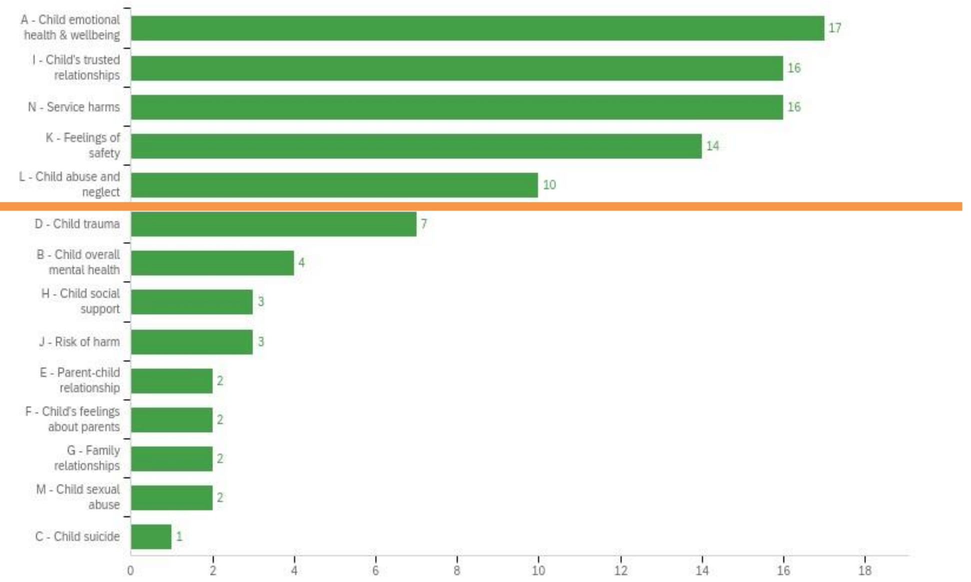

Domestic violence and abuse consensus workshop outcome rankings by number of votes

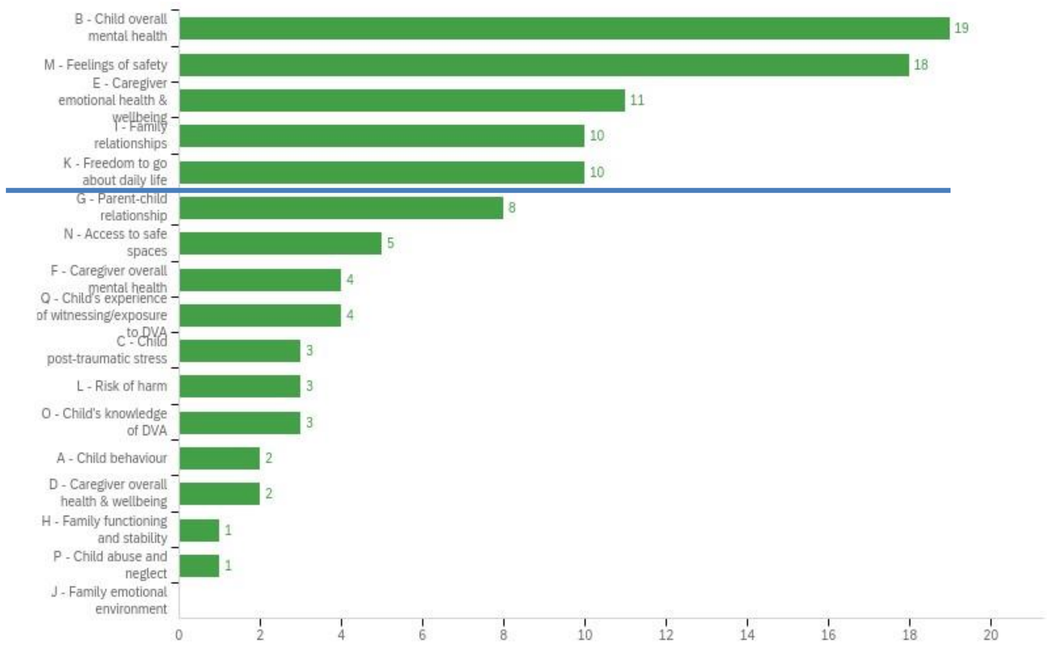

Supplement 11: Summary of discussion about implications of COS for underserved groups

1) Language and meaning

The meaning of concepts related to outcomes was discussed and whether these are understood in the same way by service users and professionals. There was concern about how language could be adapted for all age groups of children without loss of meaning. The cross-cultural understanding of concepts was raised and how accurate translation can be ensured across global settings. Finally, there was discussion about gender in relation to how child behaviour is understood and how violence is analysed.

2) Rights and discrimination

Overall, there was a preference for a rights-based focus as well as understanding of inequalities in any outcome measurement tool. Participants reflected on the impact of socio-economic exclusion and destitution and how particularly minoritised groups, such as migrant children, could be protected. There was a focus on how to centre the child's voice in any understanding of outcomes. In terms of outcome measurement/assessment there was discussion around how to reduce practitioner bias against marginalised groups, including the misuse of mental health diagnosis.

3) Using measurement tools

Concerns on the practical delivery of any measures used for the core outcome sets, both in terms of the tools themselves and the staff who might be administering the tools.

For peer review only - http://bmjopen.bmj.com/site/about/guidelines.xhtml

Core Outcome Set-STandards for Reporting: The COS-STAR Statement Checklist

SECTION/TOPIC	ITEM No.	CHECKLIST ITEM	REPORTED ON PAGE NUMBER
TITLE/ABSTRACT			
Title	1a	Identify in the title that the paper reports the development of a COS	1
Abstract	1b	Provide a structured summary	2
INTRODUCTION			
Background and Objectives	2a	Describe the background and explain the rationale for developing the COS.	3
	2b	Describe the specific objectives with reference to developing a COS.	3
Scope	3a	Describe the health condition(s) and population(s) covered by the COS.	3
	3b	Describe the intervention(s) covered by the COS.	3
	3c	Describe the setting(s) in which the COS is to be applied.	3
METHODS			
Protocol/Registry Entry	4	Indicate where the COS development protocol can be accessed, if available, and/or the study registration details.	4
Participants	5	Describe the rationale for stakeholder groups involved in the COS development process, eligibility criteria for participants from each group, and a description of how the individuals involved were identified.	4
Information Sources	6a	Describe the information sources used to identify an initial list of outcomes.	5
	6b	Describe how outcomes were dropped/combined, with reasons (if applicable).	5-6
Consensus Process	7	Describe how the consensus process was undertaken.	6-7
Outcome Scoring	8	Describe how outcomes were scored and how scores were summarised.	6-7, online supplementary materials
Consensus Definition	9a	Describe the consensus definition.	6-7, online supplementary materials
	9b	Describe the procedure for determining how outcomes were included or excluded from consideration during the consensus process.	6-7, online supplementary materials
Ethics and Consent	10	Provide a statement regarding the ethics and consent issues for the study.	4,5,6
RESULTS			
Protocol Deviations	11	Describe any changes from the protocol (if applicable), with reasons, and describe what impact these changes have on the results.	7, online supplementary materials

For peer review only

Participants	12	Present data on the number and relevant characteristics of the people involved at all stages of COS development.	Online supplementary materials
Outcomes	13a	List all outcomes considered at the start of the consensus process.	Link to longlist in repository
	13b	Describe any new outcomes introduced and any outcomes dropped, with reasons, during the consensus process.	7
COS	14	List the outcomes in the final COS.	8
DISCUSSION			
Limitations	15	Discuss any limitations in the COS development process.	9
Conclusions	16	Provide an interpretation of the final COS in the context of other evidence, and implications for future research.	9-10
OTHER INFORMATION			
Funding	17	Describe sources of funding/role of funders.	10
Conflicts of Interest	18	Describe any conflicts of interest within the study team and how these were managed.	10

From: Kirkham JJ, Gorst S, Altman DG, Blazeby JM, Clarke M, Devane D, et al. (2016) Core Outcome Set–STAndards for Reporting: The COS-STAR Statement. PLoS Med 13(10): e1002148. https://doi.org/10.1371/journal.pmed.1002148

For peer review only
